# Glucose transporter 1-mediated glucose uptake is limiting for B-cell acute lymphoblastic leukemia anabolic metabolism and resistance to apoptosis

**DOI:** 10.1038/cddis.2014.431

**Published:** 2014-10-16

**Authors:** T Liu, R J Kishton, A N Macintyre, V A Gerriets, H Xiang, X Liu, E D Abel, D Rizzieri, J W Locasale, J C Rathmell

**Affiliations:** 1 Department of Pharmacology and Cancer Biology, Duke University, Durham, NC 27710, USA; 2 Department of Immunology, Duke Cancer Institute and Duke Molecular Physiology Institute, Duke University, Durham, NC 27710, USA; 3 Division of Nutritional Sciences, Cornell University, Ithaca, NY 14850, USA; 4 Fraternal Order of Eagles Diabetes Research Center, Division of Endocrinology and Metabolism, Department of Medicine, Carver College of Medicine University of Iowa, Iowa City, IA 52242, USA; 5 Division of Cell Therapy, Department of Medicine, Duke University Medical Center, Durham, NC 27710, USA

## Abstract

The metabolic profiles of cancer cells have long been acknowledged to be altered and to provide new therapeutic opportunities. In particular, a wide range of both solid and liquid tumors use aerobic glycolysis to supply energy and support cell growth. This metabolic program leads to high rates of glucose consumption through glycolysis with secretion of lactate even in the presence of oxygen. Identifying the limiting events in aerobic glycolysis and the response of cancer cells to metabolic inhibition is now essential to exploit this potential metabolic dependency. Here, we examine the role of glucose uptake and the glucose transporter Glut1 in the metabolism and metabolic stress response of BCR-Abl+ B-cell acute lymphoblastic leukemia cells (B-ALL). B-ALL cells were highly glycolytic and primary human B-ALL samples were dependent on glycolysis. We show B-ALL cells express multiple glucose transporters and conditional genetic deletion of Glut1 led to a partial loss of glucose uptake. This reduced glucose transport capacity, however, was sufficient to metabolically reprogram B-ALL cells to decrease anabolic and increase catabolic flux. Cell proliferation decreased and a limited degree of apoptosis was also observed. Importantly, Glut1-deficient B-ALL cells failed to accumulate *in vivo* and leukemic progression was suppressed by Glut1 deletion. Similarly, pharmacologic inhibition of aerobic glycolysis with moderate doses of 2-deoxyglucose (2-DG) slowed B-ALL cell proliferation, but extensive apoptosis only occurred at high doses. Nevertheless, 2-DG induced the pro-apoptotic protein Bim and sensitized B-ALL cells to the tyrosine kinase inhibitor Dasatinib *in vivo*. Together, these data show that despite expression of multiple glucose transporters, B-ALL cells are reliant on Glut1 to maintain aerobic glycolysis and anabolic metabolism. Further, partial inhibition of glucose metabolism is sufficient to sensitize cancer cells to specifically targeted therapies, suggesting inhibition of aerobic glycolysis as a plausible adjuvant approach for B-ALL therapies.

Many cancer cells have elevated rates of glycolysis and lactate production even in the presence of oxygen. This program, termed aerobic glycolysis, occurs in a wide range of both solid and liquid tumors and is driven by oncogenic signals and microenvironmental pressures.^1^ Aerobic glycolysis is proposed to allow metabolism in low oxygen tensions and to provide biosynthetic intermediates for cell growth. Indeed, aerobic glycolysis readily supports both generation of ATP and biosynthesis of lipids, nucleic acids, and amino acids.^1^ Given the high rates of glucose consumption and aerobic glycolysis in most cancers, targeting glucose metabolism has become of significant interest as an approach to eliminate cancer cells. It is now important to establish mechanisms of aerobic glycolysis and the response of cancer cells to metabolic inhibition.

The *t*(9;22) chromosomal translocation that generates the oncogenic kinase BCR-Abl occurs in ~25% of adult B-cell acute lymphoblastic leukemia cells (B-ALL) and is associated with poor prognosis.^2^ The metabolic program of B-ALL cells is undefined, although diffuse large B-cell lymphoma (DLBCL) can either be highly glycolytic or use oxidative phosphorylation and mitochondrial metabolism.^3^ It has been suggested that BCR-Abl signaling is associated with elevated glucose metabolism, as BCR-Abl can promote glucose uptake and trafficking of glucose transporter Glut1 to the cell surface. Conversely, inhibition of BCR-Abl in leukemic cells suppresses glucose uptake and glycolysis.^4, 5, 6, 7^ This regulation of glucose metabolism may be critical for survival of BCR-Abl B-ALL, as enforced expression of Glut1 protected B-ALL cells from imatinib-induced apoptosis.^8^ These data show that BCR-Abl promotes glucose uptake and aerobic glycolysis, and BCR-Abl-transformed cells may rely on this pathway.

Targeting glucose metabolism can have efficacy against a variety of cancers.^9^ Mechanistic understanding of cancer cell metabolic requirements or response to inhibition using pharmacologic approaches, however, has been limited. It has been shown using the glycolytic inhibitor 2-deoxyglucose (2-DG) or glucose deprivation culture conditions that inhibition of glucose metabolism impacts cancer cell growth and viability through several different mechanisms, including cell cycle arrest or cell death by activating AMPK pathway and inactivating mTOR signaling.^10^ Reduced glucose metabolism has also been found to impact the stability and synthesis of Bcl-2 family proteins. Glucose deprivation induces expression of pro-apoptotic molecules, including Bim^7,11, 12, 13, 14, 15^ and can induce apoptosis in cells transformed with oncogenic K-Ras through the unfolded protein response pathway.^16^

Here we examine the mechanism and role of glucose uptake in B-ALL metabolism and leukemia progression by genetically targeting glucose transport. The Glut family of hexose transporters consists of 14 members^17^ and B-ALL cells expressed multiple family members. Conditional deletion of Glut1, however, demonstrated that B-ALL cells are reliant on this specific glucose transporter to sustain anabolic metabolism, proliferation, and resistance to cell death. Consistent with our data showing a key role for glucose uptake, we found that pharmacologic inhibition of glycolysis sensitized B-ALL cells to caspase activation and apoptosis to reduce leukemia burden *in vivo*. Glut1 and glucose uptake have a key role, therefore, to maintain BCR-Abl B-ALL cell growth and resistance to cell death.

## Results

### B-ALL cells utilize glucose through aerobic glycolysis

**Figure 1:**
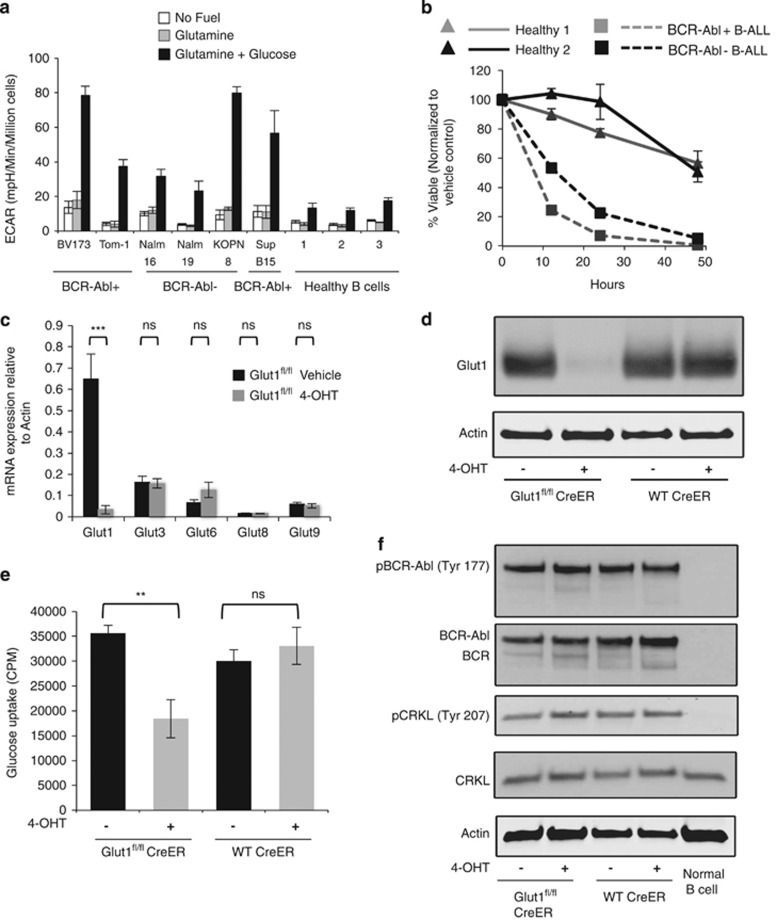
B-ALL cells require glycolysis and express multiple glucose transporters, but Glut1 is limiting for maximal glucose uptake. (**a**) ECAR was measured in human B-ALL cell lines and primary healthy-donor peripheral blood B cells at baseline and followed by sequential injection of 2 mM glutamine and 25 mM glucose. (**b**) Viability of human primary B-ALL cells and normal B cells from two donors each over time upon treatment of 2-DG (10 mM). Values were normalized at each time point to the vehicle control for that sample. The BCR-Abl status of primary B-ALL is indicated. (**c**–**e**) Control and Glut1^fl/fl^ CreER B-ALL cells were treated with vehicle or 4-OHT for 96 h followed by 48 h culture without 4-OHT prior to analyses. (**c**) Glucose transporter family member mRNA expression was measured by qrtPCR. Shown are expression of detected glucose transporters relative to expression of beta-actin. Other Glut family members were not detected. (**d**) Glut1 protein was measured by immunoblot and (**e**) glucose uptake was measured with radiolabeled glucose. (**f**) BCR-Abl activity was analyzed by immunoblot. Means of three or more replicates and S.D. are shown ***P≤0.001, **P≤0.005. NS, not significant

Oncogenic signals can increase glucose uptake and drive aerobic glycolysis.^18^ However, usage of glucose in B-ALL and the response of B-ALL cells to inhibition of aerobic glycolysis are poorly understood. To examine glucose metabolism activity in B-ALL, real-time extracellular flux was measured in six human B-ALL cell lines, including BCR-Abl+ and BCR-Abl− cell lines, and peripheral B cells from three healthy donors in the absence of nutrients, after addition of glutamine, then upon addition of glucose. Extracellular acidification rate (ECAR), reflective of lactate production, was similar in each cell type in the absence of nutrients (Figure 1a). Glutamine, which is used as another important metabolic fuel for many types of cancer cells in addition to glucose,^18^ did not increase ECAR in any of the B-ALL cell lines or normal B cells. The addition of glucose following glutamine, however, led to a sharp increase in ECAR in all the B-ALL cells (Figure 1a). A modest increase in ECAR was also observed in normal B cells. Importantly, under no condition did the oxygen consumption rate (OCR) change with glutamine or glucose addition (Supplementary Figure 1a), and we did not observe a clear difference of metabolic program between BCR-Abl+ and BCR-Abl− cell lines. B-ALL cells thus selectively metabolize glucose through aerobic glycolysis with minimal contribution to oxidative metabolism.

To test whether primary B-ALL cells depended on maintenance of high glycolysis, BCR-Abl+ and BCR-Abl− B-ALL patient samples and two normal donor B cells were cultured in complete media with or without the addition of 10 mM 2-DG to strongly inhibit glycolysis. Cell survival was measured over time and normal B cells were found only modestly affected by glycolytic inhibition (Figure 1b). In contrast, primary B-ALL cells treated with 2-DG rapidly underwent cell death and were highly dependent on glycolysis regardless of the BCR-Abl status.

### B-ALL cells express multiple Glut family members and deletion of Glut1 partially inhibits glucose uptake

Glucose uptake is the first essential step in glucose metabolism and can be limiting in lymphocyte proliferation.^19, 20, 21^ Measurement of Glut family members in BCR-Abl+ murine B-ALL found expression of Glut1, Glut3, Glut6, Glut8, and Glut9, whereas the remaining Glut family members were undetected (Figure 1c). Similarly, human B-ALL cell lines expressed a variable array of Glut family members, including Glut1, Glut3, Glut4, Glut5, Glut6, Glut7, Glut8, and Glut9 (Supplementary Figure 1B). Of these transporters, Glut1 was previously shown important for proliferation and antibody secretion of normal B cells^19^ and appeared broadly abundant in B-ALL. To genetically target B-ALL glucose uptake, therefore, we generated primary BCR-Abl B-ALL cells from Glut1^fl/fl^/Ubi-Cre-ER^T2^ mice to enable inducible deletion of glucose transporter Glut1 upon treatment with 4-hydroxytamoxifen (4-OHT). Treatment of Glut1^fl/fl^/Ubi-Cre-ER^T2^ B-ALL with 4-OHT led to efficient deletion of Glut1 with minimal compensation from other Glut transporters, and 4-OHT had no effect on control B-ALL cells (Figures 1c and d, Supplementary Figure 2). Deletion of Glut1 reduced glucose uptake by approximately half, showing Glut1 was limiting for maximal but does not mediate all glucose uptake (Figure 1e). To test whether this partial impairment of glucose transport impacted BCR-Abl oncogenic activity, we examined phosphorylation of BCR-Abl and immediate downstream targets and found BCR-Abl and its substrate CRKL remained phosphorylated (Figure 1f). Glut1, therefore, contributes significantly to B-ALL glucose uptake, and reduction of glucose metabolism does not alter BCR-Abl activity.

### Glut1 deficiency suppresses anabolic metabolism

**Figure 2:**
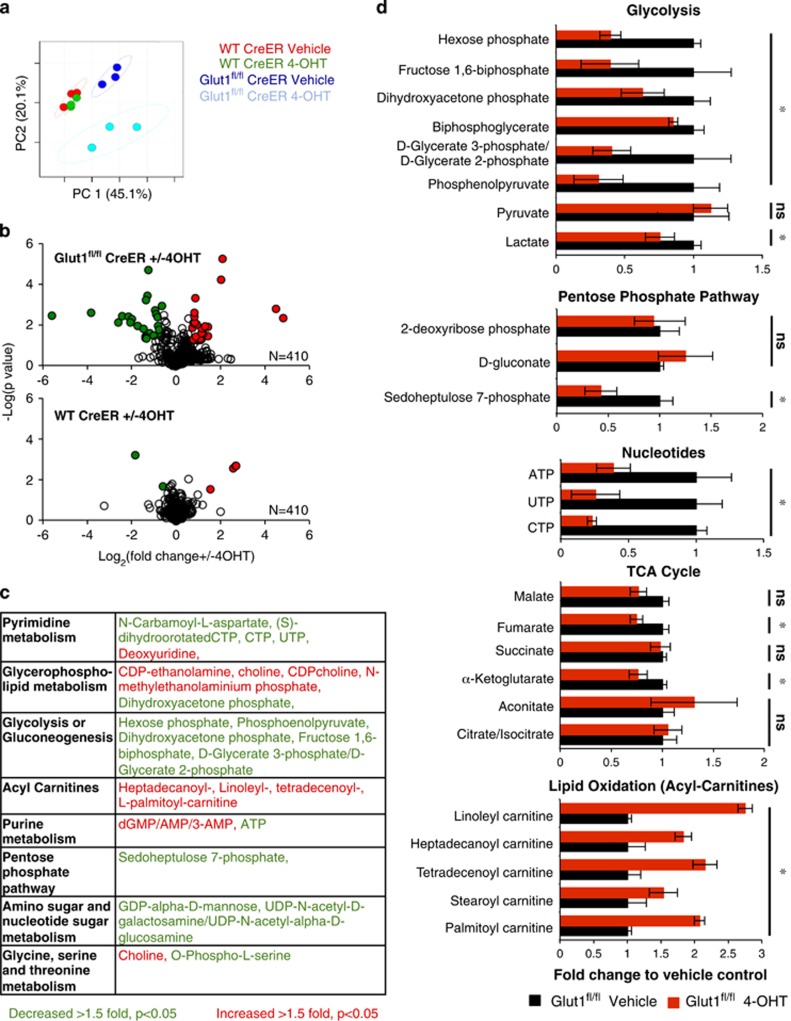
*In vitro* Glut1 deletion leads to metabolic reprogramming of B-ALL cells. (**a**–**c**) Steady-state metabolite levels in wild-type (WT) Cre-ER and Glut1^fl/fl^ CreER B-ALL cells treated with vehicle or 4-OHT were determined using LC/MS. (**a**) Principal component, (**b**) volcano plots of metabolites changed >1.5-fold, *P*<0.05, and (**c**) key metabolites categorized by KEGG metabolic pathways are shown in order of significance. (**d**) Relative levels of specific metabolites in key pathways are shown following Glut1 deletion. Data represent mean and S.D. for triplicate samples. *P<0.05. NS, not significant

Given the remaining capacity for glucose uptake in Glut1-deficient cells, the metabolic impact of Glut1 deletion was unclear. To better understand the metabolic role of Glut1, the steady-state levels of 410 metabolites were measured through non-targeted LC/MS^22^ in B-ALL cells, with or without expression of Glut1. Principal component analyses of the metabolome of each population showed a modest difference between control CreER^T2^ B-ALL cells and Glut1^fl/fl^ CreER^T2^ cells. However, control CreER^T2^ B-ALL were largely unaffected by 4-OHT treatment, whereas Glut1 deletion led to a distinct metabolic profile in 4-OHT-treated Glut1^fl/fl^ CreER^T2^ B-ALL cells (Figure 2a and Supplementary Table 1). Only five metabolites were significantly altered after 4-OHT treatment of control CreER^T2^ B-ALL cells, whereas 43 metabolites were specifically changed in response to Glut1 deletion (Figure 2b and Supplementary Table 1). Affected metabolites represented a wide array of pathways (Figure 2c) with particular reductions in anabolic pathways, including pyrimidine and purine metabolism, glycolysis, and the amino and nucleotide sugar metabolism that support glycosylation. Metabolic levels were lowered for glycolytic intermediates, a key pentose phosphate pathway intermediate, and multiple nucleotides and intermediates in nucleotide synthesis (Figure 2d, Supplementary Figure 3 and Supplementary Table 1). In contrast, catabolic pathways including glycerophospholipid metabolism and acyl-carnitines, which reflect fatty acid and lipid oxidation, were increased (Figure 2d). Surprisingly, pyruvate and lactate, the majority of TCA cycle intermediates, and free intracellular amino acids were unaffected by Glut1 deletion (Figure 2d, Supplementary Figure 3 and Supplementary Table 1). Pyruvate and lactate can be derived from non-glucose sources, such as glutamine, and this may account for maintenance of these metabolites even with reduced glucose metabolism.^23^ These data suggest that Glut1-dependent glucose mainly supports biosynthetic pathways in B-ALL cells, and reduced glucose uptake led to metabolic reprogramming to favor catabolism.

**Figure 3:**
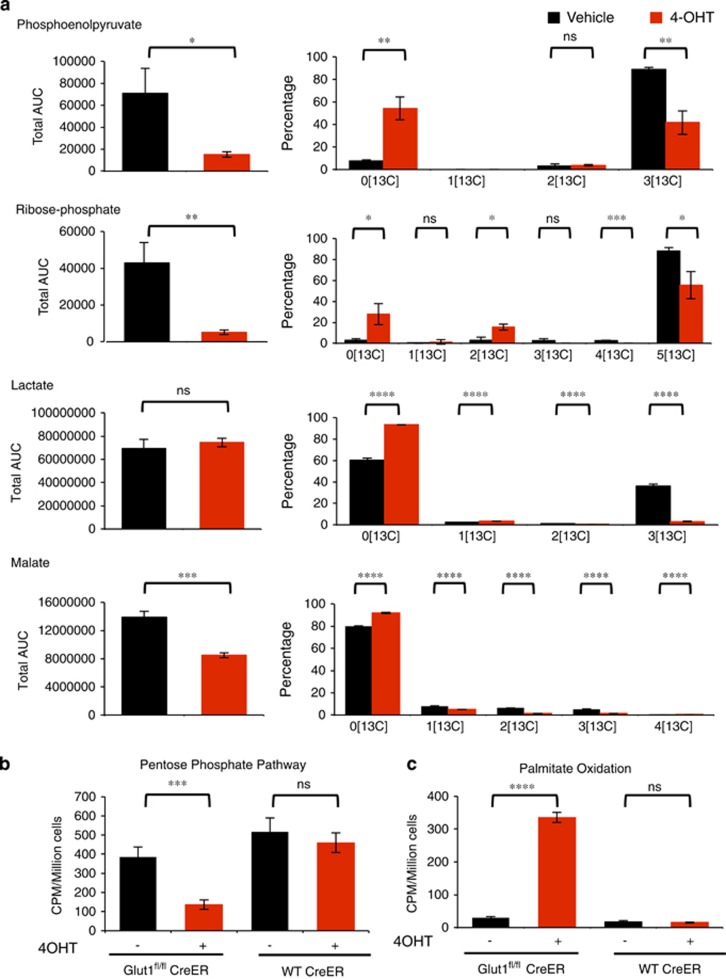
Glut1 deletion suppresses glucose contribution to anabolic pathways and increases catabolic metabolism. (**a**) 13C-glucose tracing contribution of glucose to indicated metabolite pools. Graphs on the left indicate the total quantity of each metabolite and the graph on the right indicates the relative distribution of ^13^C carbons in each metabolite. (**b** and **c**) WT Cre-ER and Glut1^fl/fl^ Cre-ER B-ALL cells were treated with vehicle or 4-OHT for 96 h followed by 48 h of culture without 4-OHT and (**b**) pentose phosphate pathway flux was measured by oxidation of 1-C^14^-glucose and (**c**) lipid oxidation was measured by oxidation of C^14^ palmitate. Means and S.D. of triplicate measurements are shown. *P<0.05, **P<0.005, ***P<0.001, ****P<0.0001. NS, not significant

To further investigate glucose contribution to downstream metabolic pathways and how Glut1 deficiency alters these pathway activities, glucose fate was traced and metabolic flux analysis was performed using ^13^C-labeled glucose. B-ALL cells were cultured in vehicle or 4-OHT for 4 days to delete Glut1 and then labeled with 13C-glucose for 24 h prior to LC/MS mass spectrometry. Despite partial maintenance of glucose uptake, flux to anabolic pathways was sharply curtailed following Glut1 deletion. Control Glut1-expressing cells efficiently converted 13C-glucose to uniformly labeled ^13^C phosphoenolpyruvate, dihydroxyacetone phosphate, and ribose phosphate through glycolysis and the pentose phosphate pathway, respectively (Figure 3a, Supplementary Figure 4 and Supplementary Table 2). Glut1-deficient cells, however, produced very little total levels of these metabolites relative to control cells and that which was generated contained a significantly lower fraction of 13C-glucose-derived carbon (Figures 2d and 3a, Supplementary Figure 4 and Supplementary Table 2). Pyruvate and lactate were present in similar levels, and control cells generated these metabolites through both ^13^C-labeled glucose and unlabeled sources, whereas the majority of these metabolites were derived from non-glucose sources in Glut1-deficient cells. Thus alternative sources, such as glutamine,^23^ contribute significantly to pyruvate and lactate in control cells and these pathways become increasingly dominant after Glut1 deletion. Surprisingly, glucose did not contribute significantly to the TCA cycle in B-ALL regardless of Glut1 expression, as malate, citrate, succinate, and alpha-ketoglutarate were unlabeled in both control and Glut1-deficient cells (Figure 3a, Supplementary Figure 4 and Supplementary Table 2). Thus, glucose was not the main fuel resource for oxidative metabolism in B-ALL, nor was it redirected toward oxidative metabolism in Glut1-deficient B-ALL cells. Rather, other metabolic fuels sustained the TCA cycle.

Radiolabeled tracer assays were next conducted in pentose phosphate and lipid oxidation pathways to independently confirm these findings. Consistent with previous results, pentose phosphate pathway activity was significantly reduced following Glut1 deletion (Figure 3b). Conversely, Glut1 deletion led to a sharp increase in palmitate oxidation (Figure 3c). Together, these data show that B-ALL cells are highly glycolytic and primarily use glucose to support biosynthetic reactions and pathways, such as the pentose phosphate pathway.

### Metabolic reprogramming suppresses B-ALL proliferation

**Figure 4:**
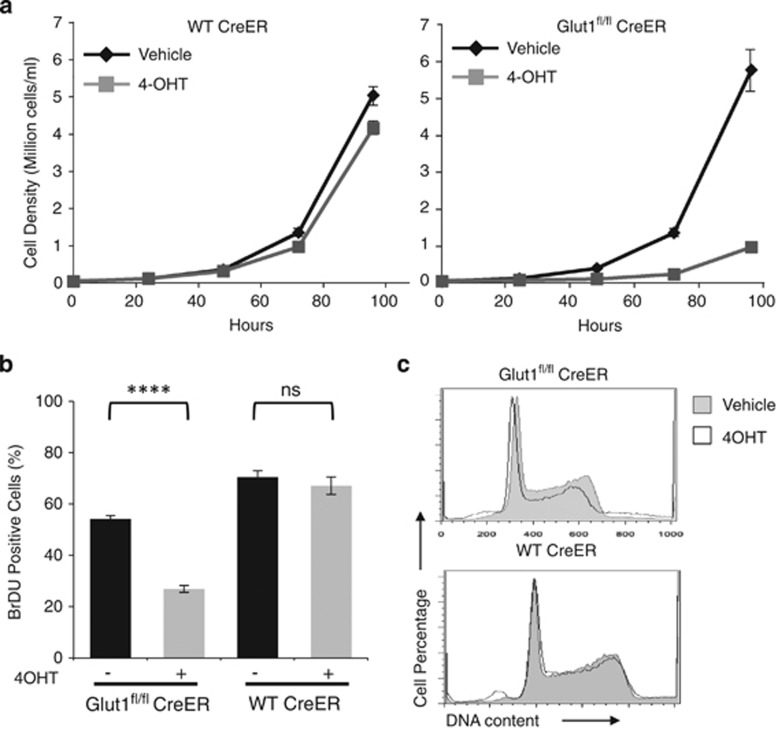
*In vitro* Glut1 suppresses B-ALL accumulation through decreased proliferation. (**a**) WT Cre-ER and Glut1^fl/fl^ Cre-ER B-ALL cells were treated with vehicle or 4-OHT for 96 h followed by 48 h of culture without 4-OHT, and cell numbers were counted over time. (**b** and **c**) After 4 days treatment with vehicle or 4-OHT followed by 2 days culture without 4-OHT, cells were cultured with BrDU for 1.5 additional hours and (**b**) BrDU incorporation was measured by intracellular flow cytometry. (**c**) DNA content was determined flow cytometrically by propidium iodide staining to indicate cell cycle status. Means and S.D. are shown for triplicate samples in representative experiments repeated three or more times. ****P<0.0001. NS, not significant

The sharp decrease in flux toward biosynthetic metabolic pathways and increased catabolism following Glut1 deletion suggested that Glut1 deficiency may impede B-ALL cell growth and proliferation. Indeed, 4-OHT treatment led to a sharp reduction in cell accumulation rates over time (Figure 4a). This was at least partially due to reduced proliferation, as BromodeoxyUridine (BrDU) incorporation in Glut1^fl/fl^ CreER^T2^ B-ALL cells was significantly decreased, whereas control CreER^T2^ cells were unaffected by 4-OHT (Figures 4a and b). Cell cycle profiling of control and 4-OHT-treated B-ALL cells, however, showed that Glut1 deletion did not lead to a clear cell cycle arrest or accumulation of cells in a specific phase of the cell cycle (Figure 4c). Rather Glut1 deficiency appeared to slow cell proliferation than activate a specific cell cycle arrest checkpoint.

### Glut1 deletion sensitizes B-ALL cells to apoptosis with targeted agents

**Figure 5:**
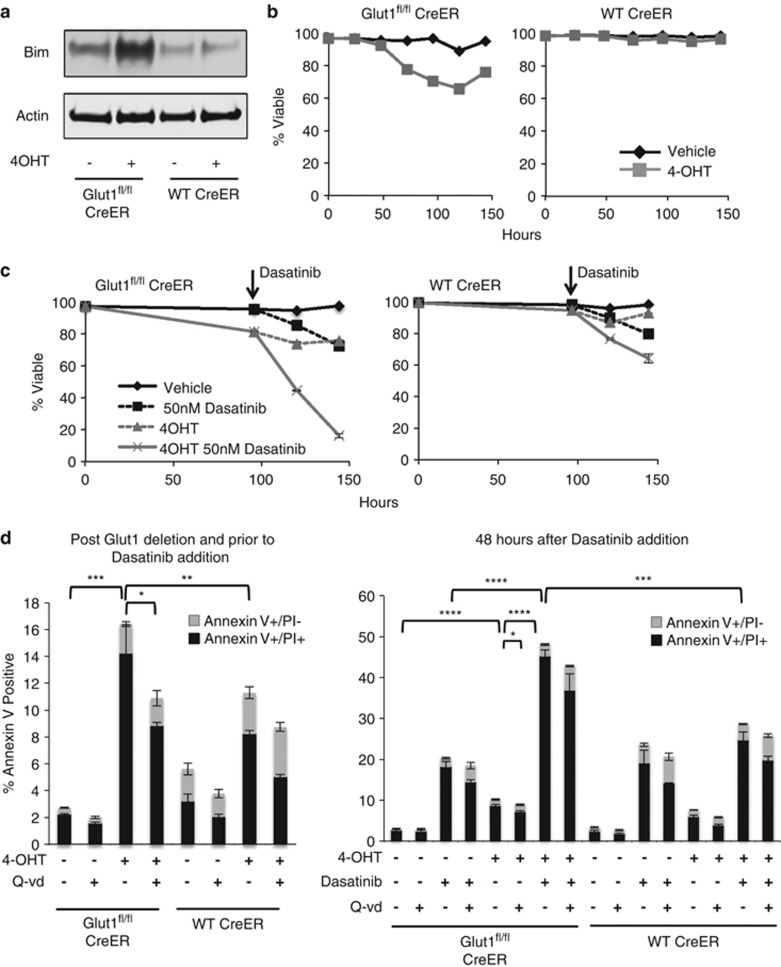
*In vitro* Glut1 deletion induces pro-apoptotic Bim expression and sensitizes B-ALL to cell death stimulus. (**a** and **b**) WT Cre-ER and Glut1^fl/fl^ Cre-ER B-ALL cells treated with vehicle or 4-OHT for 96 h followed by 48 h of culture without 4-OHT and (**a**) examined by immunoblot on day 6 or (**b**) analyzed by flow cytometry for survival over time. (**c**) WT Cre-ER and Glut1^fl/fl^ Cre-ER B-ALL cells were cultured with vehicle or 4-OHT for 96 h, washed, then cultured an additional 48 h alone or with addition of Dasatinib (50 nM), and cell viability was determined over time by flow cytometry. (**d**) Apoptosis in Glut1-deleted cells with or without Dasatinib treatment was assessed by annexin V/PI staining. Cells were treated with vehicle or 4-OHT for 96 h and apoptosis was assessed by annexin V/PI staining (left panel). After 96 h of culture with vehicle or 4-OHT, cells were washed and cultured for an additional 48 h alone or with Dasatinib (50 nM). Cell apoptosis was assessed at the end of the 48 h (right panel). Ten *μ*M pan caspases inhibitor Q-vd-oph was added in some cell cultures as indicated. Gray bar, annexin V+/PI− cell percentage. Black bar, annexin V+/PI+ cell percentage. Means and S.D. are shown for triplicate samples from representative experiments repeated three or more times. *P<0.05, **P<0.005, ***P<0.001, ****P<0.0001

Glucose metabolism is closely linked to cell survival, and Glut1 deletion may have also impaired cell accumulation *in vitro* through increased apoptosis. Consistent with this notion, Glut1 deficiency specifically induced expression of the pro-apoptotic protein Bim and only modestly impacted expression of other Bcl-2 family proteins, including pro-apoptotic protein Bax, Bak, Bid and anti-apoptotic protein Mcl-1 and Bcl-xL (Figure 5a and Supplementary Figure 5A). Bim can be induced in response to ER stress and the unfolded protein response,^24^ but only very modest markers of these pathways were detected relative to those induced by the glycosylation inhibitor, tunicamycin (Supplementary Figure 5B). Thus, ER stress may contribute to Bim induction, but this response was not strongly induced.

Glut1 deletion led to reduced B-ALL cell viability over time (Figure 5b). The majority of cells, however, remained viable even without Glut1. However, increased expression of Bim in Glut1-deficient cells suggested that sensitivity of the surviving cells to apoptosis was increased. Indeed, treatment of BCR-Abl B-ALL with a low dose of the tyrosine kinase inhibitor, Dasatinib, only mildly impacted control B-ALL cells, but B-ALL cell death was markedly increased in Glut1-deleted cells (Figure 5c). Cell death appeared to occur in part through apoptosis, as Annexin V+ early-stage apoptotic cells were detected (Figure 5d) and caspase activity increased (Supplementary Figure 6). Importantly, caspase inhibition with Q-VD partially protected cells from death (Figure 5d).

### Glut1 deletion suppresses B-ALL progression *in vivo*

**Figure 6:**
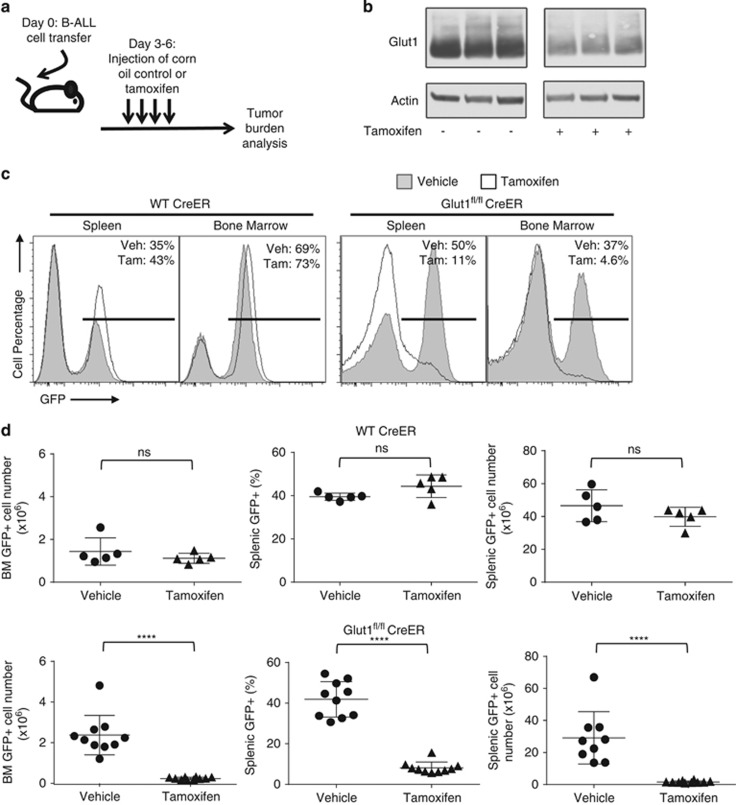
*In vivo* Glut1 deletion decreases leukemic tumor burden. (**a**) Schematic diagram showing tamoxifen treatment to induce Glut1 deletion *in vivo*. (**b**) Glut1^fl/fl^ CreER B-ALL were transferred and hosts treated with vehicle or tamoxifen on day 3, and purified B-ALL cells were analyzed by immunoblot from individual mice. (**c**) Flow cytometry from representative spleen and bone marrow on day 10 of recipient animals that received WT Cre-ER or Glut1^fl/fl^ Cre-ER and were treated with vehicle or tamoxifen. (**d**) Percentages and numbers of GFP+ transferred B-ALL cells on day 10 in individual mice treated as indicated with vehicle or tamoxifen. Means and S.D. from *n*=5 mice/group for WT CreER group and *n*=10 mice/group for Glut1^fl/fl^ CreER group are shown. ****P<0.0001. NS, not significant

Despite sharply curtailed growth and proliferation *in vitro*, how B-ALL progressed *in vivo* without Glut1 remained unclear. Presence of *in vivo* nutrients and stromal cell support may allow B-ALL cells to persist and proliferate even without Glut1 and with reduced glucose uptake. Control ^UbiCreERT2^ and Glut1^fl/fl^ ^UbiCreERT2^ B-ALL cells were, therefore, transferred into immunocompromised hosts that were treated with vehicle or tamoxifen to activate CreER^T2^, and *in vivo* and B-ALL growth was assessed with or without Glut1 expression (Figure 6a). B-ALL cells were monitored by IRES-driven GFP expression from the BCR-Abl expressing retroviral vector. Two days after cell transfer, recipients were treated with tamoxifen to delete Glut1 in transferred B-ALL cells. Animals were then analyzed for B-ALL cell number after an additional week. Glut1 was efficiently deleted *in vivo*, and Glut1 protein levels were sharply reduced in B-ALL cells purified from splenocytes of tamoxifen-treated recipient animals (Figure 6b). Both ^UbiCreERT2^ control and Glut1^fl/fl^ ^UbiCreERT2^ B-ALL cells were present in high levels in both spleen and bone marrow of vehicle-treated mice (Figures 6c and d). Importantly, B-ALL cells did not accumulate *in vivo* and disease did not rapidly progress after Glut1 deletion (Figure 6c) and numbers of Glut1-deficient B-ALL cells were significantly reduced in spleen and bone marrow (Figure 6d), relative to vehicle-treated or control B-ALL. These data indicate that Glut1 deletion suppresses B-ALL progression, and B-ALL cells are dependent on Glut1 even in physiological conditions.

### Pharmacological inhibition of glycolysis impairs proliferation and leads to Bim-dependent cell death to sensitize to targeted therapy

**Figure 7:**
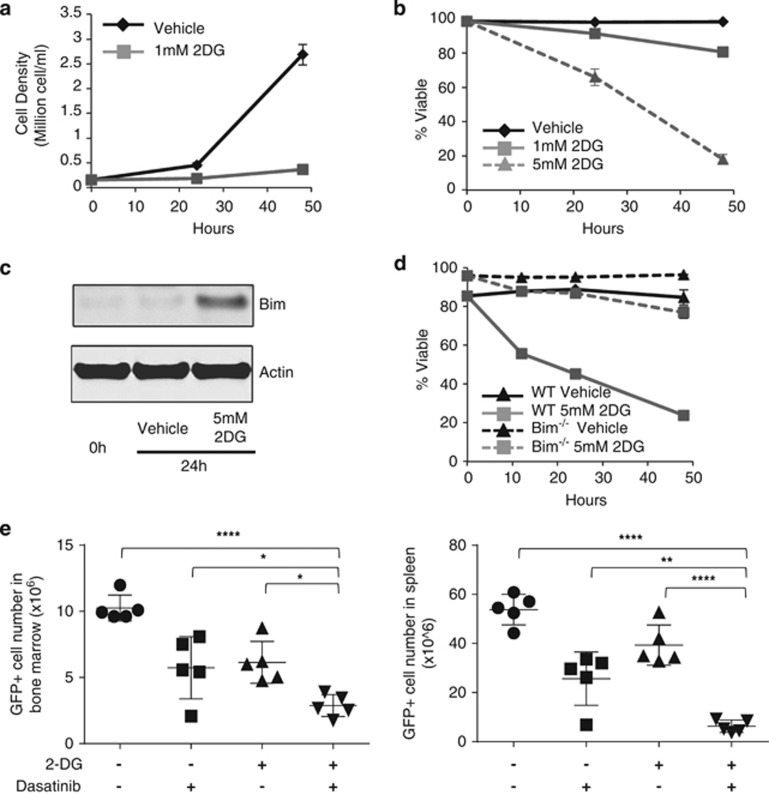
Pharmacological inhibition of glycolysis impairs B-ALL proliferation and sensitizes to apoptosis *in vitro* and *in vivo*. (**a**–**c**) WT B-ALL cells were cultured in low (1 mM) or high (5 mM) dose of 2-DG and (**a**) cell number was counted and (**b**) cell viability was analyzed by flow cytometry over time. (**c**) Cell lysates were analyzed by immunoblot. (**d**) WT or Bim^−/−^ B-ALL cells were treated with 5 mM 2-DG and cell viability was analyzed by flow cytometry over time. (**e**) WT B-ALL cells were adoptively transferred into host animals that were treated starting on day 2 with vehicle alone or with 500 mg/kg per day of 2-DG alone for 4 days or with additional 10 mg/kg per day of Dasatinib for 3 days. GFP+ B-ALL cell percentages and numbers were determined by flow cytometry. Means and S.D. are shown of (**a**, **b** and **d**) triplicate and (**e**) *n*=5 mice/group. ****P<0.0001, ***P<0.001, **P<0.005, *P<0.05

Given the dependence of B-ALL cells on Glut1, the cellular response to pharmacological disruption of glucose metabolism was next examined by treating BCR-Abl B-ALL with 2-DG. BCR-Abl+ murine B-ALL cells were dependent on continued glycolysis and a low dose of 2-DG prevented cell accumulation (Figure 7a). A higher dose of 2-DG to further inhibit glycolysis was required to induce B-ALL cell death, similar to the observation in human primary B-ALL cells (Figure 7b). This dose of 2-DG treatment induced Bim expression (Figure 7c), which was essential to induce apoptosis, as Bim^−/−^ B-ALL cells resisted cell death even with high doses of 2-DG (Figure 7d).

The potential of 2-DG to provide an adjuvant metabolic stress to specifically sensitize cancer cells to Dasatinib was next tested *in vivo*. Wild-type B-ALL cells were adoptively transferred into recipient animals and allowed 2 days to engraft. To provoke metabolic stress and prime B-ALL cells for targeted therapy, animals were treated with vehicle or 2-DG for 4 days alone, followed by three additional days with or without addition of Dasatinib. B-ALL cell percentages and numbers were then determined 11 days after transfer (Figure 7e). 2-DG and Dasatinib alone each reduced B-ALL cell burden. Importantly, combined treatment of 2-DG and Dasatinib together led to significantly further depletion of B-ALL cells. Likewise, 2-DG treatment also increased efficacy of Dasatinib to induce cell death in human BCR-Abl+ B-ALL cell lines (Supplementary Figure 7). Thus, partial non-cytotoxic pharmacologic inhibition of glucose metabolism slows tumor growth and provides an enhanced response to specifically targeted therapy.

## Discussion

Elevated rates of glucose uptake and glycolysis can have significant roles in cancer cell survival and progression by supporting cellular energetics and providing biosynthetic substrates.^25^ It is now important to establish limiting components of aerobic glycolysis and how cancer cells respond to metabolic inhibition. Here we show that B-ALL cells are highly dependent on glucose and that glucose uptake through Glut1 is essential for BCR-Abl B-ALL cells to maintain anabolic metabolism to support proliferation. Glut1-deficient B-ALL cells both proliferated at a lower rate and had moderate levels of apoptosis. Importantly, B-ALL cells required Glut1 to progress *in vivo*, demonstrating a clear role for this specific glucose transporter in B-ALL metabolism. Similar to Glut1 deletion, pharmacologic inhibition of aerobic glycolysis with 2-DG also suppressed proliferation and led to increased expression of Bim to sensitize to the tyrosine kinase inhibitor Dasatinib *in vivo*. Partial metabolic stress thus impaired cell proliferation and sensitized BCR-Abl-driven B-ALL cells to apoptosis with specifically targeted therapy *in vivo*.

Similar to the glycolytic form of DLBCL,^3^ we show here that both BCR-Abl+ and BCR-Abl− human B-ALL cell lines are highly glycolytic and metabolize glucose through aerobic glycolysis. Importantly, *in vitro*, B-ALL cells were sensitive to inhibition of glycolysis with reduced proliferation followed by apoptosis. *In vitro* culture conditions, however, do not faithfully mimic *in vivo* nutrient conditions. Presence of alternate nutrients, stromal cells and growth factors in a physiological setting may substantially modulate sensitivity of cells to therapy. Primary B-ALL cells with conditional deletion of Glut1 allows for a direct test of glucose metabolism contribution to B-ALL progression *in vivo*. Glut1 deletion did not fully prevent glucose uptake, but instead glucose transport was reduced to approximately half in Glut1-deficient cells. The remaining glucose uptake was likely mediated through other glucose transporters expressed by B-ALL cells.^26, 27, 28^ In particular, Gluts 3 and 6 may increase activity to support this glucose transport.^17^ Human B-ALL cells expressed additional transporters, including Glut4 and Glut5 (Supplementary Figure S1B). The array and expression levels of these different transporters, however, differed across individual cell lines to provide potentially different capacities to adapt and compensate for metabolic inhibition. Our data suggest, however, that Glut1 or Glut3 may have dominant roles.

Despite the incomplete inhibition of glucose uptake, Glut1 deletion led to striking shifts in metabolic pathways that demonstrate glucose transport as a limiting component of B-ALL cell metabolism. Glut1-deficient cells had significantly decreased flux through biosynthetic pathways, including the pentose phosphate pathway, nucleotide and phospho-lipid synthetic pathways, but had elevated lipid metabolism intermediates and oxidation rates. Surprisingly, the remaining glucose uptake and glycolytic flux in Glut1-deficient B-ALL was not redirected to oxidative metabolism and the TCA cycle for maximum ATP generation. Rather, even Glut1-deficient B-ALL cells remained dependent on non-glucose sources for TCA intermediates. It is possible that a BCR-Abl signal may suppress pyruvate oxidation even under nutrient-limiting conditions. As a consequence, B-ALL cell growth and proliferation were sharply curtailed by Glut1 deletion. Cells did not arrest in a specific cell cycle stage, but rather cell proliferation appeared slowed in each phase in a balanced fashion. It may be that a more severe form of metabolic inhibition is essential to induce cell cycle checkpoints and arrest, such as p21 induction that can be induced through AMPK activation of p53,^8,29^ but we observed B-ALL cells to instead undergo apoptosis in these conditions.

Similar to 2-DG treatment, Glut1 deletion also induced expression of pro-apoptotic protein Bim and decreased B-ALL viability partially through apoptosis, although non-apoptotic cell death may have occurred as well. Bim appears to be the primary Bcl-2 family protein mediating apoptotic events, as expression levels of other Bcl-2 family proteins were only modestly altered by Glut1 deletion (Supplementary Figure S5). Glut1 deletion did not fully eliminate all B-ALL cells, possibly because the level of metabolic stress from Glut1 deletion was insufficient to kill all the cells. Nevertheless, Glut1 deficiency sensitized the remaining B-ALL cells to cell death stimulus. A sub-lethal dose of BCR-Abl inhibitor, Dasatinib, rapidly induced apoptosis in Glut1-deleted cells. It is unclear what pathway was directly responsible for Bim induction. It has been reported that decreased protein glycosylation upon glucose deprivation or 2-DG can lead to endoplasmic reticulum stress,^11,30^ which may promote Bim induction through the transcription factor CHOP.^24,31^ However, Glut1 deficiency only imposed mild, if any, ER stress to B-ALL cells. The role of this mild ER stress on Bim induction remains unclear.

Interactions of B-ALL cells with stromal cells and the availability of alternate nutrients may support metabolic flexibility to allow cancer progression *in vivo* even with limited glucose uptake. *In vivo* deletion of Glut1 from B-ALL cells, however, markedly reduced leukemic tumor burden in recipient animals and prevented disease progression. The significant reduction of tumor burden may be due to the combinational effects of suppressed cell proliferation and impaired cell viability. These data demonstrate that glucose uptake and Glut1 are limiting components in the support of aerobic glycolysis in BCR-Abl+ B-ALL cells *in vivo*. Similarly, Glut1 deficiency suppressed breast cancer progression.^32^ Other glycolytic enzymes have also been shown to have key-limiting roles in cancer metabolism, including pyruvate kinase M2,^33^ phospho-fructokinase,^34^ and lactate dehydrogenase A.^35^ Our data support Glut1 as an additional potential restriction point in cancer metabolism.

These data collectively suggest Glut1 may provide a therapeutic target to reduce glucose uptake and treat cancers that use aerobic glycolysis. Although Glut1 is essential for glucose uptake in some tissues,^36,37^ incomplete inhibition of Glut1 may be feasible and provide benefits. Indeed, several groups have now described inhibitors of Glut1 with partial activity that can suppress tumor growth.^38, 39, 40, 41^ The HIV protease inhibitor, ritonavir, also shows non-selective partial inhibition of both Glut1 and Glut4 with low toxicity.^42^ Although it remains unclear to what extent Glut1 may provide a direct pharmacologic target, our data indicate that it is not essential to fully suppress glucose uptake to prevent cancer cell proliferation and disease progression.

Together, we show B-ALL is a highly glycolytic cancer dependent on Glut1 as a limiting component of glucose and anabolic metabolism. Glycolytic inhibition triggers a graded response of reduced cell proliferation followed by increased sensitivity to apoptosis both *in vitro* and *in vivo*. Impairment of glucose uptake through Glut1 deletion sufficiently suppressed biosynthetic reactions and shifted B-ALL metabolic state to catabolism. This metabolic reprogramming impeded B-ALL proliferation and prevented disease progression *in vivo*. The glucose dependence observed in B-ALL is likely also present in other types of cancer, and partial blockade of aerobic glycolysis may provide an adjuvant approach to augment the efficacy of targeted agents with minimal additional toxicity.

## Materials and Methods

### Human primary B-ALL and B-cell culture

Human B-ALL cell lines (BV-173, TOM-1, Nalm-16, Nalm-19, and KOPN-8 from DSMZ (Braunschweig, Germany); Sup-B15 from ATCC (Manassas, VA, USA)) were cultured in complete RPMI 1640 (Mediatech, Manassas, VA, USA) or complete IMDM (Gibco, Grand Island, NY, USA) with 10 or 20% fetal bovine serum (Gemini Bioproducts, West Sacramento, CA, USA). De-identified primary human B-ALL samples were cultured in Hybridoma SFM media (Gibco) supplemented with cytokines (refer to Supplementary Materials for list of cytokines). Human B cells from peripheral blood (Gulf Coast Regional Blood Center, Houston, TX, USA) were purified by negative selection (Stem Cell Technologies, Vancouver, BC, Canada) and cultured in the same media as B-ALL cells. Some samples were treated with 2-DG (Sigma-Aldrich, St. Louis, MO, USA) at indicated doses. Samples were monitored by flow cytometry using anti-human CD34, CD19, CD10 and CD20 (eBioscience, San Diego, CA, USA). Use of the human subject-derived samples was approved by the Duke University Institutional Review Board.

### Primary murine BCR-Abl+ B-ALL

Bone marrow cells from C57B6/J (Jackson Labs, Bar Harbor, ME, USA), Bim^−/−^ (Jackson Labs), Ubi-Cre-ER^T2^ transgenic (Jackson Labs), and Glut1^fl/fl^ mice crossed to Ubi-Cre-ER^T2^ mice were cultured in IL-7 (10 ng/ml) (eBioscience) in IMDM supplemented with 20% fetal bovine serum and infected with MSCV-BCR-Abl-IRES-GFP retrovirus (gift of D Fruman, UC Irvine)^7^ with polybrene (4 *μ*g/ml) (Millipore, Billerica, MA, USA). Infected cells were cultured in methylcellulose medium containing IL-7 (Stem Cell Technologies) and individual colonies were isolated on day 7 and transferred into complete IMDM media with 20% fetal bovine serum, but no IL-7. After 7 days, viable GFP+ colonies were selected for expansion. The Institutional Animal Care and Use Committee of Duke University approved all animal protocols.

### Immunoblot and flow cytometry

Cell lysates for immunoblots were prepared as described.^4,7,12^ Primary antibodies used were rabbit anti-Bim (BD Pharmingen, San Jose, CA, USA), rabbit anti-Glut1 (Abcam, Cambridge, MA, USA), rabbit anti-phospho-BCR (Cell Signaling, Danvers, MA, USA), rabbit anti-BCR (Cell Signaling), rabbit anti-phospho-CRKL (Cell signaling), rabbit anti-CRKL (Santa Cruz, Dallas, TX, USA), rabbit anti-Bcl-xL (Cell signaling), rabbit anti-Mcl-1 (Biolegend, San Diego, CA, USA), rabbit anti-Bax (Cell signaling), rabbit anti-Bak (Cell signaling), rabbit anti-Bid (mouse specific, Cell signaling), rabbit anti-PDI (Cell signaling), rabbit anti-IRE1*α* (Cell signaling), rabbit anti-Bip (Cell Signaling), mouse anti-CHOP (Cell signaling), mouse anti-actin (Sigma-Aldrich) and were detected with anti-rabbit horseradish peroxidase-labeled antibody (Promega, Madison, WI, USA) and fluorescent-labeled anti-mouse antibody (LiCor, Lincoln, NE, USA). Blots were visualized using Supersignal West Pico Chemiluminescent Substrate (Thermo Scientific, Waltham, MA, USA) or the Odyssey infrared imaging system (LiCor).

Antibodies for cytometry include anti-human CD34, CD19, CD10 and CD20 (eBioscience), annexin V (Invitrogen, Grand Island, NY, USA) and anti-BrDU (Invitrogen). Cell viability was measured by flow cytometry for propidium iodide (PI; Invitrogen) exclusion as described.^12,13^ Apoptotic cell populations were assessed by annexin V/PI staining. Cells were incubated with Alexa Fluor 488 annexin V (Invitrogen) for 15 min in dark and then 2 *μ*g/ml PI was added. Cell cycle profile and DNA content were measured in cells fixed in ethanol and stained with PI. BrDU incorporation was measured by culture of cells with 10 uM BrDU (Sigma-Aldrich) for 1.5 h followed by ethanol fixation, denaturation with 2 M HCl for 20 min and staining with anti-BrdU. Flow cytometry data were collected on MACSQuant (Miltenyi, Bergisch Gladbach, Germany) or FACScan (Becton Dickinson, San Jose, CA, USA) flow cytometers and analyzed using FlowJo software (Treestar, Ashland, OR, USA).

### Quantitative real-time-PCR

Total mRNA was extracted (RNeasy mini kit; Qiagen, Valencia, CA, USA) and 1 *μ*g of RNA was reverse transcribed (iScript; Bio-Rad, Hercules, CA, USA) to perform SYBR Green (Bio-Rad) semi-quantitative real-time-PCR for Glut1. Relative expression levels were calculated using the ΔCt/ΔCt method, with expression normalized to 18S or actin RNA. Primers for mouse glucose transporters and mouse actin: Glut1, 5'-AGCCCTGCTACAGTGTAT-3', 5'-AGGTCTCGGGTCACATC-3'; Glut3, 5'-TAAACCAGCTGGGCATCGTTGTTG-3', 5'-AATGATGGTTAAGCCAAGGAGCCC-3' Glut6, 5'-TTGGTGCTGTGAGGCT-3', 5'-TGGCACAAACTGGACGTA-3'; Glut8, 5'-ACATCTCGGAAATCGCCT-3', 5'-ACACAGCCCAGCACG-3'; Glut9, 5'-TGCTTCCTCGTCTTCGCCACAATA-3', 5'-CTCTTGGCAAATGCCTGGCTGATT-3'; actin, 5'-CCTTCCTTCTTGGGTATGGA-3', 5'-TGGTACCACCAGACAGCACT-3'. Primers for human glucose transporters and 18S: Glut1, 5'-CACTCCTGTTACTTACCTAA-3', 5'-CACTTACTTCTGTCTCACT-3'; Glut3, 5'-GACCCAGAGATGCTGTAATGGT-3', 5'-GGGGTGACCTTCTGTGTCCC-3'; Glut4, 5'-CTTCCAACAGATAGGCTCCG-3', 5'-CCCCAATGTTGTACCCAAAC-3'; Glut5, 5'-GCAACAGGATCAGAGCATGA-3', 5'-CCATACTGGAAGGATGACCC-3'; Glut6, 5'-GTCCATCTTCGACAGCACCG-3', 5'-GCAAACATGATGGCCGCTGA-3'; Glut7, 5'-CACCGTCTCCATGTTTCCTC-3', 5'-TGTTGTTGATCAGCAGGGTC-3'; Glut8, 5'-TCCTGGTTCGGGGCTGTC-3', 5'-GAGCACAGCAAGAGGCTCAG-3'; Glut9, 5'-GAGTATCGTGGGCATTCTGG-3', 5'-AGTTGGAGAGCCAGTTGACG-3'; 18S, 5'-GTAACCCGTTGAACCCCATT-3', 5'-CCATCCAATCGGTAGTAGCG-3'.

### Metabolomic profiling

Metabolomic analyses were performed as described before using la iquid chromatography Q Exactive Mass Spectrometer (LC-QE-MS) (Thermo Scientific).^22^ In non-targeted metabolomics analyses, Glut1^fl/fl^ CreER and WT CreER cells were treated with vehicle or 0.4 *μ*M 4-OHT for 96 h followed by an additional 48 h in complete IMDM media containing 1 mM sodium pyruvate. For 13C-glucose flux studies, cells were cultured as described above for 5 days culture, washed with PBS (Mediatech), and cultured in glucose-free RPMI without sodium pyruvate (Gibco) supplemented with 10% fetal bovine serum, glutamine (2 mM) and uniformly labeled 13C-glucose (10 mM; Cambridge Isotope Laboratories, Tewksbury, MA, USA) for 24 h. Metaboanalyst was used to range-scale data and provide PCA and KEGG pathway analysis of metabolites significantly changed (1.5-fold difference, *P*<0.05)(www.metaboanalyst.ca/).^43^

### Metabolic assays

Glucose uptake, lipid oxidation, and pentose phosphate pathway were measured as described.^4,44^ XF24 extracellular flux assays (Seahorse Biosciences, Billerica, MA, USA) were performed as described.^19^ Cells were initially incubated in XF basic media in the absence of glutamine or glucose. Glutamine, glucose, oligomycin and 2-DG were sequentially injected in the test to the following final concentrations: glutamine 2 mM, glucose 25 mM, oligomycin 1 *μ*M, 2-DG 20 mM. Measurements of ECAR and OCR were normalized to cell number.

### Caspase 3/7 activity assay

Caspase 3/7 activity was measured using caspase-Glo 3/7 assay kit (Promega). Equal number of live cells from different treatment conditions was used for the assay. Cells were incubated with substrates for 1 h at room temperature and a luminescent signal was measured on a luminescence plate reader.

### *In vivo* leukemia assays

B-ALL cells (2.5 × 10^5^) were injected via the tail vein into NOD/SCID/Gamma-immunodeficient mice (Duke Cancer Center, Durham, NC, USA). Two days after transfer, mice were treated with vehicle (corn oil; Sigma-Aldrich) or tamoxifen (120 mg/kg per day; Sigma-Aldrich) by i.p. injection for four consecutive days. GFP+B-ALL cells were observed in spleen and bone marrow by flow cytometry. In some cases, animals were treated with 2-DG (500 mg/kg per day; Sigma-Aldrich) or Dasatinib (10 mg/kg per day; Cell Signaling Technologies) through oral gavage, as indicated.

### Statistical analysis

Statistical analysis on data involving two groups was performed with unpaired two-tailed Student's *t*-test. Data involving more than two groups were analyzed using one-way ANOVA with Tukey's multiple comparison test. Adjusted *P*-values are shown. Statistical analysis was performed using Prism (Graphpad Software, La Jolla, CA, USA).

## Abbreviations

B-ALL: B-cell acute lymphoblastic leukemia
2-DG: 2-deoxyglucose
DLBCL: diffuse large B cell lymphoma
ECAR: extracellular acidification rate
OCR: oxygen consumption rate
4-OHT: 4-hydroxytamoxifen
BrDU: BromodeoxyUridine
