# Supplementary material for: Glucose transporter 1-mediated glucose uptake is limiting for B-cell acute lymphoblastic leukemia anabolic metabolism and resistance to apoptosis
Source: Cell Death Dis. 2014 Oct 16;5(10):e1470–. doi: 10.1038/cddis.2014.431 (PMC4237255; doi:10.1038/cddis.2014.431)
Supplement: Supplementary Figure Legends [file cddis2014431x1.pdf]

Supplemental Figure Legends

Figure S1. Related to Figure 1. Human B-ALL cell lines express multiple glucose transporters. Glutamine or glucose do not significantly increase oxygen consumption rate in B-ALL cell lines or human B cells.

A. Oxygen consumption rates (OCR) in human B-ALL cell lines and normal B cells were measured at baseline (white bar, no metabolic fuel), followed by sequential injection of 2mM glutamine (grey bar) and 25mM glucose (black bar).
B. Expression of glucose transporters in human B-ALL cell lines was measured by qrtPCR. Shown are expressions of detected transporters relative to expression of 18S as internal control.
Means and standard deviations are shown for triplicate samples.

Figure S2. Related to Figure 1. Real-time PCR (RT-PCR) measuring *in vitro* Glut1 deletion efficiency. mRNA was harvested 96h after vehicle or 4-OHT treatment in Glut1$^{fl/fl}$ CreER or WT CreER cells. Values were normalized to beta-actin internal control. In each cell type, fold changes were calculated relative to vehicle control. Means and standard deviations from three independent experiments are shown. ****p<0.0001.

Figure S3. Related to Figure 2. Intracellular amino acid levels do not show significant changes following Glut1 deletion but intermediates related to nucleotides production show significant decreases. Levels of amino acids or their downstream intermediates related to nucleotide production were measured

by LC/MS and shown as normalized to vehicle control samples. Means and standard deviations are shown for triplicate samples. *$p<0.05$, ***$p<0.001$, ****$p<0.0001$.

Figure S4. Related to Figure 3. Glut1 deletion suppresses glucose contribution to anabolic metabolism. Glucose contribution to metabolites in glycolysis, TCA cycle and pentose phosphate pathway is shown. Left graphs show total quantity of indicated metabolites and right graphs show relative distribution of ^{13}C carbons in each metabolite. *$p<0.05$, **$p<0.01$, ***$p<0.001$, ****$p<0.0001$.

Figure S5. Related to Figure 5. Glut1 deletion imposes little ER stress on B-ALL cells and only has modest effects on Bcl-2 family proteins except Bim. WT Cre-ER and Glut1$^{fl/fl}$ Cre-ER B-ALL cells were treated with vehicle or 4-OHT for 96 hours followed by 48 hours of culture without 4-OHT. (A) Bcl-2 family protein expression and (B) ER stress markers were examined by immunoblot on day six.

Figure S6. Related to Figure 5. Glut1 deletion induces caspase activity that can be inhibited by Q-vd-oph. (A) WT Cre-ER and Glut1$^{fl/fl}$ Cre-ER B-ALL cells treated with vehicle or 4-OHT for 96 hours. In some cultures, pan caspase inhibitor 10μM Q-vd-oph was added for the last 24 hours as indicated. Caspase 3/7 activity was measured and values were normalized to vehicle control. (B)

After culturing with vehicle or 4-OHT for 96 hours, cells were washed and then cultured alone or with 50nM Dasatinib for an additional 48 hours. 10μM Q-vd-oph was added in some cultures as indicated. Caspase 3/7 activity was measured after 48 hours. Values were normalized to vehicle control without Dasatinib treatment. Means and standard deviations are shown for triplicate samples from representative experiments repeated three times. *$p<0.05$, **$p<0.01$, ***$p<0.001$, ****$p<0.0001$.

Figure S7. Related to Figure 7. 2-DG increases efficacy of Dasatinib to induce cell death in human B-ALL. Three human BCR-Abl+ B-ALL cell lines were cultured in the presence of 1mM 2-DG, 25nM Dasatinib or combination of the two drugs for 48 hours. Apoptosis was assessed by annexin V/PI staining (Grey bar, annexin V+/PI- cell percentage; Black bar, annexin V+/PI+ cell percentage). Means and standard deviations are shown for triplicate samples from representative experiments repeated three times. *$p<0.05$, **$p<0.01$, ***$p<0.001$, ****$p<0.0001$.
