# Supplementary material for: Glucose transporter 1-mediated glucose uptake is limiting for B-cell acute lymphoblastic leukemia anabolic metabolism and resistance to apoptosis
Source: Cell Death Dis. 2014 Oct 16;5(10):e1470–. doi: 10.1038/cddis.2014.431 (PMC4237255; doi:10.1038/cddis.2014.431)

Supplemental Fig 1

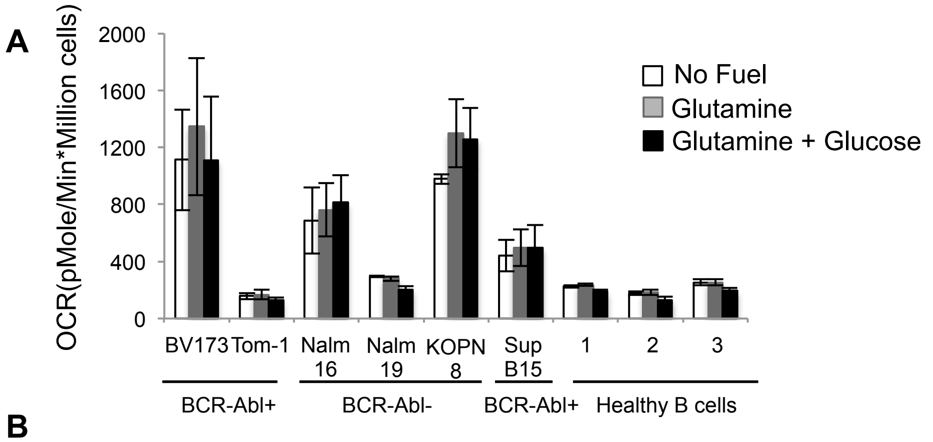

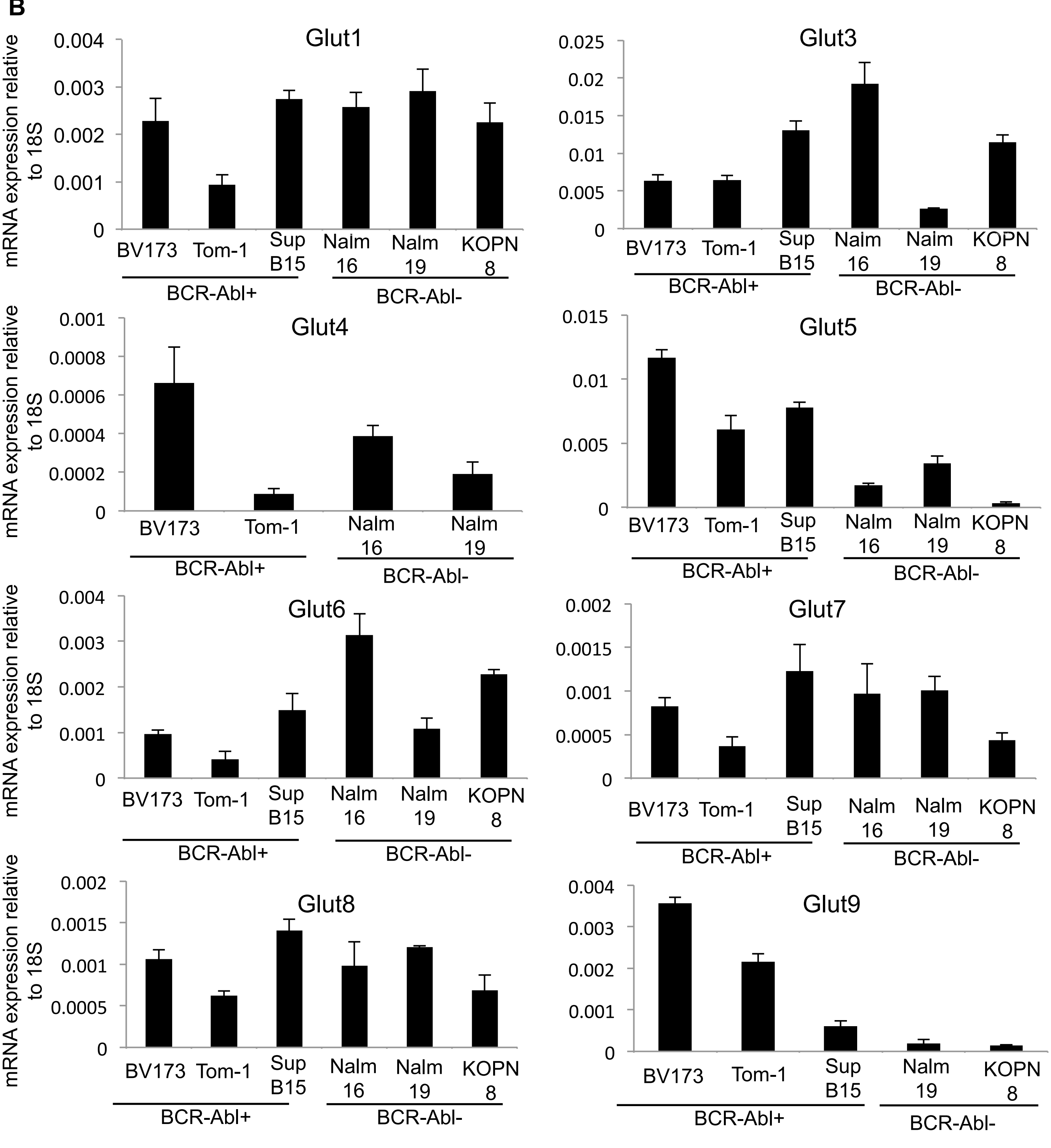

Supplemental Fig 2

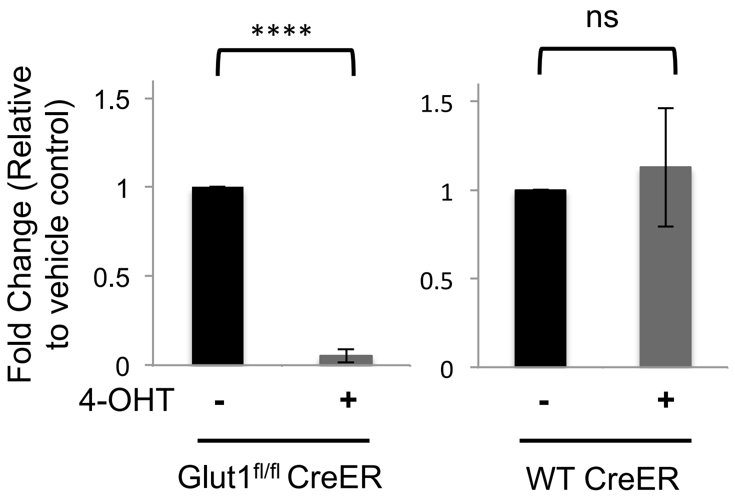

Supplemental Fig 3

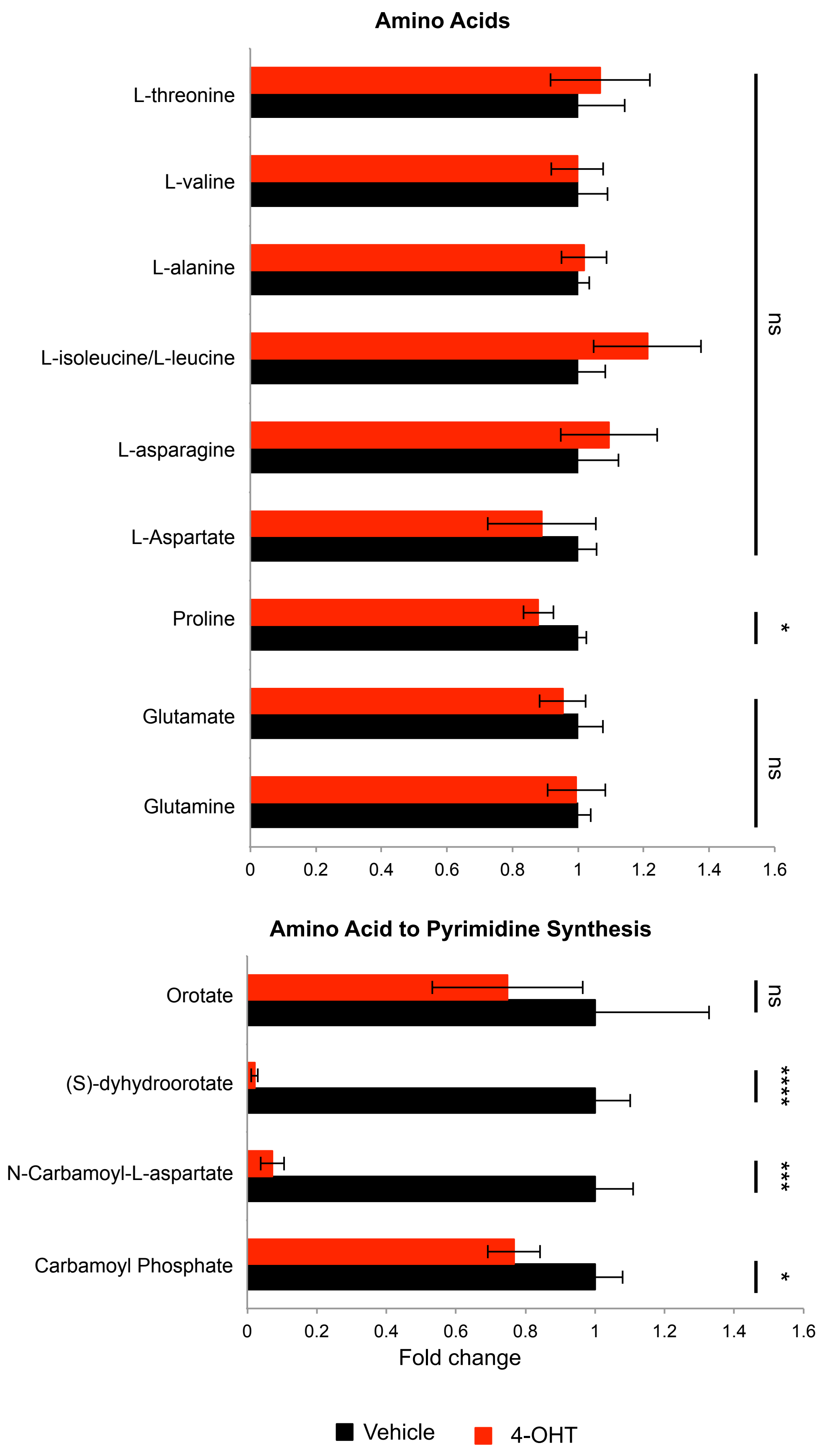

Supplemental Fig 4

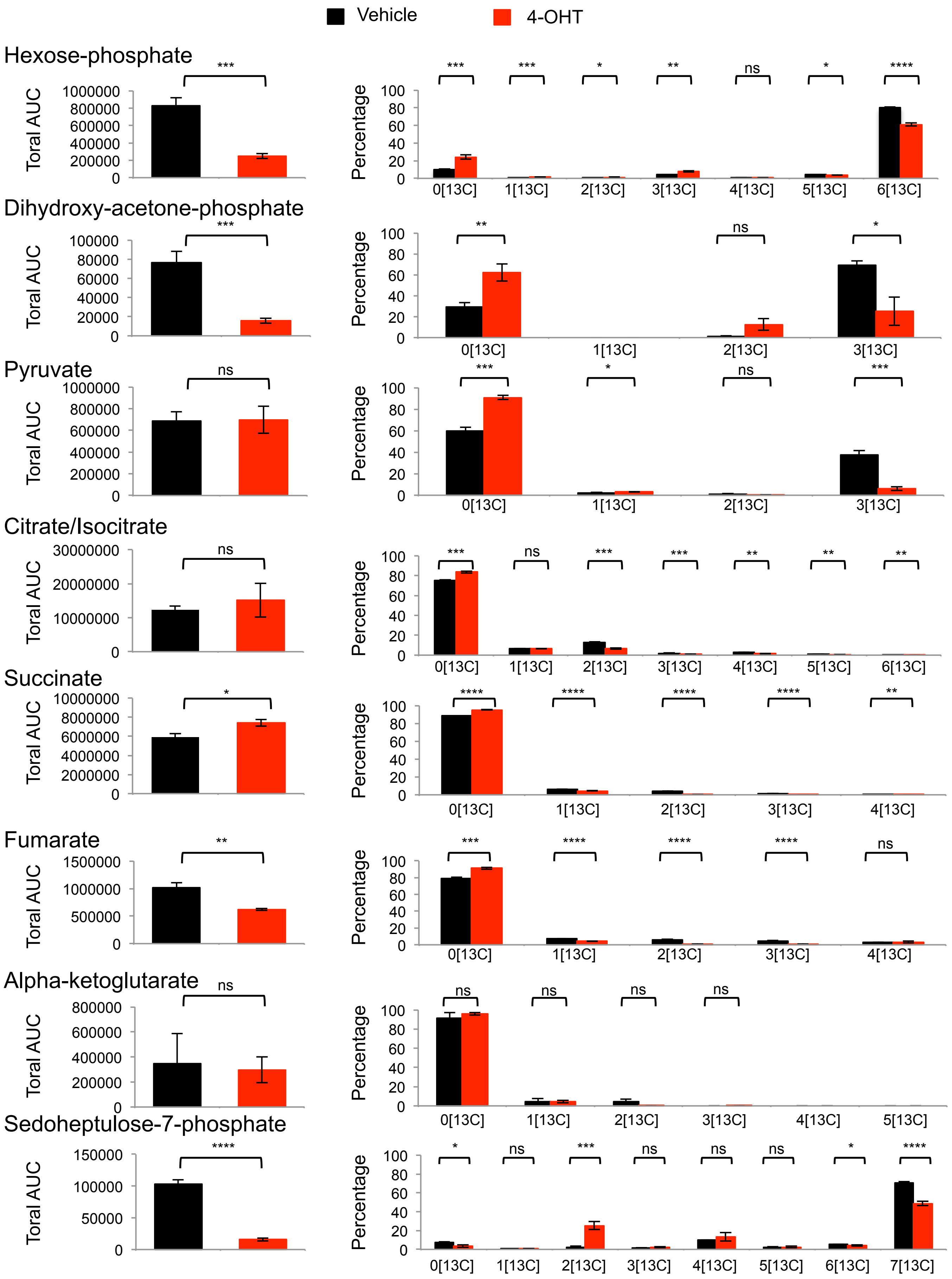

Supplemental Fig 5

A

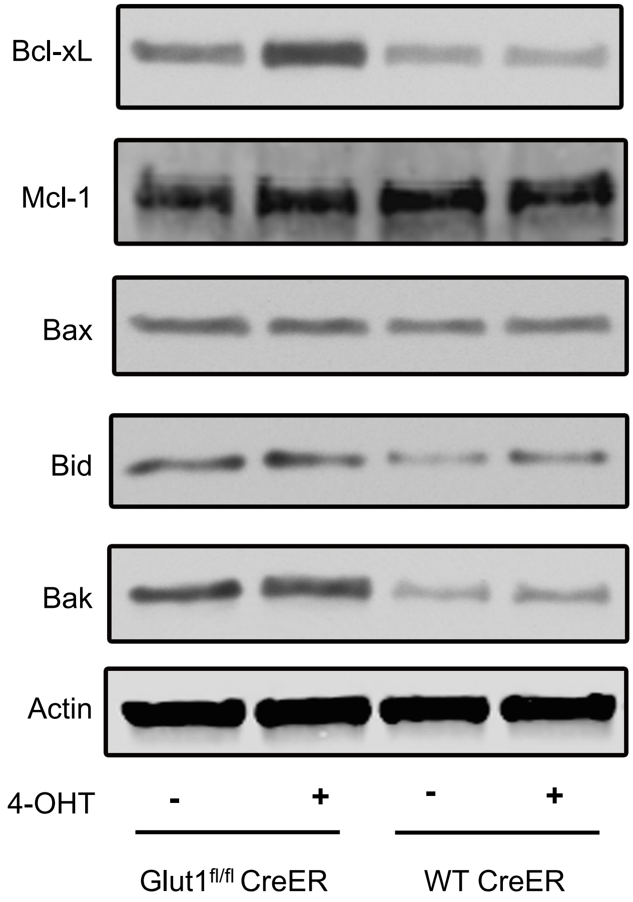

B

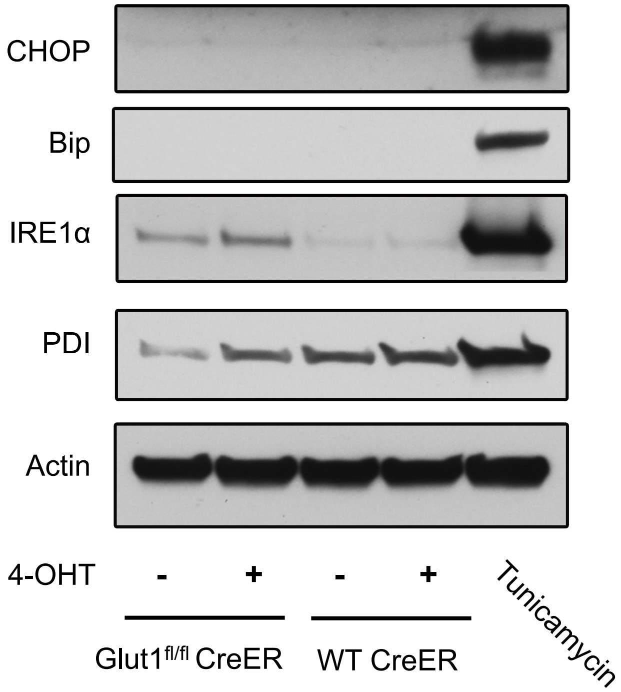

A

Post Glut Deletion and prior to Dasatinib addition

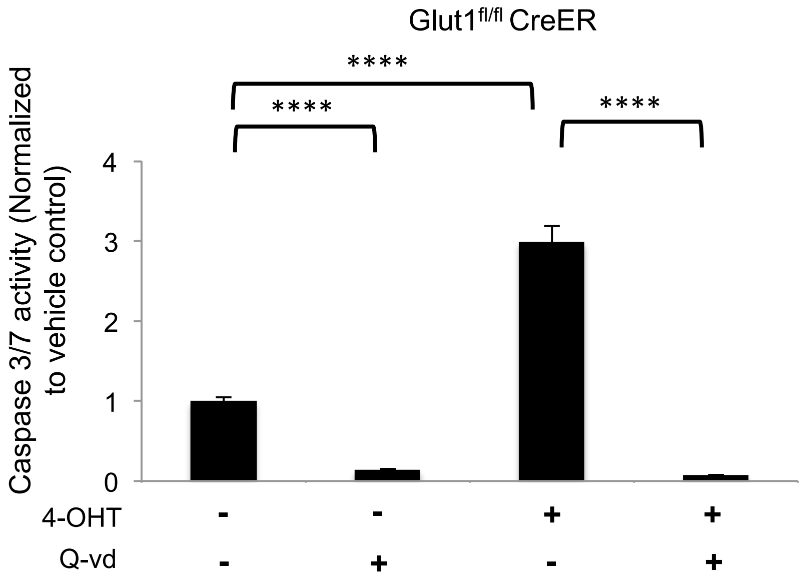

48 hours post Dasatinib addition

B

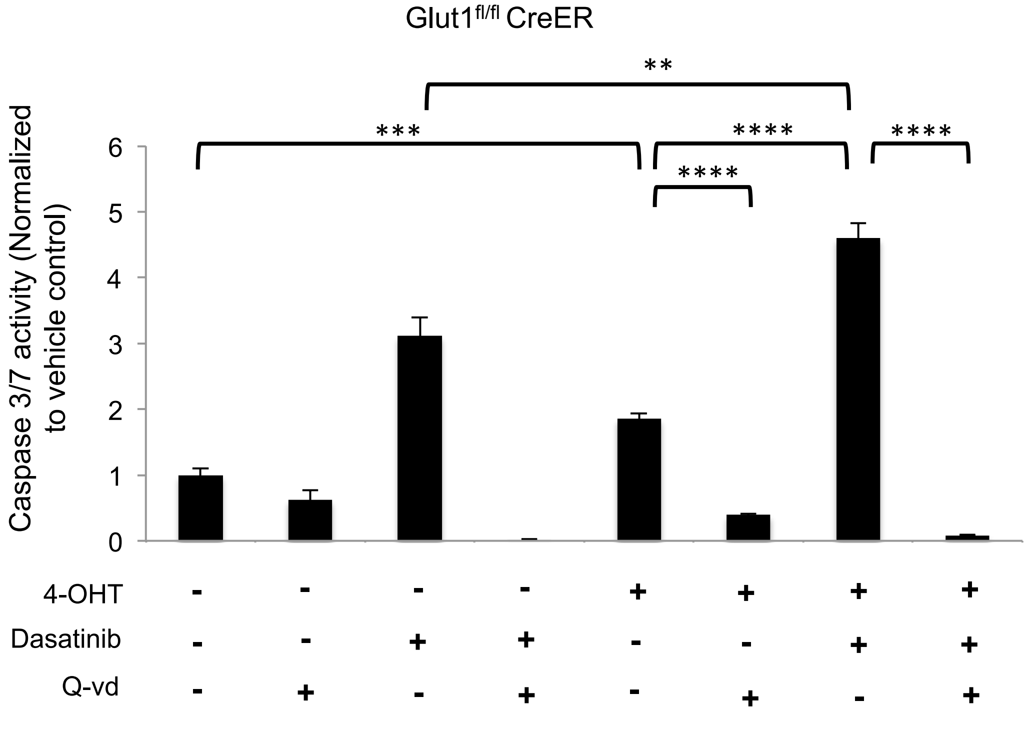

Supplemental Fig 7

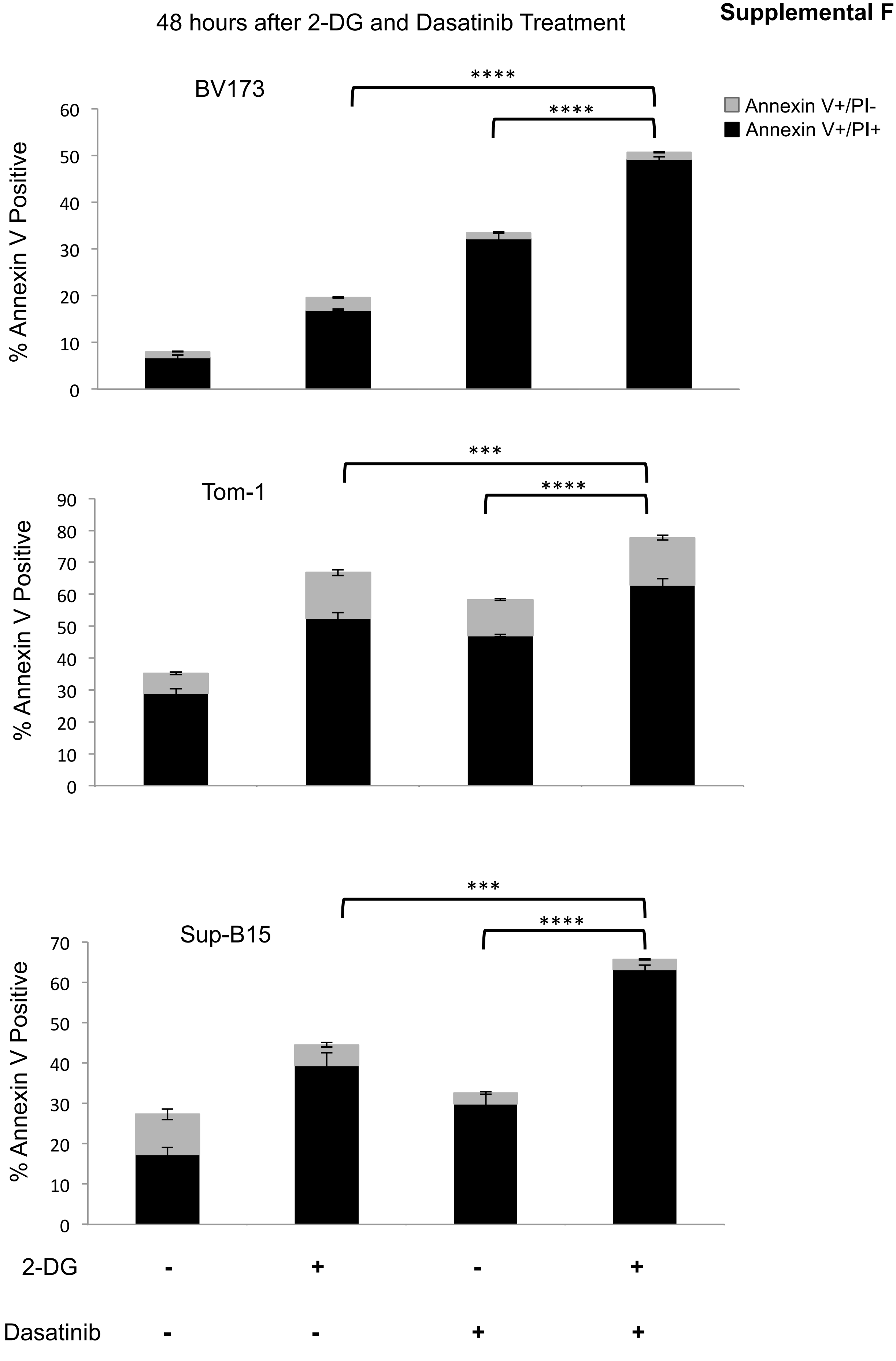

Supplement: Supplementary Figures [file cddis2014431x2.pdf]
