# Supplementary material for: Glucose transporter 1-mediated glucose uptake is limiting for B-cell acute lymphoblastic leukemia anabolic metabolism and resistance to apoptosis
Source: Cell Death Dis. 2014 Oct 16;5(10):e1470–. doi: 10.1038/cddis.2014.431 (PMC4237255; doi:10.1038/cddis.2014.431)
Supplement: Supplementary Table S1 [file cddis2014431x3.pdf]

Table 1. Non-targeted metabolomics results from Control and Glut1fl/fl cells +/- Tamoxifem

Cells	WT	WT	WT	WT	WT	WT	Glut1fl/fl	Glut1fl/fl	Glut1fl/fl	Glut1fl/fl	Glut1fl/fl	Glut1fl/fl
Treatment	Vehicle	Vehicle	Vehicle	4-OHT	4-OHT	4-OHT	Vehicle	Vehicle	Vehicle	4-OHT	4-OHT	4-OHT
2-aminoacrylic acid	1752176.05	1637787.61	1588959.02	1536981.64	1564537.88	1667519.34	1278792.61	1302061.22	1142526.5	914858.03	1065699.29	1024695.73
D-serine	3954285.93	4120787.33	4232368.62	3792468.93	3960453.54	4308861.32	3374302.33	3947620.43	3102925.11	2633467.23	3456772.85	3286207.15
N-methylethanolaminium	1023078.23	904059.146	882015.926	898543.584	843548.773	1102985.3	979422.541	794229.795	896944.221	2568117.16	2000786.86	2099411.49
cytosine	490372.589	483774.36	617539.089	522987.203	558131.254	525393.915	251298.374	636813.634	289197.708	226545.823	666306.849	679220.288
creatinine	82798316.6	82280975	85845532	78721458.5	80811626.4	89255902.6	50201677.9	68002627.5	56673512.2	51970068.1	76512030.9	84089773.9
creatine	61143440.4	59295928.1	67290085.8	62313336	65567433.7	59145500	44914959.7	43478081.8	44408702.3	46277754.4	40923322.9	57566030.6
Phosphodimethylethanola	4254699.14	3133195.84	2854649.22	3552817.96	2871026.6	3886484.25	2238237.23	1537322.98	2201762.21	2374158.42	1378322.25	1843382.97
56-dihydrothymine	3293449.67	3157273.37	3565942.87	3150636.97	3265859.01	2943211.97	1918330.24	1768206.15	2026538.38	1857849.43	1773656.01	2388357.01
2-deoxy-D-ribose	7869.52575	14143.3478	12105.509	10176.0654	10702.4514	5776.5596	6759.87075	7395.35105	4303.6851	4082.20575	8871.28515	12932.704
adenine	308618.795	374711.822	369763.057	317325.902	385669.026	357773.028	247588.549	318117.028	332568.068	295297.769	347042.547	352467.033
Hypoxanthine	101798457	90676366	83782879.6	76530790.2	74819501.7	110464057	47236781.7	56988317	44782739.2	58036562.2	82266181.2	89034228.4
2-keto-4-methylthiobutyra	24539.8502	38308.3932	18513.9281	31298.8543	23248.1837	22324.1602	40193.738	22750.5379	29991.1945	110737.418	27472.5009	14191.8619
L-cysteinylglycine	377549.121	157706.321	217748.831	153067.045	190573.465	202830.037	93676.3352	364413.143	144973.906	1233.5219	42170.6825	313373.475
choline phosphate(1-)	11914292.7	9088049.82	7076068.12	11407495.5	8225776.01	9986886.17	26904947	16288734.7	21474429.6	19412701.2	9140666.6	7478238.04
Nicotinamide	14923816.5	15094953.5	17348918	15445477.7	17082635	16614845.1	9236166.43	12781174.6	10427969.1	9205927.54	12475724.2	14752544.1
N-acetylputrescinium	1646138.36	2409678.92	2970450.98	1725179.79	2469102.2	2871544.81	1189647.29	1737454.62	2377074	1957450	1802989.11	2497758.49
Urocanate	124235.831	52050.5002	91213.9292	58673.611	97737.2269	102224.191	57159.6338	47579.1375	39097.8389	88060.4464	41838.9942	62851.8571
3-Methylimidazoleacetic a	171139.677	150700.415	188387.461	144906.967	158149.43	155862.112	114311.329	132679.308	137905.083	101208.3	120383.741	146344.413
4-Imidazolone-5-propanoa	66521.5559	50763.1831	58961.5665	49939.4037	60716.0315	47214.3977	40318.2775	33818.8269	41613.2547	34857.6926	38575.1488	37456.716
D-Alanyl-D-alanine	164345.669	159815.398	178903.081	136691.471	169744.156	162771.38	79424.5351	99742.2974	85858.458	60318.8454	78362.5923	113056.403
N-acetyl-L-asparagine	70280.0427	63649.8968	69049.5914	47793.0148	58751.1606	67605.9525	23225.2569	36884.8721	24002.6083	30484.3875	26974.924	48138.3653
citr_L[e]	1372323.84	1323996.7	1601388.04	1419733.9	1452687.84	1436513.93	833023.488	927226.12	865076.717	873284.056	1068906.74	1152957.41
D-Glucosamine	545732.379	446546.751	604457.72	568382.306	566673.116	516518.639	493895.259	371147.872	262670.43	430922.63	455571.159	578375.432
benzoate	390513.078	357320.897	417725.307	382987.638	413453.995	417722.519	304298.564	331664.72	317432.846	327602.933	335101.884	384358.875
4-trimethylammoniobutan	38288.9712	36051.0056	35806.8832	35765.8284	36656.2428	31948.8323	29149.1929	30948.6361	29224.3939	32451.0015	31188.48	32118.7128
1-methylnicotinamide	10427.4638	14248.9836	54565.2258	11702.4007	14677.8643	23128.6637	5062.06405	5544.02445	9337.07865	2582.16195	5001.4618	7841.30175
anthranilate	133218.262	149627.875	108139.982	109242.916	127596.542	149740.07	98045.0963	104454.138	97238.8534	75123.1288	78596.1763	47623.302
3-methylcrotonoylglycine	106486.088	113275.828	124361.088	102285.389	116429.975	131251.038	83524.6276	93947.1651	99120.5862	130374.905	109809.431	113927.776
(R)-carnitine	63445576.8	56966927.2	62417616.5	58088631.2	57926076.3	57039486.4	33120435.1	23719273.3	35582686	28313086.5	19409801.1	32594995.2
N(2)-acetyl-L-ornithine	960048.794	974113.906	1089708.83	910930.52	1014269.78	953008.179	598155.278	717605.675	620216.849	686740.781	721746.134	900129.402
2-phenylethanaminium	124148.125	146658.47	90412.706	104480.933	128371.796	133798.683	198813.659	169419.299	185503.305	1030288.66	249573.636	116796.253
tyraminium	125630.476	184524.934	143093.944	140216.436	150759.76	188975.85	283895.541	137878.258	307765.511	1155233.74	261896.019	154839.174
dopaminochrome	8178843.54	7356925.22	9578546.77	9620384.69	8773909.09	8554717.82	6715277.24	7560164.6	6056007.11	6110581.08	8107248.53	6396614.33
Pyridoxal	7654237.44	6947695.13	9191150.87	9147813.3	8472242.96	8154247.85	6362919.17	7265722.15	5800767.74	5811086.14	7761098.79	6331647.26
N-acetyl-D-glucosamine	73728.3363	74139.8589	79328.9478	73791.4019	75162.9624	88762.3608	35429.7143	47525.6087	39835.6591	40945.4575	67654.869	78512.7705
sn-Glycero-3-phosphochol	11468210.2	9578682.65	9465998.41	17135219.7	16236576.3	18801150.5	12861858.7	8963534.22	14048455.8	32784049.2	19505801.9	28295790
5-amino-1-(5-phospho-D-r	7331.1897	6938.8599	9085.26695	17425.0444	15089.0812	14769.9005	10632.7464	9536.9112	13510.0637	38912.5791	11789.9011	37133.5542
coumarin	180811.704	174243.917	180462.719	171640.923	192832.486	195451.117	139248.385	129532.532	132033.39	140611.16	166202.561	174633.267
N6N6N6-Trimethyl-L-lysine	9799960.43	9369721.33	10772743.9	9411689.87	9837540.86	9611498.74	5855009.04	6278716.76	5800047.22	6900629.26	7406503.33	10059008.3
L-alanyl-L-leucine	228775.802	247668.023	310791.477	266499.811	270869.322	244512.142	145714.392	165642.08	159667.221	182624.54	157154.124	237129.001
O-acetylcarnitine	26903220.9	24729146.5	21350548.3	20376562.3	18485486.4	21414828.5	35056384	28740607	30345712.4	24711751.9	23541272.6	20064673.5
3-Hydroxy-N6N6N6-trimet	339147.018	314285.762	308780.537	316541.438	313929.61	305212.346	206675.9	200031.984	184993.706	245549.193	238307.609	295413.591
2-deoxycytidine	67499.0294	67796.5371	78926.5261	67910.3403	73950.3306	63304.877	39751.011	116085.113	43908.4378	26281.9152	102319.279	81958.3859
C9H13N5O3(4)	33623146.3	34065734.5	35244472.4	34894233.7	36357772	33306909.9	31323244.9	33616407.4	31796125.5	32311457.7	33306288.2	36661523.8
naphthalene	13904.6465	14498.7615	9580.8793	14456.0235	11005.1278	16432.5709	24134.9605	13306.4898	17011.7401	69732.8177	21363.3993	17065.9931
perillyl aldehyde	348266.274	686749.688	260639.362	435695.814	429669.783	743782.002	1348737.81	435602.561	1296717.6	3612385.01	1430499.57	535819.905
serotonin(1+)	844027.209	830147.699	1013684.72	1247330.13	1459390.34	1270154.64	838302.236	1207087.69	1064934.13	970345.79	1390075.13	1725556.31
5-Hydroxyindoleacetate	16119.7171	13785.7323	18011.7476	11904.4414	23016.5938	16275.1811	7471.01225	14488.5497	9128.78495	9032.45135	18074.8457	16493.0854
N-methyl-467-trihydroxy-1	5880.71225	7349.0724	3426.2458	6841.0023	3315.95495	3147.80015	6756.8841	6913.55145	2072.4024	9401.60845	2482.79435	1000
propionyl-carnitine	3622105.74	3381418.46	3302218.78	3086054.95	3126783.83	3373943.52	4685563.29	4956817.66	4423601.09	4677702.7	5340836.85	4975786.19
2-deoxyguanosine/adenos	1672728.59	4897613.03	1187236.96	381413.938	127773.032	318963.343	311445.948	3351876.36	143812.301	716253.924	19267690.6	1384453.65
xanthosine	14189.8847	7888.0414	23503.6373	7696.1758	17983.6392	21202.8017	2959.41895	5759.5735	1000	1000	5071.1435	2556.14985
glutathionate(1-)	25982854.8	12089473.1	14746122.7	10958387.4	14371437.4	14617830.7	7020705.46	24859426.5	9759213.19	704878.272	3604909.04	23514362.8
beta-carboline	1000	1000	1000	1000	1000	1000	1000	1000	1000	1000	1000	1000
5-methoxytryptophol	24679.3479	46318.2448	44566.021	33393.6153	53239.0562	41782.9156	11038.4543	31491.6371	18463.764	26506.3689	32212.4154	41619.8985
N1N8-diacetylspermidine	9291.88985	11937.3185	15180.1947	13872.7116	13832.326	18274.4672	3263.1815	1000	1639.016	4802.74225	1000	3853.186
butyryl carnitine	31534731.9	32138281.1	28944012.1	26077653.8	25906378.7	28231110.9	22085362.9	23413246.7	21426136.7	14043502.3	16809698.9	15538818.5
lysyl-proline	4049.78935	3144.2272	1000	1629.9286	1000	1000	503505.282	395219.808	469541.925	393786.544	289514.551	346623.97
3-hydroxy butyryl carnitine	466088.535	418254.653	339669.662	335957.225	328962.025	361093.232	420804.508	367396.596	378369.391	326434.879	258197.106	216259.518
N-Ribosylnicotinamide	128940.04	128279.191	113549.848	105916.464	101247.381	93296.2005	134053.861	107042.727	132892.468	185205.092	108135.849	107247.607
succinyl carnitine	47511.8799	38770.9016	65908.3469	52859.2387	51963.2697	41128.2579	19860.3445	16355.7752	11184.5699	22092.6617	17139.2136	26639.6763
35-dihydroxy-34-dihydro-1	8330992.44	10240113.1	6047467.57	5637550.78	10374236.1	9974923.5	2722642.19	8752313.53	4764863.36	1118444.27	6947471.8	13141473.8
5-Methylthioadenosine	381684.339	427211.878	491135.622	288290.03	340254.739	486673.111	168518.522	199626.104	216794.158	121130.637	158843.414	249922.579
N-acetylneuraminate	1415280.51	1083763.04	1483823.84	2283948.48	1122192.46	848575.397	1017721.7	447366.585	303589.25	362118.022	473353.735	427593.061
NeuNGc	9446.90125	14362.2986	15070.3226	12138.6317	16127.0364	18098.1893	10861.0761	7397.7307	11717.1079	8711.8988	5199.661	5465.4892
tiglyl carnitine	364647.812	343147.004	324181.255	318736.365	322843.181	363191.871	315771.897	367363.361	314246.549	343614.776	341235.316	326089.006
3-hydroxy-isovaleryl carnit	184272.581	200978.478	113556.891	165313.307	131215.479	158788.513	228745.129	193593.575	208529.81	335894.457	187309.226	112960.782
thiamine(1+)	32072453	31194724	36390998.3	35087547.8	35555467.9	36171922.7	21890318.2	23457200	23873677.5	25092042.1	28009073.5	35990126.9
glutaryl carnitine	79756.5447	85749.6182	102645.949	93604.5327	104117.702	94426.3127	56447.3871	72291.2524	53985.8484	77075.7335	56071.9282	79208.162
S-(2-Methylpropanoyl)-dih	107249.475	55228.5566	106066.781	231813.878	66871.1175	27780.4873	23494.4416	9120.63975	3605.69655	8321.60455	3033.92825	5167.9311
octenoyl carnitine	36920.7021	39787.6366	29421.4412	30991.9169	25545.2403	33297.2968	54943.5579	47929.9133	47799.5402	84314.5525	65987.7115	47817.3882
octanoyl carnitine/L-Octan	244436.109	226658.207	194223.99	149076.168	166751.576	238308.502	197316.285	179680.497	176778.904	226672.164	202720.078	192275.94
12S-HHT	123560.986	226786.522	89450.5065	144126.03	151142.172	252487.777	521150.166	150580.725	501199.558	1554331.31	568650.16	182218.25

decenoyl carnitine	14732.5191	13578.0736	17047.2095	13752.3152	12959.6267	14747.8324	21362.8945	24367.9537	22934.9392	35647.4406	35840.5628	31452.3541
decanoyl carnitine	66263.2241	59262.2479	56592.698	48776.8133	52374.9508	67341.8455	55087.3696	61693.5712	61606.9401	66852.4514	63661.7024	65373.8101
3(S)10(R)-OH-octadeca-6-t	128907.41	237381.434	90974.6404	150601.134	158867.165	274474.532	559901.556	160944.221	564058.591	1711683.84	610706.219	190146.485
UDP(3-)	450881.293	426177.772	279726.779	393063.813	416249.721	462398.683	576726.667	571362.661	499867.751	323211.418	404140.033	152514.729
CMP-2-aminoethylphosph	1000	1000	1000	1000	1000	1040.4283	2301.658	2184.7182	1000	58557.5279	50181.6645	46768.241
CDP-ethanolamine(1-)	44657.0098	51304.0704	33593.0694	52975.0873	40365.3035	51603.3961	67373.6521	69236.4335	83849.816	312349.446	305309.59	285634.985
CMP-N-trimethyl-2-amino	25575.3591	26818.4681	25213.5759	21367.8727	29480.6571	36061.1643	26651.8411	24994.3058	20644.759	106831.647	104165.726	101050.398
CDPcholine	684512.649	556766.225	446219.533	434856.788	433937.59	652952.614	429074.278	392163.813	389752.918	747143.333	667708.906	770033.029
(2S)-2-azaniumyl-4-{[(1R)-2	5761480.39	7402519.18	5994050.3	6180821.05	5918246.29	7063326.96	7915626.63	5086926.47	7039059.44	10355707.4	11678691.5	8329207.23
CMP-N-acetyl-beta-neuran	585737.357	754703.271	592968.515	614715.441	585565.194	713766.066	738386.865	492416.817	689452.642	996856.392	1085060.81	779623.414
tetradecenoyl carnitine	33180.4207	23111.1441	32334.0578	31888.9452	31480.0023	53772.3711	26459.368	39307.9066	26747.288	54936.2178	59245.5931	84363.8304
NAD(1-)	2082049.48	2091897.49	1836671.39	1912878.21	1913282.94	1875007.58	1674084.32	1261157.59	1470095.36	1146885	962002.277	1021407.35
NADP+(3-)	24656.6272	39299.3849	5673.46845	25140.3493	30094.115	33172.0937	54494.32	129751.597	40739.4896	33247.0301	102305.288	11402.6507
pendtadenoyl carnitine	11973.4485	14672.1944	29885.6303	9122.3442	13394.8525	26768.6859	110922.66	14546.0864	49381.0996	112248.182	42324.19	16374.3944
L-palmitoylcarnitine	669022.379	453702.068	478625.513	619719.394	640811.53	587955.093	659466.778	913652.733	675475.259	1253163.44	1437055.81	1958513.98
3-hydroxyhexadecenoylca	10799.866	8241.9894	9566.80075	12303.9258	9479.9606	22649.0434	23656.09	30236.1148	23945.7701	22019.8372	32207.9677	37248.0454
3-hydroxyhexadecanoylca	16807.3427	11200.4607	11618.667	15670.5514	15585.1036	19316.3687	44389.201	50492.554	40342.5784	52622.207	51311.2203	60301.0696
heptadecanoyl carnitine	45899.4573	40149.2876	37943.0598	45490.6699	68316.3722	49548.465	67400.3584	88020.2585	67091.6946	131881.31	121422.894	152570.675
stearoylcarnitine	159316.319	130514.044	127782.375	145084.526	356263.546	178378.454	246679.892	289077.323	206559.499	350876.391	416136.819	523034.586
25-hydroxyvitamin D3-26-2	7273.909	1000	3905.5475	6594.0035	5050.5502	5791.379	1000	4422.5744	5082.53065	12877.0824	3469.51905	3194.8179
24-oxo-1alpha-23-25-trihy	8211.31225	12656.3471	4053.9141	8557.6675	6577.01935	11342.6689	25628.1611	6533.4296	24493.2701	128415.64	22082.0083	6923.9307
13-carboxy-gama-tocophe	23926.4613	33923.3345	15831.1132	22691.626	27480.623	34578.6381	59308.4772	23176.6513	69156.9231	161604.658	59565.3822	24100.7658
bilirubin(2-)	7521.22455	3471.89505	8480.7279	15813.0399	25286.5704	27156.1669	1000	5102.2173	1000	1000	1000	5055.9444
C4H9NO2(4)	344376.178	350634.101	356864.604	310757.873	332502.044	336493.242	282750.158	281966.871	284930.238	253113.908	263808.016	270388.154
N-carbamoyl-beta-alanina	2969333.44	3028393.94	3046886.21	2854966.65	3046267.02	3230894.75	2310877.22	2780191.19	2220517.73	2272903.16	2967522.32	2764235.99
histamium	220521.337	175702.907	160293.889	215354.372	197165.823	140408.901	143532.619	113300.099	127607.136	180909.369	123328.454	166562.498
1-pyrroline-2-carboxylate/	288489.857	268376.904	281456.793	253201.454	246256.941	266378.722	210833.22	234963.439	236680.008	201585.032	214693.387	242716.329
acetamidopropanal	65312332.9	60732797.2	61085457	55149292	56129711.6	58598948.3	43551990.7	45190218.6	43062854.8	36531907.9	40522814.9	38827594.2
imidazol-4-ylacetate/thym	442583.672	412838.209	364524.032	387454.54	378499.08	514460.219	183444.773	243211.564	210885.33	161398.164	379052.083	347050.236
Ornithine	1109514.26	1144145.78	1008591.85	1049827.41	1172781.55	915761.513	467419.468	563127.512	467165.789	594639.046	673100.706	674177.473
3-Ureidoisobutyrate/Glycy	236058082	229909855	239152409	228793511	235336898	249478721	170699260	179742376	166400938	154035119	177257525	182762497
L-pipecolic acid/4-acetami	527084.868	545161.055	613356.598	552028.353	589083.387	591853.449	387320.25	478936.024	469681.456	436528.76	463940.256	512883.346
L-pipecolic acid/4-acetami	9257291.11	9531099.47	10314709.3	10161034.9	10867802.6	9725773.11	6126652.42	7931474.96	6207299.17	7716575.34	9577255.85	10809880
C6H11NO3(4)	961004.195	1025389.1	1116031.62	1020466.85	1125180.38	1065732.76	631479.733	868692.491	813976.349	698923.883	926985.575	1055837.13
(R)-mevalonate	1423551.44	1385418.88	1465079.27	1410972.77	1415640.79	1532406.71	1028793.69	1079982.22	1012571.32	970771.842	1080749.43	1117793.27
spermidine dialdehyde-1	981313.441	888085.901	1081659.04	934460.453	986709.122	956309.754	624938.863	484551.149	724754.803	680446.518	480921.207	812463.352
spermidine dialdehyde-2	238831.685	209810.875	227611.651	212794.004	198363.613	206083.901	165401.652	147725.209	171080.125	147480.924	107580.327	160252.769
4-(trimethylammonio)buta	7106044.95	5627931.5	6636631.83	5622700.06	5305400.86	5684408.55	3541003.06	2190950.8	3708477.37	3407548.31	1815823.85	4230974.7
2-methylbutyrylglycine/isc	234938.119	207509.923	224854.359	209696.496	201129.787	203139.888	162130.349	143315.047	162451.858	139371.6	105273.26	153361.101
isoputreanine/putreanine	5391407.32	5000653.35	4283465.22	6337871.26	6050685.42	5502923.25	4397347.73	4045365.47	3905923.48	7693562.66	7531878.92	7711549.75
L-Prolinylglycine/Glycylpro	331678.155	312073.207	315599.073	270160.496	285211.845	293214.464	7095388.55	7316427.17	6197464.15	4666055.08	5835228.9	4551560.05
N-Acetylmethionine	3017.3808	9600.5017	2720.3748	3650.8485	6994.3331	4914.12055	2414.74155	4587.99105	9089.05685	6929.7705	6551.99515	8338.18645
leukoaminochrome/dopar	120058.11	108115.86	88307.6464	116910.249	101129.217	119792.064	233150.574	93760.1506	197238.817	1112871.95	193995.365	92896.2047
leukoaminochrome/dopar	176731.295	186782.279	187306.523	169065.867	201056.433	239345.95	227311.563	157438.131	215507.268	1256859.82	312557.705	201574.697
Pyridoxine/Norepinephrin	132215.964	141781.254	162720.089	146168.073	155896.215	171091.155	109712.718	128893.005	121784.715	138213.387	138373.615	151872.6
Glycylleucine	531791.404	564631.185	517587.397	472821.581	555181.62	503667.446	319196.938	256549.01	325592.964	445741.823	228153.724	290135
gama-L-glutamyl-L-alanine	233140.8	222374.96	204911.14	204012.477	195029.351	231570.543	154910.236	142988.533	162396.581	55820.4853	67951.6919	68383.9065
gama-L-glutamyl-L-alanine	35775.8921	42341.9097	36764.5641	38234.9762	42698.3601	45830.2188	44986.7036	40727.3181	32476.8254	40436.6786	41294.0038	24986.0147
C9H8O3(3)	1296417.3	1263409.68	1433865.32	1237002.37	1350001.03	1412264.52	1096759.47	1142331.66	1072687.9	1098064.76	1173241.93	1303278.17
Adrenaline/L-Normetanep	143414.228	140362.719	147366.249	124870.768	148391.209	158161.255	118374.156	156460.868	131486.396	86256.9865	118555.068	128856.438
pseudoecgonine/ecgonine	71448.3515	59541.2762	49156.2366	41380.167	48462.9334	50301.7343	40606.1395	41720.2088	39715.2763	32546.384	38251.0652	23689.2442
N(8)-acetylspermidinium/N	39782.4694	39606.3303	33952.9462	14156.825	10278.7741	7171.7846	29608.8268	33777.6565	8766.3391	28916.7485	38831.3411	30968.4778
limonene/(+)-alpha-pinene	185008.243	320245.039	138320.363	223299.952	210212.157	325232.088	599644.024	233773.258	550529.874	1413985.92	672086.337	247508.299
C10H16O(3)	300451.731	627316.615	240460.326	425758.218	324984.803	518736.816	944027.414	433405.864	919347.993	1939501.57	1208122.79	409623.72
indole-3-acetate/(5-hydrox	3368802.75	3418000.49	3717989.19	3283769.08	4329701.26	3898817.6	2104679.6	3097883.62	2679156.38	1867708.17	3357297.48	3990827.19
5-hydroxytryptophol/12-d	14470.6837	14362.4957	18543.4614	8033.64	20591.4745	20352.0577	9563.93035	12107.4393	11124.5895	13695.0455	20328.7252	13599.9374
5-hydroxytryptophol/12-d	22345.9252	14223.8273	20222.4612	16741.1218	16847.1259	11452.9072	5332.54705	9878.12545	13950.9352	15219.1493	11516.537	15817.743
NN-dimethyldopaminequi	53737.2211	57865.0281	52193.9275	49586.9147	53858.2059	45675.7468	38528.5858	47287.0798	36377.4693	57182.4488	46001.2235	40532.1675
NN-dimethyldopaminequi	127946.281	194157.101	143674.175	216583.126	144438.045	196067.684	247968.571	103006.518	192025.075	1142809.89	212040.879	136003.166
L-kynurenine/Formyl-5-hy	93196.5891	110061.578	112365.543	97766.5617	106135.507	98635.1266	65779.3716	76425.5742	65843.1061	68156.1339	87831.2327	91971.3004
methyl indole-3-acetate	348527.924	362147.205	396408.945	392136.334	375009.673	381003.883	266958.239	304231.667	276193.784	286040.228	316307.363	359942.912
sphingosine/(2S)-1-hydrox	348224.505	391840.829	310224.788	265502.522	236588.738	189924.046	255980.964	185664.67	178297.904	70795.6074	84069.5877	123926.017
3-oxo-10(R)-hydroxy-octac	172377.112	351245.247	144659.148	235839.957	192678.879	297598.745	584155.775	242179.858	552931.625	1572822.43	673533.48	237829.169
C18H32O4(3)	146687.903	240529.53	92406.005	156488.96	178715.755	298172.471	557809.117	150706.834	608263.44	1591506.17	610222.353	216229.822
C20H30O(4)	1769.0621	2107.874	1000	1000	1167.56085	1935.69105	4196.30725	1756.88865	1743.7772	11249.92	5664.05155	2124.5134
ADP	2483597.85	2559936.12	1428334.3	2055137.75	2025619.58	2971933.44	2816436.47	2692174.42	2085240.06	2012459.84	2796920.17	988115.044
dGTP/ATP	2440074.45	2865113.87	1514988.81	2660844.41	3131521.51	3681966.33	3614336.65	3944080.63	3385660.74	1380315.19	2309379.14	581179.51
tetradecanoyl carnitine/m	233563.103	150714.585	168078.145	193870.888	182382.978	147384.425	139426.067	203820.244	124083.524	197477.358	244609.585	306004.763
trans-Hexadec-2-enoyl car	150284.239	101517.334	116936.527	142998.975	128488.555	128375.969	140598.705	198644.986	137233.598	241696.684	309095.635	398395.302
alpha-linolenyl carnitine/g	11415.0378	4910.81745	4599.38975	9822.4593	8207.3862	7392.10885	7415.6395	13092.7897	6554.7133	10592.1173	12536.546	23612.8623
Linoleyl carnitine/Linoelaic	41094.004	23213.135	26864.1173	33422.8154	34773.2468	34975.9843	34897.1831	58226.3359	38694.5161	92797.9499	116153.276	152883.112
Elaidic carnitine/Vaccenyl	775866.407	554643.647	560080.615	704409.046	722618.04	713469.768	731666.647	1059644.47	761714.446	1175784.96	1415657.96	1936852.9
C27H44O2(5)	166540.267	117065.249	125709.597	149785.363	155194.201	141786.721	162502.264	228970.621	161383.859	302095.003	338659.197	473194.133
C27H44O3(5)	10970.6361	7253.6247	5161.43465	9794.34015	4921.38855	20198.4295	27709.5671	4757.0599	28719.5057	99698.4895	23865.9262	9163.9449
C27H46O3(6)	2808.52375	5278.4248	1952.12745	4861.80915	6237.9154	11766.5952	24848.443	5416.07955	26878.5484	88962.1415	28376.8253	6713.1218
C27H42O4(3)	4100.02525	5605.6283	2337.44345	2972.65235	7036.95855	9498.52815	19454.4939	4782.89285	21972.5227	108697.473	22633.6857	7079.16935
5beta-cholestane-3alpha-7	9891.50185	18149.7281	6340.9048	9156.03345	14307.233	32127.1408	66777.4033	10840.8032	73982.9774	207990.849	71423.4748	18101.5384
imidazole-4-acetaldehyde	219609.915	191068.988	239839.909	243650.838	210289.377	194242.058	130725.41	112691.502	144077.798	167023.395	122265.117	196187.113

agmatinium(2+)	22621.0142	22926.3437	18792.0382	26980.4534	27919.7031	20763.3229	16528.3899	14788.1828	7879.9773	20035.6422	24695.0103	21526.73
5-guanidino-2-oxopentanc	21465.7966	24168.4809	52188.0674	13977.2963	22756.3023	31070.9278	21128.2064	20266.1089	16135.7242	11819.9814	13502.9028	35639.4405
phenylacetaldehyde	13236.6217	17746.4017	17686.9874	9541.2121	18357.2	13310.9433	14952.3193	8583.04415	17846.7797	17898.2439	13919.256	19197.4722
L-serine	3954285.93	4120787.33	4232368.62	3792468.93	3960453.54	4308861.32	3374302.33	3947620.43	3102925.11	2633467.23	3456772.85	3286207.15
L-cysteine	130078.69	137754.119	48355.7138	24340.4632	90890.5996	76927.2836	9022.1682	130738.6	63642.7589	1173.0012	12614.266	172495.264
NN-dimethylglycine	709116.478	485588.111	525078.51	664101.869	576974.647	639872.524	407731.611	372152.174	401699.875	626468.095	391300.007	430202.331
L-asparagine	2969333.44	3028393.94	3046886.21	2854966.65	3046267.02	3230894.75	2310877.22	2780191.19	2220517.73	2272903.16	2967522.32	2764235.99
choline	110820744	110216316	116352989	114450949	118731240	124604648	97997684.4	98387594.6	101838410	178735422	168788561	185720308
L-methionine	10083552.8	9486112.61	11040822.5	10317276.4	11309385.5	11322279	6028876.86	7907958.22	6202416	6526129.43	8879130.68	10732255
Guanine	1256315.49	1195747.43	999148.366	984646.339	1088325.9	1646610.26	34547.3813	42915.4565	35490.4227	42824.3854	59361.5009	97802.8789
L-histidine	33923116	33943258.9	46991267.1	41112545.5	42433994	33212862.1	18695502.8	20802233.8	20903613.2	24877486.6	25499335.1	36581853.7
spermidine(3+)	266702.104	223705.878	192408.745	246310.019	191857.619	185573.566	316032.178	244603.993	223758.277	253301.23	199634.386	157055.081
L-phenylalanine	84613052.4	87039997.7	94837877.2	91192595.7	93931005.6	93569772.5	62402684.2	71067963	62949803.8	67353161.3	78885158.6	88158888
Biotin	406812.175	342248.522	325559.203	308144.901	292651.668	430453.56	147117.262	159234.278	139307.319	231306.762	280978.301	326378.925
S-Adenosyl-L-homocystein	196380.062	161777.547	136123.882	112058.989	99012.6579	138750.678	49544.6936	40477.1417	75063.1301	71755.4414	61406.781	79355.9731
S-adenosyl-L-methionine	342310.481	405499.047	372058.386	327483.532	341698.996	355650.535	261182.566	192289.67	224892.706	272196.152	169471.558	248658.671
Riboflavin	382225.211	411417.907	440522.058	425708.74	482605.005	486780.085	287675.804	339442.825	289577.408	340363.256	421347.59	491541.825
Folate	102997.729	113057.294	105813.069	116300.687	132272.699	132617.321	88613.9657	113658.952	85206.2244	96929.7071	122509.301	117469.822
L-threonine	13946278.3	14198366.1	14642166.6	13356029.4	14414527.4	14924821.3	11066134.4	13450377.4	11058244.2	10692714	14227671	13066744.4
glycine betaine	76923974.5	74928867.8	77869505.7	72844817.4	77540622.5	79369298.4	51420502.9	60383881.7	53186896.5	51217164	61221802.6	66664183.3
L-valine	8414432.96	8408055.33	8611065.73	8449475.01	8288244.24	8362618.24	8318265.97	7171762.27	8501723.74	8348772.82	7249811.26	8342087.73
L-glutamine	236058082	229909855	239152409	228793511	235336898	249478721	170699260	179742376	166400938	154035119	177257525	182762497
L-tyrosine	5699279.44	5666008.82	6063362.17	5382063.91	5998210.36	6024289.49	4760610.7	4935806.28	4631523.17	4614013.51	5144258.41	5603315.06
L-alanine	11796561.8	12000655.3	11898243.4	10432023.5	10962290.9	11878146.4	8178425.35	8346677.65	7792868.91	7669901.02	8303625.36	8781741.74
L-proline	65312332.9	60732797.2	61085457	55149292	56129711.6	58598948.3	43551990.7	45190218.6	43062854.8	36531907.9	40522814.9	38827594.2
glycolate	978553.245	933104.552	989005.652	916364.915	948505.956	946979.78	509619.669	695283.373	644369.913	762698.559	854972.529	1030191.62
oxalate(2-)	234668.25	252920.226	227719.426	253403.63	285841.508	291803.78	338078.421	192200.4	308974.307	843017.448	319332.026	318175.674
Hypotaurine	305734.24	286511.922	281555.727	253906.669	259094.233	323334.792	220152.207	211511.274	198569.709	100085.775	125616.109	135463.087
Taurine	24348454.1	22937865.5	19301857.4	19443450.8	18652095.4	21802280.9	24632683.9	21345214.2	23317450.7	13175667.1	12959539.8	11785946.2
C3H4O2(3)	5017337.21	4701876.73	5516825.28	5089151.91	5501780.16	5268638.4	3743940.58	4423330.11	3910663.3	4361590.17	4995350.9	5859641.25
C3H6O2(4)	1139473.39	1177282.24	1104860.16	994638.96	1110953.02	1185724.16	1051915.76	984188.004	1049053.25	1390643.36	1000161.32	1061428.44
3-Sulfino-L-alanine	694879.161	706048.061	570666.868	611335.058	515448.945	623813.824	951586.644	571629.729	731075.005	749291.926	388269.344	354052.52
(S)-3-sulfonatolactate(2-)	3541.3839	13999.3534	1591.1164	6467.6204	3253.90045	4467.15615	10450.6866	1596.532	5379.5507	30662.1815	5325.95745	2200.85005
Glycerol 3-phosphate	5417840.82	5509873.37	5286993.17	4573610.57	4865184.32	6115159.7	3721406.36	3509312.79	3618498.58	3941227.46	3678400.79	4917999.27
O-Phospho-L-serine	408125.122	346826.164	318895.05	305244.445	268371.872	268039.811	118240.453	92942.7629	88417.8302	52487.4034	48711.2633	49787.2944
L-2-amino-3-oxobutanoic a	291595.134	303907.085	279460.215	267069.413	276027.083	307797.147	252565.047	242037.345	225660.3	247584.663	216891.497	216076.228
3-(methylthio)propionate	200987.894	165808.067	170337.421	188990.598	163726.504	155380.879	186077.111	182064.737	125406.722	174794.392	181725.825	154456.373
L-erythrulose	4623816.69	4388572.42	5320265.82	4847094.92	5337274.72	5083573.87	3536266.12	4247975.88	3602946.76	4462551.53	4888655.11	5795780.54
D-aspartate(1-)	16394321.2	15166761	14847042.1	12848830.9	12731363.4	13849074.1	9993615.17	9844253.96	8974189.23	7377958.78	7899744.14	10344143
(S)-malate(2-)	49859049.8	51806309.1	50854177.2	46784617.4	47137093.7	50526291.5	53509884.6	47048169.2	50364911.1	35352593	37076436.1	43143933.5
L-threonate	6658041.36	6128956.36	6447952.31	6509352.48	6158014.68	6468784.44	5708257.39	5418340.13	5331601.19	4821787.7	4878925.03	5256532.1
allantoate	11169.6334	8597.0841	12593.4592	8559.27695	11892.2324	9382.52225	5436.61675	8343.4662	5619.59625	6662.80405	12373.6942	13835.9544
N-phosphocreatinate(2-)	11015.6422	10758.9243	10269.1653	10838.0728	11658.0878	8163.7279	15566.1132	16233.871	10979.7225	10673.3968	12411.777	9367.33905
3-methyl-2-oxobutanoate	1141537.8	1239746.87	1171634.45	1253373.74	1296063.48	1434293.12	1485641.13	1072953.04	1487499.23	3413759.89	1824411.24	1384291.41
beta-hydroxy-beta-methyl	545924.709	540567.414	535750.665	504101.478	528405.812	525152.714	543307.12	537225.922	547588.592	685511.675	527262.665	544276.899
C5H9NO3(5)	646257.164	590352.662	661742.938	560202.575	530699.181	587649.821	406068.752	450092.899	377680.009	412162.493	488538.717	592658.813
2-oxoglutaramate	294814.921	276797.822	316910.813	294455.666	290084.786	328867.188	201734.161	223957.567	175955.469	158417.29	214155.469	255337.187
2-oxoglutarate(2-)	952218.493	836708.286	858598.395	843433.904	743485.293	754524.004	943590.677	905635.186	866063.223	599313.337	713030.343	754423.025
Xanthine	3053990.28	3336167.21	3706408.82	3276197.71	3715687.68	3423336.62	3147953.49	4019805.73	3264137.91	2880329.77	4868796.76	5267152.21
orotate	366506.396	301532.566	274709.664	372242.867	372020.89	185163.082	35434.6377	54708.221	34531.6696	21638.7495	39488.3846	32238.2513
(S)-dihydroorotate	218635.023	178647.288	201512.94	212448.005	244591.963	162411.613	86701.3745	70767.2807	77813.5641	1486.6332	1000	2483.97795
N-acetyl-L-cysteine	2852.2062	2342.30325	2316.27035	1000	2409.5033	1000	1150.9353	3399.1753	1682.27075	1000	1000	5745.9607
79-dihydro-1H-purine-268	215745.768	196629.38	250815.541	245936.866	255010.63	264961.629	151490.138	182614.854	157817.593	169106.111	203456.907	242533.962
N-Carbamoyl-L-aspartate	292946.475	274985.353	284117.59	333590.453	327526.069	184132.469	133713.785	107396.57	124785.997	7705.5299	13379.2633	5444.8157
5-Hydroxyisourate	30834.0638	26671.8627	34255.2824	27239.2729	27048.5365	33249.161	23844.9721	22193.7918	25611.7663	19727.1649	19089.9701	26863.9776
(R)-2-hydroxy-4-methylper	316229.942	309371.643	278309.851	289496.14	291049.457	297540.061	349281.086	307730.019	321562.044	602824.087	374647.993	297522.716
4-nitrophenolate	248542.624	183152.26	200010.703	199848.418	221296.259	185808.183	182910.316	210374.832	156602.916	87937.2509	150259.679	176730.983
2-methylglutaconic acid	3223234.37	3065003.64	3621676.66	3238517.25	3709539.27	3513913.59	2365959.4	2876952.89	2551005.08	3215041.37	3373039.19	4019839.02
adipic acid	146588.59	161540.237	123451.038	118864.01	136782.428	179577.362	234730.172	128667.877	230123.127	790271.742	209641.907	139205.378
L-lysinium(1+)	10089953.2	9834146.54	11606412.4	10439466.3	11083416.7	11393974.1	6481856.49	7507375.61	6752417.91	8190740.27	9190922	12073830.1
2-aminomuconate(2-)	12936.3819	9257.8791	17633.0446	15159.9282	13004.4891	15392.7024	5265.4682	3774.32375	7061.0836	11856.9911	12637.3013	11943.6975
2-oxoadipate(2-)	23894.6844	30148.7012	23667.7761	22340.4731	30374.9213	25889.1801	16725.6696	24611.4062	21588.0329	29254.4288	26819.2319	27404.5138
erythro-5-hydroxy-L-lysiniu	21234.7762	21042.5765	23519.9475	21091.8857	22496.4402	22087.405	11246.1636	13646.0909	11438.7296	16140.4553	17152.7406	25709.201
L-argininium/D-argininium	4562094.69	4539992.64	5194036.55	4822647.22	5100882.86	5104447.22	2890795.75	3349703.03	3003332.84	3822385.77	4180148.25	5564667.75
oxalatosuccinate(3-)	1207.09525	1163.01615	1861.41075	2389.12535	1000	3617.96745	7091.07065	1000	4020.61285	63732.0244	2465.9423	1000
L-Fucose 1-phosphate	23038.0881	21994.7301	17641.172	20844.6779	26703.8901	32514.8546	32886.0069	37690.0523	32252.6526	24341.9601	27341.6293	24499.7974
D-Glucosamine 6-phospha	77270.4875	78888.9344	66722.5584	60810.232	60630.4228	62805.6472	82956.0394	81485.1384	66752.5409	51609.4361	62386.0733	62414.0611
6-phosphonatooxy-D-gluc	68467.7873	71296.7161	37550.1935	58097.3103	62768.4251	80571.4409	98809.2684	103096.343	75020.6954	34408.9368	52383.7505	15000.6583
4-hydroxybenzoate/Gentis	166971.259	141023.129	160018.179	147266.875	157252.413	140944.76	135564.017	156992.396	140326.195	131732.7	127294.941	147546.866
3-hydroxyanthranilate	48539.6643	39139.0407	41215.0224	35320.985	37766.9886	33029.8314	33959.2895	43972.24	28309.2285	15011.3385	30922.7883	35148.5398
quinolinate(2-)	37016.5151	44774.7872	38850.9088	41935.3071	52214.026	40705.747	32462.0336	34971.0064	21566.6264	52509.3107	54162.1528	65763.3383
2-acetamido-5-oxopentan	48549.6198	48092.1998	51940.0729	48101.8462	44722.7575	51924.5803	65348.0941	57664.7027	57178.9452	75600.2968	46005.8379	46641.4468
(Z)-but-2-ene-123-tricarbo	19174.8853	41253.9569	25754.3538	22778.6986	29109.5915	45179.8242	46489.8881	18816.4865	30567.3458	128285.307	39074.3105	36621.118
N-acetyl-L-glutamate(2-)	411727.266	348088.146	510946.193	504656.742	375064.489	342036.938	266116.146	234008.626	132821.646	145526.381	184233.744	222145.527
L-cystathionine	15087.2474	16526.8626	15128.806	15467.749	17341.0273	17192.0532	10273.8191	14365.0782	10710.1498	13613.6285	15990.2345	16597.06
Sedoheptulose 7-phosphat	258110.865	237632.797	213314.545	190979.27	192532.558	198128.26	243902.844	236913.747	235853.463	104161.494	102177.977	99670.365
sedoheptulose 17-bisphos	2569.4317	3374.3338	1000	2910.9354	3015.7016	4431.0458	21403.8758	15330.9499	15449.7348	2992.6287	4809.4193	1000
dopaminium(1+)	12078.545	11799.5179	11184.0173	12971.2761	12813.1602	16101.4199	8789.30875	12071.2909	9668.85125	18684.4306	11855.2372	10847.1937

suberic acid	124444.885	139568.684	90646.0035	93221.623	111878.39	160566.887	283559.569	114283.842	249265.697	960246.241	227292.622	119751.627
4-pyridoxate	245218.098	214422.636	262966.242	240808.453	257503.83	194051.348	107945.97	196622.332	126128.822	76404.6577	174291.072	221189.827
lipoamide	14997.7427	10120.0642	13655.58	15990.727	14368.3177	14429.5804	6385.4455	5258.2673	3484.9484	5572.43095	6935.4086	6006.818
lipoate	360900.343	348038.529	347125.489	335221.201	338654.859	375063.538	352182.8	230602.753	321231.021	593882.486	277701.072	427104.322
fructoseglycine	11668.4765	8404.7087	16301.4733	15036.2046	11296.7103	8344.55235	10660.7527	9632.2151	6984.018	10730.7961	10105.8054	14602.3955
Sulfate derivative of norep	85762.6799	72869.8822	116133.78	96177.4529	93321.9806	88111.8568	97903.5687	82232.2681	43501.9352	12602.9063	71681.0084	57006.237
gamma-L-Glutamyl-L-cyste	72487.2988	29781.3893	37119.7692	25572.4891	31334.2502	36666.5281	135621.165	197868.964	134456.098	83285.5532	115321.212	263645.672
nonanoate	9009778.64	7396843.36	6945083.74	6951325.8	7089637.62	6799655.87	7576426.41	8111771.73	6583520.44	5913742.09	6398242.11	6714074.37
(4-hydroxy-3-methoxyphe	115614.905	110399.315	114781.784	106580.234	112957.298	120839.558	108338.884	102545.863	104659.083	186102.605	117189.575	112890.599
4-hydroperoxy-2-nonenal	544154.762	685996.491	399824.123	480826.571	572610.234	794001.854	1685359.31	568357.742	1459249.55	6992725.72	1567947.63	602938.301
Azelaic acid	171495.735	184676.85	129802.129	150233.263	136774.032	198286.605	372668.904	162683.332	255151.208	932940.478	256809.975	121955.574
(R)-Pantothenate	38852875.9	40488462.7	40709057.2	37578709.9	39724058.4	43397121.2	44807831.9	45707928.1	41768256.4	25487931.2	34708216.7	36322757.9
carnosine	204560.476	189091.585	286834.377	231021.156	234656.046	226652.012	151691.568	167913.108	151716.641	188588.217	201970.832	341848.247
Deoxyuridine	96941.202	78187.5267	80445.4068	77098.853	81782.1497	88856.9332	94143.6245	110084.526	96038.396	155499.108	177372.459	190273.091
gama-L-glutamyl-L-alpha-a	11610.3212	16863.4312	15543.5809	9589.4303	9892.65475	15135.7568	14944.6872	9689.8208	13420.6255	19228.5423	23531.9615	21598.8487
Galactosylglycerol	2734.81685	1145.2366	3182.3689	1000	1000	1000	8417.3534	1000	1780.65255	1000	1000	1561.72895
S-[2-carboxy-1-(1 H-imidaz	55305.9849	58332.1831	63375.5636	56315.3049	53287.4464	78749.4375	28400.2947	39389.4718	35614.1	31537.2258	58209.3385	63504.5349
N-acetyl-seryl-aspartate	70805.2968	71803.5635	73894.0458	74830.1044	61470.2891	82380.144	72604.957	69432.1666	60506.2766	59130.8234	54141.2321	76428.658
2-keto-3-deoxy-D-glycero-	59440.7098	60043.6136	72514.5659	62339.3784	62641.7908	54791.6398	50763.4369	51140.5733	43562.1575	25259.6649	42433.6726	50567.0493
dUMP(2-)	132546.841	327154.671	393204.471	163710.757	318480.736	377393.328	50051.0814	222766.996	255183.925	39174.7569	170346.142	228622.135
CMP(2-)	329510.317	242481.413	171834.65	184267.93	152563.153	239895.511	154732.44	123008.63	152597.177	239926.939	172655.243	233726.212
UMP/3-UMP	1780663.28	1351202.81	920370.285	919934.171	815027.719	1360284.92	1078679.2	639440.693	914213.294	1443650.83	879158.99	1496725.29
5-Amino-1-(5-Phospho-D-r	31582.1551	27040.4844	34587.7747	31005.9534	20336.2497	27680.0859	52381.6401	52767.0328	36870.8194	25383.3955	21409.7408	37571.0226
2-keto-3-deoxy-D-glycero-	180371.81	146589.722	106848.695	125414.882	110406.311	168034.581	152086.632	120656.169	124596.927	172134.696	127812.295	156655.893
Perillic acid	44302.7119	67870.4585	32062.3237	48495.0925	51455.0158	81727.4768	165610.825	49158.9629	145339.129	558613.518	151669.043	63701.102
trans-45-epoxy-2(E)-decen	51390.3468	67343.5516	38694.7639	50154.5821	53042.1272	80025.3007	148531.867	49058.4308	126848.498	471219.986	138483.146	57401.7619
caprate	1682722.16	1434348.6	1378961.1	1420388.14	1388919.78	1371602.59	1383962.49	1556997.97	1264553.74	1432173.18	1342991.94	1356475.26
w-hydroxydecanoicacid	47027.5471	66763.5613	29423.9444	43092.5616	44716.0469	59722.0193	112714.9	44095.908	114721.012	351657.367	125377.634	54480.7784
kynurenate	100468.902	98716.8416	103842.256	102616.871	105083.873	104251.82	70854.0826	98056.3936	61417.6519	46456.8524	89394.6611	93205.6537
o-methylhippurate	1318386.73	1242699.87	1464173.63	1477584.72	1597484.39	1576205.8	598608.933	979969.107	632856.688	494483.536	910123.229	1194105.86
sebacicacid	159421.033	269118.953	109492.178	163359.628	186451.365	321580.746	815951.02	157265.074	661491.13	3243176.03	656197.22	210719.447
Thymidine	548853.908	438271.347	342887.775	294819.427	311592.943	506377.735	385349.62	403662.259	362254.081	446000.343	571127.737	600545.484
N(omega)-(L-Arginino)succ	41792.6026	33593.6652	40663.8504	29566.7608	28661.8202	35781.2376	31632.8701	30644.115	28468.6752	24223.0918	24909.9341	31477.7586
2-deoxyinosine 5-phospha	5949.34175	23471.9213	15665.1652	13810.5969	16713.6322	21466.8475	8595.7198	19723.9015	21431.1349	8550.5617	23161.0248	21374.9314
L-tryptophan	8366564.69	8406317.76	9301995.24	8861102.83	9182471.06	9193865.25	6066311.57	7218820.92	5943216.38	6551550.78	7952062.39	8560996.66
5-Methoxyindoleacetate	108689.077	105899.628	132452.91	114963.988	125248.427	114040.384	58168.4156	94044.3181	65435.5451	51920.4759	87300.4131	118854.767
salsolinol 1-carboxylate	66697.5658	95921.3377	84632.8565	98064.2062	91532.4398	80197.238	46054.2489	55221.0659	42197.5633	67629.5067	51384.1214	50176.5537
laurate	2087420.12	1683773.73	1580337.29	1650576.18	1762283.8	1786574.04	1688005.81	1581657.17	1297474.97	1929438.34	1503903.9	1942979.18
omega hydroxy dodecano	55673.5169	75415.06	28900.6255	45935.9827	44377.5043	67192.5588	134826.573	43529.1128	146778.026	390467.871	170742.382	66505.6887
Dodecanedioic acid	238963.013	388285.047	197242.977	309543.266	305578.804	445696.062	861402.283	200241.556	832588.127	3232487.29	954771.771	254075.361
1-deoxy-1-(N6-lysino)-D-fr	18189.8145	13952.0329	24231.5825	20547.946	17619.1857	17470.867	10811.4298	9704.3127	4938.58795	8281.5445	9602.8096	14765.3649
tetradecenoate (n-C14:1)	66104.7153	74710.4401	53929.4618	58229.1624	61468.9888	67299.8907	111135.425	55262.531	94759.6939	333476.849	113700.494	73786.5334
myristate	1565514.64	1334492.05	1300014.61	1284099.09	1360373.79	1328275.52	1126246.6	1191556.16	1040501.2	1081514.24	1216423.08	1356169.39
6(S)-hydroxy-tetradeca-2E	31257.5475	41360.5302	22991.7685	37531.8238	32332.4781	42326.794	78155.8039	31883.5557	60833.7525	185821.187	65505.1332	37809.8798
omega hydroxy tetradecar	36297.9879	56344.4216	26021.2987	36171.0989	37005.2108	53073.6468	80546.3919	36377.5464	76905.5092	194575.854	104222.414	46962.0213
3(S)6(R)-dihydroxy-tetrade	112830.807	172400.445	87720.5108	113649.736	126805.774	202208.94	540746.45	128891.065	437106.961	1683252.9	458770.436	185412.831
pentadecanoate	694374.68	644432.922	486143.645	629357.331	622891.828	738281.807	740669.883	492819.214	645247.986	1298078.44	1004205.23	621572.561
palmitoleate	400061.273	362775.246	398440.422	296384.783	387505.124	318615.149	218486.123	295677.756	195065.593	230716.831	343344.394	440267.829
palmitate	21715848.8	15941067.5	18736972.8	19236617.4	19785561.8	19581107.6	15735117.4	16540009.8	12168638.5	10727234.3	15140869.1	19413429.5
omega hydroxy hexadecar	294021.708	334888.769	267963.999	302174.186	295976.006	326491.649	352480.43	289084.843	348036.121	550080.791	487477.933	314769.291
3-carboxy-alpha-chromanc	27459.2269	31612.9381	22729.5333	23391.7487	22415.2277	28416.8464	20895.8988	24080.4447	22532.2874	36284.2663	24189.9735	25350.8366
hexadecanediocacid	82391.8836	147626.61	60640.783	94338.5081	100969.358	176692.74	363445.604	84107.4759	370872.96	1279472.61	392047.932	127315.738
margarate	410905.788	356701.325	292732.818	363900.608	392821.196	411550.043	430959.711	331715.751	368124.172	624770.907	481413.996	340531.506
stearate	9706084.09	7100945.11	7406438.67	8560353.28	9130822.83	9699650.95	11026959.4	8751923.14	7653752.8	9593658.18	8989437.07	7855592.28
12,13-epoxy-9-hydroperox	57374.7434	116921.422	34991.2767	66370.3297	74025.2024	148019.666	348418.122	67767.7343	343582.546	1362469.6	354519.419	89595.0625
estrone 3-sulfate(1-)	1201.4397	1505.14495	2042.89385	1946.2656	1815.17025	1380.58505	2571.0204	1511.5211	1727.6778	25934.6531	1328.43675	2527.78645
dihomo-gamma-linolenic a	122905.769	93327.6263	131213.62	93841.4084	106215.618	105170.19	55447.123	125084.764	64340.6155	55481.0851	188222.348	227713.047
11-cis-eicosenoate	81869.7148	63293.7401	57285.3805	57917.1191	62065.7724	82963.9939	90063.7499	72229.2123	65437.1506	134979.861	75871.0151	69239.1922
15-hydroxy-(8Z11Z13E)-eic	9746.39215	10523.0014	10310.5793	7624.7143	10529.5664	8884.7514	9404.7395	8401.83415	7993.57995	30021.7087	9960.23035	13537.948
CTP(4-)	259407.34	269106.548	133380.847	283678.782	342598.012	487140.838	520230.056	512663.165	440824.738	109954.027	190367.728	40169.353
UTP(4-)	1197068.57	1188757.29	654821.424	1223963.88	1362716.53	1887727.52	1655902.69	1681057.8	1454219.46	447395.964	636556.175	151626.506
(R)-S-lactoylglutathionate(	1626.12765	2985.7858	1000	1000	1000	1705.95055	3709.8475	4333.0044	1000	5868.61875	3599.22595	1000
UDP-alpha-D-xylose(2-)	33710.4894	35531.8115	29949.4624	31554.8096	26017.7535	40845.7587	26938.37	25895.8982	20501.7753	20876.8102	20754.4533	11607.5797
D-ribofuranos-5-yl-ADP(2-)	1814.6372	3364.19255	4962.3199	1658.355	1518.49	3249.57865	1000	1000	1000	1388.3507	3583.11315	1000
UDP-D-glucuronate	18997.3911	26348.2084	8521.64105	23089.3015	31691.4454	48544.0479	52621.5067	45059.5696	37848.2131	25726.6907	38213.3232	6360.3972
GDP-alpha-D-mannose(2-)	40514.7176	43137.5621	16109.0277	32021.5453	44915.6867	43506.1484	54186.1853	37279.3494	43951.3144	13142.5175	14775.8219	5021.82965
NADH(2-)	237086.368	203525.51	158030.278	146571.484	175679.972	247265.009	182458.657	399469.389	183437.92	120806.276	252733.353	91625.9678
NADPH(4-)	40113.9035	33241.2324	18541.8153	41616.3905	41349.302	66095.5941	53096.6215	1000	45764.2461	33753.6059	1000	1000
docosanedioicacid	43923.1364	74565.5197	23561.1568	41541.6875	55125.1387	107061.084	237120.743	36842.7155	217804.321	885180.324	243364.396	59184.8039
glycochenodeoxycholate	39830.2637	37432.8731	53562.6252	51594.6089	51839.4496	46221.2879	64510.5804	80556.5557	54753.1073	43041.5534	76228.8354	83166.2475
glycocholate	22602.5303	24378.8287	35717.5558	28274.6141	33429.5429	34918.8122	36010.1567	40200.9147	28725.3003	38123.5642	51210.933	68200.7053
taurolithocholate	22486.4624	16961.8569	28025.2461	25865.6697	23648.608	20267.9324	17171.5654	24408.0954	16565.2142	3746.3668	26223.4825	35919.656
taurocholic acid	20728.6595	15677.4957	25336.459	24901.7068	21583.4495	18502.9564	24039.2988	29696.7907	20612.4284	18614.5223	34263.8858	41115.0147
cholesterol sulfate	47218.2392	72724.2379	54057.4438	71007.4934	51601.7167	61444.1671	116653.933	80844.6649	67579.5695	44764.6356	66978.8827	53729.1945
13-carboxy-alpha-tocophe	1364.04125	1000	1000	1623.3473	1000	1149.17115	1515.3768	2891.29845	1144.088	2321.21175	5619.57915	6241.83865
3-oxopropanoate	575270.797	618831.692	647953.363	567995.712	663412.399	664233.78	409944.958	546559.49	521695.525	692601.938	526508.187	592217.254
3-hydroxypropionate/D-gl	19212073.5	19320601.2	22729616.3	19509804.9	22728937	23343221.3	13105366.2	16831083	15824305.2	14452188.2	16578838.8	20902140.8
Malonate/3-hydroxypyruv	163837.249	163242.522	141275.246	149114.281	162278.13	200644.447	340029.669	122883.99	271059.649	1313487.67	233925.704	172055.93

D-glycerate	391196.114	367817.208	406224.495	359407.953	371158.272	411950.472	329269.585	362917.227	298237.334	385976.748	401614.324	406826.253
(2R)-2-hydroxy-3-(phospho	401827.446	401576.609	260420.446	354798.608	360546.244	412861.089	653916.588	368823.067	533553.786	247447.61	256001.776	127219.591
23-bisphosphonato-D-glyc	73705.4073	71696.7649	76879.9406	67174.3319	68910.7305	72054.7819	82649.2003	72374.7254	71989.2114	62198.7149	67169.1228	64594.4693
C4H6O3(3)	6618489.43	6831293.67	7679815.92	6903572.56	8020396.18	7460687.5	4968276.31	6460545.81	5555614.08	5711737.35	7099660.62	8033267.93
C4H8O3(3)	1698276.27	1741021.1	1881956.21	1681300.79	1758783.12	1851844.42	1435472.92	1641678.2	1499475.54	1814670.78	1837064.55	2007656.88
L-3-Cyanoalanine/56-dihyc	292461.844	276029.239	302592.762	273791.414	271452.383	294692.511	214712.485	258549.037	203723.237	226181.747	262413.453	297175.169
Methylmalonate	10080860.1	10289107.8	9704497.43	8136586.26	8922022.18	10801257	8313352.17	7767346.14	8391435.38	7832492.44	7295505.84	8859583.75
5-oxoprolinate/L-1-Pyrroli	92939862.7	91754999.8	98542600.7	97690999.7	94099594.8	99225859.9	66828612.6	79482984.8	67297879.7	58702126	84150975.2	97797394.4
itaconate/Mesaconate	1118195.29	1059289.95	1078723.74	1215608.25	1135758.8	1110122.19	1092847.7	929785.063	1095079.12	1693924.54	1148979.16	1099666.3
L-4-hydroxyglutamic semia	66678914.5	64557832.1	58366742	52829073.2	51845721.5	62847056.4	54100364.4	51081829.5	46496966.9	44121393.9	49386110.6	51009466
C5H10O5(6)	945917.058	912124.784	1117587.56	990879.429	1123750.89	1098530.83	719236.593	887210.241	751383.166	867826.58	1006664.18	1195984.14
L-arabinitol/xylitol/D-ribitc	758672.405	782565.515	882103.531	788187.666	774802.559	856176.719	543114.741	648512.525	559658.54	562682.08	733612.15	836129.794
L-xylonate/L-lyxonate	744169.275	732782.284	790159.465	680841.309	736576.573	768688.851	551650.572	547808.758	507004.913	553344.056	570580.109	746792.976
2-deoxy-D-ribose 5-phospl	28023.4138	24451.1333	22826.3071	17599.0336	20333.4168	20391.8729	19695.2888	17620.417	13438.6297	15831.3423	11767.5449	20150.101
C5H11O8P(5)	212426.69	228483.938	173558.147	166261.385	185086.919	207277.403	212266.203	232411.094	205623.189	107772.288	114859.371	120531.962
1-piperideine-6-carboxylat	58851.5768	55876.7265	80770.1079	71299.2088	66317.0081	63099.4082	47250.8989	59976.8253	42722.2048	33070.3706	53401.7839	55638.8387
3-methyl-2-oxopentanoate	4383766.28	4440276.39	4645365.05	4719258.11	5129270.15	5122765.34	3940094.46	3722782.29	4189542.47	6586226.06	5692275.25	5298538.65
L-2-aminoadipate(1-)	789319.102	797569.059	748504.938	703622.694	695348.631	803159.114	676558	630153.439	588724.6	419092.801	477413.018	465575.546
L-fucose/L-Fuculose	165400.138	164642.058	177850.995	172308.524	179716.155	188920.296	133320.777	143405.648	130445.869	157968.22	227141.203	196454.153
cis-aconitate/dehydroasco	1551885.29	1414695.33	1440114.66	1785407.14	1613928.41	1433691.82	1517069.99	1234877.34	1539728.75	2577573.25	1566935.19	1489341.93
N-Formimidoyl-L-glutamat	20303.0874	32169.3738	18127.9457	27132.1486	17347.692	23492.8939	50143.8091	26943.6457	32744.9266	45037.466	16357.4484	12175.3097
N-acetyl-L-aspartate/2-Am	877599.202	681582.486	860151.106	901653.522	767924.092	938164.338	319821.899	299925.628	273419.375	554104.735	672306.544	777162.62
D-glucurono-63-lactone	319196.417	268479.192	391846.434	466141.753	384085.059	345278.739	242355.591	216194.074	188784.391	416966.952	190327.988	218086.899
3-Keto-beta-D-galactose/L	100308.534	110308.96	114446.553	116460.208	121866.895	120735.049	111018.136	94193.7393	114623.105	311368.176	130339.629	148811.038
C6H12O6(5)	2389942.42	2219275.74	2770346.19	2437872.9	2505248.84	2559495.19	1555550.05	1960096.78	1573858.38	1890795.86	2277934.98	2771575.73
L-Iditol/D-glucitol/galactitc	2253472.22	2198783.69	2469431.99	2314667.01	2403206.66	2318715.77	1719209.27	1987364.02	1762820.78	1868327.49	2226478.76	2566753.96
citrate/isocitrate/Diketogu	59087744.1	51537742.6	49540367.8	60417799.9	58560039.6	55713568.9	49082734.6	37290451.7	47089908.3	52226725.8	40226685.2	48214890.4
3-dehydro-L-gulonate/D-gl	80330.3018	88894.084	84386.8963	83345.3881	89459.2119	88786.0338	73147.131	69016.1969	65931.1475	87427.9386	99383.7868	120838.425
L-gulonate	1166984.79	1161769.2	1323405.08	1068700	1103080.19	1151782.76	1821478.15	1854730.3	1953044	1868066.47	2298436.78	2857494.29
D-gluconate	1166984.79	1161769.2	1323405.08	1068700	1103080.19	1151782.76	1821478.15	1854730.3	1953044	1868066.47	2298436.78	2857494.29
Hexose phosphate	1381952.27	1283906.11	1196394.87	1103847.07	1203692.68	1161718.37	1206602.75	1202347.22	1091113.95	412416.12	564561.919	408269.822
Fructose16biphosphate	220425.734	234929.258	111196.616	249454.849	252133.617	282858.302	736876.306	413900.269	597128.34	341364.738	247065.593	96963.0022
(2S3R)-3-hydroxybutane-1	360900.343	348038.529	347125.489	335221.201	338654.859	375063.538	352182.8	230602.753	321231.021	593882.486	277701.072	427661.65
phenylacetate/(4-hydroxy	2712657.8	2716470.23	2811939.85	2844379.27	2844293.25	2770717.42	2205481.23	2541375.83	2252311.61	2142157.49	2545370.98	2731848.16
4-hydroxyphenylacetate/2	729081.397	584478.192	565104.262	602972.004	573143.131	594531.189	643478.753	640076.555	525637.009	780209.971	560466.526	569100.667
N-formylanthranilate/nora	58388.0539	45801.4575	51775.2343	52535.9037	51621.9858	48476.6448	35544.3255	47329.8406	35776.5291	22698.7331	38477.0746	43471.48
C8H16NO9P(4)	159900.658	142285.998	118454.769	116890.436	115199.366	144708.742	86921.291	91616.2759	83316.3402	56683.0933	60228.2541	55291.8691
4-oxo-2-nonenal	211842.126	384382.201	143416.852	249949.737	251088.998	477342.593	1035095.97	251480.215	886673.034	3173330.83	971198.624	308986.58
4-hydroxy-2-nonenal/3,4-e	284663.166	308834.792	243185.516	268978.159	283267.131	335374.343	517277.937	268432.616	463553.373	1436809.87	489503.876	290463.829
N-benzoylglycinate/adrenc	4556158.79	4716448.63	5984110.45	4729513.1	5153205.08	5675860.42	3392784.23	4675986.14	3437989.04	3131307.67	4936202.61	5906236.18
trans-caffeate/3-(4-hydrox	27056.0191	28643.3113	26791.0254	25812.3791	23170.6094	29206.0878	29943.0164	26216.4356	26410.7794	23768.6072	26815.8763	27831.3714
Homovanillate/3-(4-hydro:	792951.701	792343.039	840460.031	755434.257	784432.63	843149.922	697879.45	809414.627	629768.929	509189.988	730936.388	754546.227
2-Carboxy-23-dihydro-56-c	157887.897	191028.522	765361.946	414404.399	618254.514	714069.403	99287.23	440681.473	76898.6836	201509.468	631758.83	814193.31
cytidine	15864.5502	12854.5679	11863.8223	23921.457	28723.1404	28201.311	28048.0274	25059.4813	26782.8122	83547.6069	64727.6051	72116.8361
dGMP/AMP/3-AMP	5313342.87	4167665.95	2815685.4	2958290.48	2536391.02	4610375.51	2762681.05	2173223.24	2432935.52	6031528.48	4633874.03	6749030.31
4(R)-hydroxy-dodec-6Z-en	203449.073	330381.443	112446.453	200769.694	234475.65	423164.659	947665.108	195351.743	845121.139	4056554.08	880423.672	280141.296
C12H22O11(6)	49146.8192	35091.2239	51760.29	47132.7663	49108.4285	53410.2517	38068.4452	48842.1371	31675.5052	52255.6556	40091.3085	50466.6295
C14H24O3(4)	42121.113	68004.1523	25001.7623	46736.0635	40790.9365	81970.8647	190849.672	36927.2762	153098.721	570131.556	169890.624	57690.5358
3-oxo-6(R)-hydroxy-tetrad	107068.616	184566.446	66733.8395	111883.663	123441.145	216022.594	472415.987	104975.585	428779.176	1539804.15	441095.673	139372.46
8(R)-hydroxy-hexadeca-2E	6237.5549	6528.41265	4118.8145	13145.8951	3008.9233	3938.84955	7390.2686	5623.8759	5671.72195	14493.1056	5878.82895	5328.7606
C16H26O3(4)	7777.6294	10272.6521	5655.14345	7071.7178	5061.51555	8602.05665	15630.8857	6473.66195	8524.71485	33806.9918	11491.9754	6986.13545
3-oxo-8(R)-hydroxy-hexad	31031.781	36088.9254	8903.67765	32723.455	23443.5998	40297.2281	96697.6787	18820.2888	78669.3214	313532	89124.1866	27515.9816
linoelaidic acid (all trans C	2030971.19	1254122.71	1520973.45	1301901.01	1482833.06	1668011.44	1277563.95	1187781.47	649625.415	751756.856	852478.881	2116258.25
trans-vaccenate/elaidate/	2750829.6	2102012.77	2597451.78	2228682.22	2496057.87	2351342.5	1443789.1	2145688.39	1273437.76	1119103.26	2064444.58	2747443.71
9(10)-EpOME/12(13)-EpOI	57260.1766	54605.0108	53143.3753	52014.3304	57503.0344	61257.8149	81430.5786	48035.8707	67543.4317	213505.295	71780.7213	58240.1407
9,10-hydroxyoctadec-12(Z	133886.053	217189.285	100298.343	142647.57	160183.544	247725.788	491872.507	143675.106	508058.39	1672291.19	587370.489	187858.969
pristanic acid/pristanate	512308.267	552548.276	319240.865	587521.405	605765.492	822612.874	948148.977	456819.869	806053.121	1207765.03	1107508.83	585527.178
C20H30O2(4)	99064.0974	77639.7837	110562.203	86035.7023	85132.0515	78069.5478	55327.747	96108.5143	55664.2964	39269.0204	132881.679	156520.377
arachidonate/eicosatetran	392521.603	324738.407	441107.273	327592.56	381645.98	347638.618	200468.823	417068.393	222956.294	238768.72	761738.431	876876.922
phytanate/arachidate	140209.132	131134.644	88891.7569	136994.393	144364.429	185854.191	299601.245	158189.128	194642.349	306958.129	214998.485	118678.369
C20H30O3(6)	12676.7944	12167.2474	17960.3249	12257.9761	10813.3685	7602.97795	4574.7159	9658.039	3567.56525	4527.2227	5134.0452	11653.26
C20H32O3(12)	28590.3853	26783.1964	37131.6732	28563.307	29173.4727	30012.7424	13834.2924	26810.8343	13774.879	9691.8515	20831.1899	37475.3554
C20H30O4(18)	16972.14	8741.66735	19073.2096	15831.9069	14804.1968	13371.4324	6231.49205	12766.2815	4899.2245	1970.4077	6066.14825	15520.6122
C20H32O4(21)	73495.7617	180665.053	82637.0674	68784.5619	106587.838	131741.18	37392.4982	55959.6168	25507.791	59583.0033	96085.3196	103856.641
C20H34O4(4)	342166.682	229172.161	259126.985	312969.378	278006.043	254643.806	219090.269	272522.21	185900.941	264070.277	175672.938	235545.861
C20H30O5(11)	27001.9719	13685.685	28002.6673	25804.4915	206621.613	42198.729	30122.7901	8544.62895	7516.67065	22904.2375	15290.9963	14085.8091
C20H32O5(15)	40654.7236	58780.2089	44987.4558	38485.4346	46546.7754	53308.2093	42416.3938	31434.3276	29063.9343	122545.324	48605.542	40392.8733
cervonic acid C22:6 n-3/dc	398907.065	337365.907	455115.449	350644.23	384994.814	357323.297	215959.12	417522.64	237355.513	221745.961	563529.436	688579.613
clupanodonic acid/docosa	138946.995	112859.91	158872.468	115859.646	128224.145	136450.222	78427.8303	161134.391	88225.8091	92066.7343	331071.721	351314.174
[(2R-3S-4R-5R)-5-(2-4-diox	1573802.02	1525458.29	1137024.67	1383608.76	1250412.57	1562961.76	1646531.9	1390361.06	1349027.09	964391.865	997383.26	645701.208
ADPmannose/GDP-L-fucos	51086.2342	55021.1485	26767.2724	50973.0025	54573.7422	79095.8137	66881.6367	63828.7974	56117.8292	54804.9914	72982.3961	24750.2816
UDP-N-acetyl-D-galactosar	2547891.13	2488157.43	1890878.17	2077832.68	1781625.34	2452737.78	1893607.14	1593297.92	1504532.06	1067696.35	1046452.49	756419.239
Taurodeoxycholic acid/tau	153028.484	137117.43	212018.99	175740.589	193097.228	200749.229	228182.832	320076.803	238152.926	214135.339	358658.598	438531.772
C27H46O4(3)	18255.9437	2846.873	11284.243	5861.4031	8759.33585	8008.2418	3506.9048	5332.0162	1000	1000	2164.92455	1824.63165
L-isoleucine/L-leucine	114063698	114516163	125219079	119382508	122608059	128906974	83940812.2	96929869.6	84525220.2	92475103.9	107770804	121386307
thiosulfate(2-)	210436.283	244552.204	155258.4	334099.323	356777.834	510427.926	321090.853	294584.592	250451.792	205774.236	438117.322	116840.579
Diphosphate	586161.984	500644.44	188275.788	306453.541	309823.345	730936.276	413924.14	418713.227	299110.308	541204.013	706417.193	220925.461
Carbamoyl phosphate	138957.58	134180.713	113967.929	121453.957	110739.442	122256.799	143500.84	139559.16	123209.19	108419.832	110985.901	92332.083

glyoxylate	404086.825	400170.72	444257.48	395065.96	435599.643	418612.157	347761.239	359312.4	359593.106	343711.148	374994.283	422246.769
ethamp[c]	26392.9946	21479.2136	21838.7263	19483.6763	18510.5818	22980.6976	95189.4113	90304.6455	100387.735	2335917.39	2107169.43	2074404.82
L-cysteate(1-)	4215.80645	2558.73895	1181.9282	2058.2164	2770.35265	4827.4012	1420.08675	1073.4661	1971.89045	8258.55245	1727.49075	3520.04835
Oxaloacetate	32256.4369	38926.8138	26140.3996	31941.3841	36680.2198	49042.6006	132136.475	15552.7595	118430.116	874752.015	99711.0559	40517.2635
Picolinic acid/Nicotinate	123682.156	157167.213	174379.47	132046.84	168209.866	171804.175	113951.012	140100.427	157021.271	127622.125	158945.487	183308.342
D-glucarate(2-)	19833.9701	19627.2497	21332.0023	18736.8354	27961.0356	30216.7444	23407.2344	18374.7885	20336.0123	23854.5598	21747.6816	34764.3178
Deoxyinosine	32821.0228	32320.6776	30390.0977	25693.2068	25176.4325	42967.6195	18215.9462	17874.5362	18003.6693	48023.8202	32545.4544	12054.6788
dCTP	24829.0283	23971.8719	13149.9837	24561.4466	27804.7148	36532.4849	53057.2488	43360.8934	37964.0991	8413.2175	14636.0052	2463.1998
pyruvate	5893322.11	6902886.63	5168727.89	4029427.59	5889936.5	5309917.84	5184647.25	8759642.96	6670761.96	7791412.79	6871552.75	8582751.29
sarcosine	773292.959	874920.277	901747.309	797597.217	876536.745	961492.065	626895.959	773744.499	805766.851	683266.977	802154.629	841417.422
(R,s)-lactate	441701208	475556721	489075472	457220925	486847855	522307409	304723230	341957612	320557659	207337985	251140500	274831949
acetoacetate	465274.353	811034.494	674108.52	511926.145	784789.049	789487.09	662317.139	703699.561	942084.26	1966382.67	970778.248	686595.681
L-glutamate(1-)	66678914.5	64557832.1	58366742	52829073.2	51845721.5	62847056.4	54100364.4	51081829.5	46496966.9	44121393.9	49386110.6	51009466
D-glucose	23349814.4	21695761.9	26054596.8	24083966.3	26860860.9	25279968	16805508	20686205.2	17260328.9	20416971.1	24142804.8	29122028.6
myo-inositol	2295637.44	2118661.18	2652003.91	2331077.15	2382282.08	2441905.1	1496152.86	1878484.75	1504909.6	1809838.57	2175100.94	2636477.08
uridine	7381720.22	6233810.46	4268749.29	5009559.05	4318868.72	6951666.92	4053072.51	4026946.16	3853726.81	4934308.37	6815122.57	5506112.06
uracil	470983.932	460290.669	494843.293	434667.55	470997.458	447013.281	342182.079	412029.722	354572.194	329613.158	436377.203	498619.421
Fumarate	5222040.06	5301518.11	5123087.26	4786024.09	4691156.44	5143056.75	5484124.59	4838351.9	5144588.78	3584048.91	3727292.32	4211937.6
L-aspartate(1-)	16394321.2	15166761	14847042.1	12848830.9	12731363.4	13849074.1	9993615.17	9844253.96	8974189.23	7377958.78	7899744.14	10344143
L-ascorbate	393170.33	422174.053	492800.016	565809.257	461260.199	505595.52	482408.203	295141.335	351892.422	1036322.52	348593.986	334765.952
glycine	3495125.86	3111909	3216985.93	3008303	3397380.51	3540832.83	2540908.58	2646096.41	2483863.59	3180417.38	3509471.67	5527334.84
succinate(2-)	10080860.1	10289107.8	9704497.43	8136586.26	8922022.18	10801257	8313352.17	7767346.14	8391435.38	7832492.44	7295505.84	8859583.75
Dihydroxyacetone phospha	425824.727	448719.046	384630.945	378921.222	414831.107	385908.214	549517.098	640539.441	501454.944	273456.495	448812.748	346726.493
Phosphoenolpyruvate	91557.7837	105562.495	76776.9065	107521.766	96760.6749	106785.098	329759.098	238690.726	240291.746	66921.5352	137544.39	44626.5652
IMP	167624.758	137464.05	98541.6241	117733.139	105538.312	157960.346	140194.176	113699.486	113960.844	158863.535	119643.669	145466.064
inosine	1430.79415	2022.853	2494.3676	1758.1109	2007.126	1254.196	1852.6703	2258.0995	2025.0301	1166.6335	2105.0702	1869.14305
