# Supplementary material for: Glucose transporter 1-mediated glucose uptake is limiting for B-cell acute lymphoblastic leukemia anabolic metabolism and resistance to apoptosis
Source: Cell Death Dis. 2014 Oct 16;5(10):e1470–. doi: 10.1038/cddis.2014.431 (PMC4237255; doi:10.1038/cddis.2014.431)
Supplement: Supplementary Table S2 [file cddis2014431x4.pdf]

Table 2. Results from 13C-glucose tracing in Glut1fl/fl B-ALL cells +/- Tamoxifen

Cells	WT	WT	WT	WT	WT	WT	Glut1fl/fl	Glut1fl/fl	Glut1fl/fl	Glut1fl/fl	Glut1fl/fl	Glut1fl/fl
Treatment	Vehicle	Vehicle	Vehicle	4-OHT	4-OHT	4-OHT	Vehicle	Vehicle	Vehicle	4-OHT	4-OHT	4-OHT
Glucose-neg	306828.2365	331239.9595	256185.7185	434036.2333	501387.2387	402248.2452	389194.6826	293820.174	365227.768	588471.2991	612962.0553	556740.7603
1[13C]Glucose-neg	15893.5916	17164.2815	14839.38105	23610.0602	25225.214	23846.65915	19743.1127	14986.9474	15333.92505	26791.6577	25592.8398	22710.69735
2[13C]Glucose-neg	0	203.90955	73.47815	0	0	0	0	0	0	0	0	1399.74705
3[13C]Glucose-neg	428.7621	0	0	0	0	0	0	0	0	0	0	0
4[13C]Glucose-neg	4231.07345	6847.84915	4697.0452	4889.479	5276.13165	2103.3581	5659.5775	3848.58105	5165.72415	10791.7141	8772.04575	15815.8162
5[13C]Glucose-neg	234729.8015	259289.994	192719.4888	232092.8625	268814.2546	223079.5142	335522.1547	251391.9458	341580.0268	495087.7913	504607.7867	503893.9422
6[13C]Glucose-neg	5405271.738	5763696.057	4322383.655	5246021.833	6257964.001	5081279.864	7510679.054	5575400.917	7353211.027	10982098.8	11437968.18	10834326.55
hexose-phosphate	86121.45555	110159.2609	78703.59625	90368.3746	92065.9966	125467.1553	83513.39165	70356.97445	95225.88415	61179.8329	58419.16635	58494.73495
1[13C]hexose-	4946.54135	5441.20145	4554.974	5102.6563	5838.2054	6422.65785	3043.8406	2438.05175	3227.76575	3900.99915	4128.8368	3900.28185
2[13C]hexose-	732.3926	1168.28895	1261.4487	3751.25725	3639.80685	3128.7096	1613.2779	1419.8708	1454.7714	2042.74175	2141.985	3775.9606
3[13C]hexose-	22295.81365	33372.5738	21514.09865	36827.14575	34309.96625	36192.6473	35399.15285	31716.8963	35074.4316	20757.13505	15000.23365	22912.1832
4[13C]hexose-	5040.73785	7073.01425	3569.69145	7345.755	5920.7484	6825.632	4766.2092	4770.2382	3914.0828	1372.4841	558.92045	2258.07595
5[13C]hexose-	25402.2264	31810.386	24778.74275	30761.33295	27033.3414	31621.354	40944.9879	34320.76165	38604.95425	9186.7236	9013.16965	9948.4355
6[13C]hexose-	405981.0496	505059.4995	438832.6734	400369.279	363845.0271	509485.4913	727985.4521	577547.5586	681039.8071	151917.0301	130878.7992	171748.3199
fructose-16-	38675.64585	27316.56445	19458.4082	20106.16135	19576.26525	29575.5014	37310.95885	41256.85015	55164.0087	25626.0127	32447.31335	36937.2319
1[13C]fructose-16-	566.7311	532.617	76.3875	166.8939	159.8988	198.34085	303.26865	611.4068	906.59825	0	0	62.0235
2[13C]fructose-16-	275.7295	323.8164	411.10165	335.2554	311.67935	353.5276	400.05775	522.01445	638.3603	776.047	998.039	599.7619
3[13C]fructose-16-	19349.20785	18592.7578	16058.1553	17493.13255	16902.92985	19302.87445	24262.829	25615.05305	25384.3604	26347.86285	23941.33395	26746.61555
4[13C]fructose-16-	4501.7004	4375.8857	2950.0518	4502.23625	3856.1942	4908.34515	5383.3813	6198.98455	6444.26385	4170.527	7750.5254	4004.0674
5[13C]fructose-16-	24464.1225	22522.7793	16741.67055	19576.24265	20474.88	24936.5376	32105.8542	32715.42985	33907.2027	32808.0364	35455.88725	35883.6291
6[13C]fructose-16-	368278.8958	321793.7588	257345.4286	295521.1132	313238.558	384655.4593	461431.3809	455220.4077	489626.5638	475668.4864	535194.2456	538923.9969
dihydroxy-acetone-	28589.9859	30413.99475	22426.3427	23279.27855	20341.92065	33165.12965	21351.2604	21094.4794	24189.16315	10625.4547	8672.43475	9918.78955
1[13C]dihydroxy-	100.9593	0	0	0	151.734	0	0	0	0	0	0	0
2[13C]dihydroxy-	667.55145	159.88855	474.4577	1399.95835	1128.14655	860.7688	1162.604	412.15225	1084.94015	2659.35975	1849.04335	1211.1296
3[13C]dihydroxy-	39182.0812	41473.0501	43984.9539	28181.02865	20928.0216	40472.26745	64657.95235	42488.3341	53513.12845	2298.00765	2678.8685	7594.28255
3-phosphoglycerate	11538.72195	8623.71895	8394.5148	11864.023	7459.17705	14695.01785	5528.41955	5350.9813	10541.48395	13154.5663	10250.73205	8543.10705
1[13C]3-	0	0	164.0531	292.8657	0	0	120.8844	0	153.05	0	0	0
2[13C]3-	752.96665	616.6128	509.4062	325.6298	620.4536	586.14465	292.77735	143.482	880.72025	0	0	0
3[13C]3-	99573.52555	86270.32635	76489.2738	81424.84455	63894.8879	107733.1497	72917.8365	74068.6562	108140.7374	16519.9332	18427.0529	22154.60135
Phosphoenolpyruva	9352.1945	9056.04145	8351.9647	8608.3205	6834.4073	11380.7509	4267.73605	5264.54375	6933.9114	10801.9707	8557.4168	5562.9645
1[13C]Phosphoenol	0	0	107.566	0	0	0	0	0	0	0	0	0
2[13C]Phosphoenol	2880.0542	2424.8668	2515.15185	1948.6377	3162.9305	3024.58405	1060.1767	3290.2536	2480.51355	843.80435	455.731	486.3282
3[13C]Phosphoenol	45761.01655	35337.58425	41991.0307	35001.3191	27187.74945	45905.91005	46743.47725	56224.01785	86693.669	6034.0214	5396.49985	6991.1584
pyruvate	325610.8478	366918.3656	240683.4384	468479.4487	589496.7909	388220.9313	406457.6251	379929.0086	440670.9791	629498.2979	530116.027	734127.8615
1[13C]pyruvate	11897.6784	13545.76745	8494.76565	16340.90045	21812.30485	13501.2945	15696.3165	12293.69005	16919.13875	22045.2022	16837.9265	25131.6443
2[13C]pyruvate	4280.7737	5408.50105	3966.81155	4385.2138	5932.62875	2561.1128	6507.34615	1051.58185	9724.68015	1348.3234	324.1453	1568.66335
3[13C]pyruvate	133168.5586	161840.8787	116109.3836	142269.4038	195920.2182	94187.4261	209686.9566	251444.4581	314542.5547	48042.4671	23290.56705	57292.88775
lactate	36040474.92	41972786.93	25881792.88	51081886.31	60910211.79	52334006.09	41616659.84	36975441.7	47413763.87	70104947.93	73080318.12	65855509.29
1[13C]lactate	1416414.573	1650325.002	1064318.869	1903325.847	2270733.285	1991100.518	1561140.439	1427749.133	1816356.807	2428738.216	2530471.04	2269323.115
2[13C]lactate	793144.1837	916668.3011	760902.5644	745210.263	801472.6173	918165.6807	715248.9913	745426.1893	872758.114	128811.0131	119787.3945	123515.4145
3[13C]lactate	25081293.38	29025802.22	24110974.79	22840645.7	24421201.81	28366277.17	23427092.25	23971158.7	27887530.19	2315373.284	2301699.417	2358611.039
Glycine	898488.1525	980957.2431	877130.6562	883797.2334	798955.2791	1156921.84	847903.0166	822198.5488	794547.2249	807669.931	784341.9848	815011.8097
1[13C]Glycine	16685.73165	17263.9381	22882.7346	18375.95885	16083.019	23950.26055	12518.9395	12348.86765	11066.87285	5602.3161	3560.36535	4999.92835
2[13C]Glycine	52985.69405	54034.9335	61385.9922	39110.4449	33647.24535	58042.33195	28282.73195	28775.0593	30944.78465	866.096	635.427	826.4769
sn-glycerol-3-	365463.7067	563874.1403	328726.977	515176.8636	502915.1213	741741.899	318039.433	298179.4499	328141.3406	355555.7048	315869.3347	325207.6099
1[13C]sn-glycerol-3-	24133.18995	35120.09	21611.1289	28781.56245	28330.38905	44039.93565	18175.41155	18065.60785	21466.0284	16954.90015	13520.9992	14121.73065
2[13C]sn-glycerol-3-	53999.9757	75398.46775	47362.234	61136.05215	55043.03335	83192.6562	45962.9666	44924.02825	44106.162	16930.64755	14237.3794	16934.5112
3[13C]sn-glycerol-3-	1546867.825	2186440.357	1411378.762	1619768.811	1467801.836	2321089.776	1409571.805	1244443.534	1364558.007	308204.4826	281222.253	339975.5744
fumarate	852284.5733	996638.5517	814478.6246	904912.677	857321.7411	1032435.078	857991.1273	726069.0587	848631.9808	587140.6908	558220.1055	549347.4269
1[13C]fumarate	75833.0007	92765.2364	80425.9823	74858.65105	70038.1001	88841.58275	81799.9441	68876.35605	74374.65305	26985.36655	24720.3706	25620.24915
2[13C]fumarate	77828.6467	90766.70755	86992.3644	62867.46115	60567.92825	80982.1946	66527.9427	58295.8802	57490.56535	4163.8649	4275.37355	4618.1667
3[13C]fumarate	52909.42915	56609.1422	57353.0213	50363.42785	41430.05785	58421.8642	48718.834	45767.27725	48669.0226	6082.3675	3675.7227	5089.919
4[13C]fumarate	25095.00965	20702.22995	28691.8918	24978.38975	22058.7822	21962.5634	30592.81405	25806.99935	26379.2598	14511.1914	15449.85715	26254.87175
succinate	5032842.932	6114665.741	4353181.707	6191546.543	6643958.117	6643669.975	5344594.24	4730303.43	5514903.264	7185489.138	7314072.48	6695944.45
1[13C]succinate	384756.4129	484435.6449	353085.1765	414298.7478	426781.7541	489323.1753	375808.3506	339093.9272	381178.8592	342191.6613	340546.1037	304862.0773
2[13C]succinate	355288.703	459155.2204	385649.7122	293619.3833	260948.6832	383660.5158	246106.9004	232103.2438	235248.3974	22234.58135	18540.90975	18776.9234
3[13C]succinate	97613.215	113832.6594	112211.3663	75386.4837	58749.0514	110119.0635	70299.22865	64200.1802	69698.1742	3088.79095	1908.036	1640.1591
4[13C]succinate	12054.05195	12910.55775	18305.1462	10302.78565	7419.2905	12220.49065	6020.981	6573.0152	6241.8847	4173.2837	3814.38435	3732.99925
Pyroglutamic acid	1295579.045	1584854.919	1238921.27	1311832.962	1252606.728	1698777.137	1374592.382	1265239.497	1433635.091	1177282.876	1122992.433	1074251.5
1[13C]Pyroglutamic	112183.0737	142181.0527	109179.1966	111883.1798	108142.2091	142408.031	121294.9772	105747.2446	119810.1958	75562.95535	73582.87955	64571.89045
2[13C]Pyroglutamic	143632.6476	181302.9084	156771.1203	114956.1808	103782.4117	156875.1319	117846.1908	104795.6635	113336.8744	9042.3562	4164.8456	0
3[13C]Pyroglutamic	22817.90815	29731.09065	26984.1566	19830.78445	18904.073	23645.9946	21719.8173	19313.5991	20179.38045	9848.0941	9301.76735	7804.1032
4[13C]Pyroglutamic	7652.27385	13970.273	12687.1694	7404.57705	5957.6725	3840.97565	6910.3788	6641.25225	5266.7226	2340.2925	0	1300.7628
5[13C]Pyroglutamic	1890.94885	1747.4053	1527.3125	203.08665	860.3556	1542.0951	1450.7141	1302.7481	796.6546	259.12575	139.319	140.803
malate	11800909.45	13597293.08	11220210.34	12215648.47	11755654.93	13883027.89	11569484.65	10331548.08	11448650.13	8220065.371	7758833.105	7497530.553
1[13C]malate	1150237.18	1342265.776	1124135.663	1109879.607	1024603.121	1277362.776	1181158.306	1025546.246	1098497.468	444823.1287	426615.3147	429314.6536
2[13C]malate	1173229.214	1357405.231	1262836.008	962907.4669	869877.7184	1132470.547	967989.7838	852186.0945	884672.7326	113612.6488	109911.937	140562.4322
3[13C]malate	830292.2248	898992.776	884182.1459	792538.5922	674013.0891	908602.8448	744344.0063	698017.3656	776165.544	131611.7486	118971.0774	141913.3907
4[13C]malate	41812.33475	42786.50625	50157.4479	28636.25475	24823.04505	34248.7287	32151.96265	27514.10375	27529.0776	797.34915	1285.4127	2847.75085
a-ketoglutarate	338362.4015	277893.2615	406742.5447	253658.9596	284174.8505	284877.2193	391098.1012	73516.2868	459637.003	340439.9636	167246.3878	343904.1879
1[13C]a-	24390.1687	18366.0243	30825.53155	12448.87405	9554.8271	18421.4954	29521.9674	451.4874	31147.64365	12072.31955	9987.5124	12232.2066
2[13C]a-	23939.5591	12567.6539	37206.59055	4708.81105	5171.68405	11120.06945	28377.8525	920.9471	26133.5343	0	0	466.2334
3[13C]a-	992.29845	0	2817.4271	0	0	0	0	0	0	0	0	596.0721
4[13C]a-	1339.5152	703.8837	315.87075	0	0	0	0	0	0	0	0	0
5[13C]a-	0	0	0	0	0	0	0	0	0	0	0	0
citrate-isocitrate	8984421.46	9628139.114	7372188.194	11035293.53	12542481.42	11657120.3	8320352.8	9228852.044	10228705.44	16134297.48	14161917.62	7902123.959
1[13C]citrate-	739638.4372	774463.3479	610072.5789	864733.3779	958340.9078	924285.536	724528.8224	805033.5852	858388.3262	1210789.893	1075137.269	607514.1437
2[13C]citrate-	1197546.491	1214925.006	1159414.093	964720.6468	953577.118	1188625.618	1425418.329	1646196.015	1620474.247	1166253.913	1099070.116	719708.0221
3[13C]citrate-	174986.4305	170399.2874	159769.7785	135977.5641	132832.1269	176484.9583	227630.2028	255795.7183	234464.1182	169458.444	153400.9521	97754.78685
4[13C]citrate-	259716.1317	258214.6402	260881.5729	202726.8727	204465.9722	233161.3169	282115.5416	342203.593	355804.3697	269847.2034	260966.6785	170718.8374
5[13C]citrate-	97959.4921	101818.9454	99744.14535	78744.23155	81363.50465	91920.4464	110989.1909	135560.877	135951.0118	109540.3163	102748.326	68000.96615
6[13C]citrate-	11232.7572	11368.46385	11373.60855	8696.0709	8493.9514	7903.1473	11822.35635	15249.6129	16798.0945	12399.9942	11985.47215	4857.19685
2-dehydro-D-	33968.7931	46117.08135	27895.75665	52891.09365	71325.7823	50253.21245	42653.8796	40673.59305	42822.0684	84629.8137	93932.58455	83900.46475
1[13C]2-dehydro-D-	918.75905	1013.0132	791.9847	2462.8695	1795.47325	838.5975	1170.702	429.5523	687.0463	2036.71455	3077.59395	3284.2529
2[13C]2-dehydro-D-	1469.79845	2113.086	2387.8356	2735.2628	1537.148	2968.19915	4328.76	4639.1453	5724.3611	8340.08565	8948.90295	7655.3264
3[13C]2-dehydro-D-	0	0	0	0	0	0	0	0	0	0	0	0
4[13C]2-dehydro-D-	0	0	0	0	0	0	0	0	0	0	0	0
5[13C]2-dehydro-D-	0	0	131.03805	536.98125	0	0	128.85795	0	0	0	0	250.7427
6[13C]2-dehydro-D-	4483.2972	7989.7758	4212.5306	4245.238	3555.93465	3819.92655	7847.64425	7561.53585	9570.97875	9289.75405	9006.25005	8465.7569
D-gluconate	608843.6659	817421.6059	480755.7106	861345.2628	1096024.484	842038.2897	1707940.322	1645334.498	1883783.416	2608266.244	2579976.194	2511075.298

1[13C]D-gluconate	38147.56895	48024.4845	30062.7109	52649.45845	68309.4486	52941.5416	117539.9119	124936.5662	136542.7952	273912.8323	259406.4316	246066.7847
2[13C]D-gluconate	1062.43635	2896.73785	1683.15345	2355.70745	883.91115	3440.97285	23357.17055	29974.6724	37226.12365	169724.9259	134641.0317	141130.3687
3[13C]D-gluconate	22137.31115	31709.94265	16605.0456	19311.71865	20507.05955	32170.41065	165039.9534	178273.5286	208430.1718	326994.1798	296491.6265	294028.7714
4[13C]D-gluconate	4259.86905	6267.30265	1819.0526	1591.66185	2857.8099	6282.0095	38736.7072	42985.8214	54101.7514	51572.1998	45228.5384	49880.0105
5[13C]D-gluconate	24553.8872	29163.84665	19270.113	25163.26765	26382.0351	33916.4447	154298.1722	154334.4656	185052.8603	123070.8701	112638.1921	108579.1825
6[13C]D-gluconate	354259.7672	480148.9142	342962.2373	380592.3363	383393.8388	470272.9136	2113163.358	1950052.531	2249164.233	997219.6825	928447.7929	933998.8757
ribose-phosphate	655.27095	1054.37415	725.626	516.22325	765.3684	1150.6097	1516.9358	1326.91815	1024.0134	1097.87145	1837.8888	1219.37315
1[13C]ribose-	121.1769	0	0	0	0	0	0	179.834	0	150.55755	0	0
2[13C]ribose-	2378.8465	3508.50775	2159.492	1572.0119	1249.0337	3008.9636	2554.68415	0	1850.5791	683.37555	839.31575	790.66445
3[13C]ribose-	679.64135	309.61515	161.756	398.08815	655.515	989.0946	2190.72135	493.638	861.44245	0	0	0
4[13C]ribose-	247.87035	911.70495	420.91935	1101.3437	246.55215	0	995.58565	805.58375	1543.89845	0	0	0
5[13C]ribose-	33801.6045	36713.7023	28000.27195	24982.9444	20026.2008	37582.18415	40906.44325	27431.38195	45356.03765	2195.6142	2100.2929	4576.839
thymidine	20124.7536	28174.5728	11591.61115	36987.02305	40043.7372	39013.22525	45789.03995	36322.9033	53874.5632	78631.2964	86459.6342	81388.4156
1[13C]thymidine	3742.76375	4915.5175	2489.3599	5778.88835	5991.06455	6224.681	7448.95075	5189.4677	8461.46035	8771.851	9622.8774	9298.38075
2[13C]thymidine	5688.92	4069.2785	3008.94315	5096.6706	3663.52215	9962.7413	3482.9543	37342.5228	3530.9149	5108.1985	30404.74605	4107.7299
3[13C]thymidine	147.99925	401.4535	261.16225	82.442	0	617.6877	0	4178.00765	0	0	2921.5484	105.8989
4[13C]thymidine	0	0	0	0	0	0	0	0	0	0	17866.73325	0
5[13C]thymidine	3223.84025	4481.96675	5240.4409	98.95375	72.4332	869.76315	2305.77505	756.0308	1343.66775	0	534.918	0
6[13C]thymidine	98.1095	297.13345	1152.4157	45.0618	0	0	140.5128	0	53.2959	0	0	0
7[13C]thymidine	135.4831	134.4021	136.0917	0	79.456	0	0	0	0	0	0	0
8[13C]thymidine	0	0	39.0915	0	0	0	0	0	0	0	0	0
9[13C]thymidine	0	0	0	0	0	0	0	0	0	0	0	0
10[13C]thymidine	0	0	0	0	0	0	0	0	0	0	71.1924	0
uridine	1481385.011	1818491.152	648175.6715	2628845.228	2167232.186	3350973.083	2428603.671	2396980.812	2817365.86	1612772.424	1590933.543	1394301.043
1[13C]uridine	162539.174	199500.3459	81145.81635	273537.8329	223077.8123	349284.2377	246475.903	245480.7898	285722.2516	165263.2278	161491.7945	140219.2408
2[13C]uridine	113443.8706	149854.6398	153365.5488	51648.0742	41667.86995	72878.55045	46250.62165	63837.39345	44437.22475	22918.15845	29773.8565	16927.14375
3[13C]uridine	43723.2125	56336.63295	68077.533	14418.8907	11628.49585	22111.6328	14901.3739	11854.434	10068.0852	893.1151	882.403	812.99215
4[13C]uridine	58466.8612	73625.61575	85780.59975	17226.5424	13241.6164	28022.42035	19616.6188	13556.229	13699.54925	717.50375	199.97895	548.4353
5[13C]uridine	632300.8382	799323.3756	878776.9045	179572.5755	150541.5747	269040.1194	268824.7521	207093.5042	212978.8778	17152.1208	13768.16915	14990.62075
6[13C]uridine	76579.4421	96976.29095	116319.1123	18700.05165	15167.602	29168.07635	27131.7668	19822.94475	19390.1364	499.99385	0	115.52955
7[13C]uridine	37837.19085	48970.8739	62589.25185	8974.83665	6340.99665	13339.7926	12998.2314	10739.18495	10851.32195	0	156.368	0
8[13C]uridine	7174.9962	8029.3503	12964.0212	544.33265	170.69425	2129.78875	1377.28795	2557.40045	902.035	597.75965	380.59	256.3566
9[13C]uridine	10370.06605	11592.59125	3763.4155	11595.62535	8701.8239	16510.0083	16012.2465	18284.2437	21353.00025	10127.6855	10303.5074	9547.25015
6-phospho-D-	3405.2754	1483.5294	1253.6863	1230.1579	1283.70615	2924.8796	10460.81885	8565.67685	15654.1355	6212.57685	4994.5192	8455.73725
1[13C]6-phospho-D-	0	0	0	0	188.436	0	147.20745	0	0	0	0	0
2[13C]6-phospho-D-	365.48	0	0	555.28745	848.3716	349.72275	356.21215	0	396.60465	1841.9334	2342.586	1475.00665
3[13C]6-phospho-D-	382.94285	277.3836	156.998	388.9306	207.244	1686.19975	862.7339	906.0308	615.2599	1644.71925	1874.44765	1589.97455
4[13C]6-phospho-D-	0	0	0	209.48655	0	0	0	0	0	0	0	0
5[13C]6-phospho-D-	2469.11365	1821.794	1282.10595	1461.55755	1545.7322	3155.22445	2765.8516	1846.0739	1742.5295	692.45675	758.98345	1366.27455
6[13C]6-phospho-D-	23085.9652	21449.7767	14346.8986	17618.8665	15183.6386	26082.3029	34449.1287	35445.1037	30437.2002	14150.19885	14366.0591	16286.9635
D-sedoheptulose-7-	4564.30155	6005.6673	4272.493	4614.4104	4686.11905	7059.55895	6323.80495	7352.3747	8691.3694	1058.45955	503.63565	622.08825
1[13C]D-	191.32	513.9265	0	356.50655	229.314	194.68	175.816	152.87	256.31775	140.4	172.49115	0
2[13C]D-	786.6052	1393.8172	104.308	1446.8473	1827.3239	1061.75895	3250.40255	1233.7401	3149.97595	5185.1386	5858.0531	5339.6771
3[13C]D-sedoheptulose-7-phosphate	1389.95455	2052.997	2034.187	2999.93915	1438.83	4626.39225	1727.6951	1662.12245	2008.64545	651.55135	398.14075	494.96655
4[13C]D-sedoheptulose-7-phosphate	6452.61035	8164.9936	4585.6066	7058.2373	6439.19385	10388.857	9780.0902	9646.4412	10886.88655	2345.48465	2119.06805	4470.2638
5[13C]D-sedoheptulose-7-phosphate	2471.8661	2900.26875	1612.256	3641.81745	3527.9297	4559.69155	2831.56475	1981.0858	2006.69045	692.8557	509.60325	418.90965
6[13C]D-sedoheptulose-7-phosphate	5311.8981	5748.0144	4315.786	5820.71275	5863.48375	8461.9119	5852.0481	5471.53095	5679.32665	965.3967	947.58965	818.40175
7[13C]D-sedoheptulose-7-phosphate	53677.66725	60231.59235	45082.888	48483.03855	46453.46105	71401.7178	74389.52635	68878.16475	76403.067	11005.3216	9038.50915	12279.469
2-hydroxygluterate	137395.3848	142269.1267	141465.836	142106.0196	155817.1987	145948.5374	154018.565	224324.8775	141430.0931	213177.6841	318730.3758	210004.0665
1[13C]2-hydroxygluterate	9602.98595	8953.08875	10401.04145	8671.0602	8920.6505	7728.6821	9314.1443	12054.44835	8045.7651	12916.51835	16572.58905	11782.93895
2[13C]2-hydroxygluterate	8003.46715	6633.2547	8935.66175	3846.2685	4290.30925	4227.3796	5176.54805	3717.65045	4572.5215	0	0	232.72275
3[13C]2-hydroxygluterate	509.6593	689.97015	832.3188	281.5059	166.036	0	167.2983	198.51	0	0	0	0
4[13C]2-hydroxygluterate	127.08	0	349.8079	0	0	0	0	0	0	0	0	0
5[13C]2-hydroxygluterate	936.48745	454.99805	762.5735	341.15295	869.11695	123.346	144.1011	0	0	0	360.31125	0
S-adenosyl-L-methio	36457.11965	47068.1161	33879.00135	39339.87535	35696.3006	47724.49815	34844.6582	45144.75195	50397.74405	39585.4799	26956.2713	35250.9531
1[13C]S-adenosyl-L-	11360.92855	15715.88	10234.45865	14699.07345	10500.0562	17469.57265	7758.765	10370.72585	10468.01945	13357.27145	10468.95325	11881.5233
2[13C]S-adenosyl-L-	62076.12345	82208.4217	56269.87565	63050.0719	51370.5183	77276.65345	43601.40025	40705.3608	40768.0259	40734.2977	28021.7294	31744.08675
3[13C]S-adenosyl-L-	25350.2549	32908.68725	23489.50375	30091.4037	22419.70975	35988.2592	19286.93855	20039.67705	19712.1561	7008.87225	4732.02255	5769.0449
4[13C]S-adenosyl-L-	32794.28545	42295.0328	31745.9781	34748.6563	27095.4567	40716.911	24813.53965	24283.89505	24610.78555	5727.614	2920.0434	3720.53065
5[13C]S-adenosyl-L-	397149.417	517749.7687	377437.0583	363285.2575	288651.9865	432348.2344	336655.0532	335542.5987	334869.9554	65738.5613	47811.6224	62015.61365
6[13C]S-adenosyl-L-	46169.1072	58808.8469	52475.91665	43040.33585	32274.97105	48913.26915	34099.3795	34693.149	35342.41365	3010.47135	2927.39945	3463.3554
7[13C]S-adenosyl-L-	2145.1091	3315.2747	6408.38	1530.22985	727.5096	2298.70395	570.9569	681.27665	295.04	0	0	129.3615
8[13C]S-adenosyl-L-	0	311.1957	736.07155	40.4201	51.1704	96.387	0	0	0	0	0	0
9[13C]S-adenosyl-L-	4651.6702	6649.86335	6834.87965	2494.3023	1069.87275	3757.67535	1274.81125	1247.3901	662.8032	138.0264	174.3465	45.7539
10[13C]S-adenosyl-L	112.88425	152.98435	282.343	105.9078	0	293.5757	645.17765	72.6846	79.9595	104.2797	0	116.51625
11[13C]S-adenosyl-L	0	0	0	0	0	0	0	0	0	0	0	0
12[13C]S-adenosyl-L	0	0	0	0	0	0	0	0	0	0	0	0
13[13C]S-adenosyl-L	0	29.53	0	0	0	0	0	0	0	0	0	0
14[13C]S-adenosyl-L	0	0	0	0	0	956.1655	0	0	0	0	0	0
15[13C]S-adenosyl-L	0	0	0	0	0	0	0	0	0	0	0	0
S-Adenosyl-L-homoc	4526.79845	5035.36405	3864.05185	4652.56315	2610.3145	4006.4088	558.038	0	1579.19285	4196.1983	4283.18575	2325.5395
1[13C]S-Adenosyl-L-	494.464	887.384	514.7172	359.2953	655.478	0	0	0	0	361.78545	0	462.218
2[13C]S-Adenosyl-L-	8404.03835	10652.809	6022.85785	5147.36685	5035.61135	6862.05725	0	0	526.93485	1279.08715	1577.0196	1211.2503
3[13C]S-Adenosyl-L-	830.444	2807.6217	2192.99505	2628.12775	0	2573.19445	0	0	0	0	0	0
4[13C]S-Adenosyl-L-	1642.614	2658.17185	2537.4923	2384.16865	1653.23	751.1946	0	0	0	0	0	0
5[13C]S-Adenosyl-L-	41818.3758	65646.9068	52300.2035	37771.35435	35227.1374	39171.3304	20167.35435	18758.86615	25168.3438	2665.9652	911.98575	1360.5683
6[13C]S-Adenosyl-L-	4553.596	7029.71045	6507.05565	7548.0547	8993.48765	2959.0566	6398.07825	4400.602	1866.64725	19447.9351	13503.22105	13363.4455
7[13C]S-Adenosyl-L-	0	0	0	0	0	0	0	0	0	0	0	0
8[13C]S-Adenosyl-L-	0	0	0	0	0	0	0	0	0	0	0	0
9[13C]S-Adenosyl-L-	0	0	154.6766	625.32795	0	0	0	0	0	0	918.4734	0
10[13C]S-Adenosyl-L	0	0	0	0	0	0	0	0	0	0	0	0
11[13C]S-Adenosyl-L	0	0	0	0	0	0	0	0	0	0	0	0
12[13C]S-Adenosyl-L	0	0	0	0	0	0	0	0	0	0	0	0
13[13C]S-Adenosyl-L	5230.08735	6827.80495	2026.40335	3248.299	3939.05455	4224.98715	421.886	115.476	0	1377.50475	574.861	0

14[13C]S-Adenosyl-l	86.1021	91.536	0	264.156	0	0	514.252	370.384	0	0	0	0
dGTP/ATP	371738.2223	264215.9392	195313.97	209441.6784	219960.5613	375250.5398	517630.4497	816832.817	831226.2364	478711.4844	625298.7837	590588.3629
1[13C]dGTP/ATP	108476.5469	70342.69275	54527.5716	57867.7374	61017.17555	110142.7943	99993.6599	154504.5873	133578.7748	164304.1932	199481.8411	179941.443
2[13C]dGTP/ATP	561903.0206	317717.0695	229742.3845	300577.1424	305263.0848	538842.6259	430523.9214	547806.6282	405220.0654	392790.0275	506054.2677	469256.0085
3[13C]dGTP/ATP	212661.1245	123185.8084	84323.97635	125273.3358	132973.726	232058.5296	167623.8031	234951.3682	172708.5638	88435.54025	98896.0731	114476.5228
4[13C]dGTP/ATP	293820.6588	161524.1646	107426.637	163943.9007	163018.5847	294217.2237	237832.4496	322196.0612	224240.7944	95148.8267	113650.7148	120774.1331
5[13C]dGTP/ATP	3540865.175	1949406.995	1321404.855	1688471.824	1705686.718	3097118.146	3094532.689	4053708.132	2898844.114	658807.237	898805.4291	1063521.931
6[13C]dGTP/ATP	257957.7541	144116.7163	114497.1935	116119.6496	119649.1036	221934.7627	213585.2217	267826.5424	189439.7931	43596.54725	54072.9096	65791.88125
7[13C]dGTP/ATP	103873.9621	56063.15765	43878.6781	44355.6496	48681.1694	92489.36545	90478.6963	115968.3648	81776.0334	9450.1906	16612.01285	24885.05165
8[13C]dGTP/ATP	1588.9973	524.4802	862.55975	408.4015	382.296	470.92995	870.9106	421.94445	0	0	0	0
9[13C]dGTP/ATP	0	0	0	0	0	0	0	0	0	0	0	0
10[13C]dGTP/ATP	0	0	0	0	0	0	0	0	0	0	0	0
ADP	294094.2764	265331.0873	167350.8848	245824.2223	270719.3302	395488.9225	358880.3078	470155.8089	522206.685	679416.8913	644499.4644	739197.0061
1[13C]C10H15N5O1	91390.09765	76355.78795	48910.55145	79665.8612	86405.1673	127247.5009	77088.1245	88143.39495	94452.48505	229959.3628	223790.7841	249920.2728
2[13C]C10H15N5O1	529014.2887	452776.0799	241668.1477	398610.8502	400648.0228	658980.5349	417803.3731	443855.2907	433148.2518	604634.6012	587170.9489	666671.5688
3[13C]C10H15N5O1	198770.0559	166598.8332	99679.39305	174447.9281	168463.2427	288548.8516	175041.4192	195348.514	185030.0924	121033.5318	123036.1054	146769.56
4[13C]C10H15N5O1	271732.033	234804.283	132796.3412	215206.3712	232960.0224	350970.8729	257638.217	257615.1917	244439.2138	127073.4836	128909.5787	161009.6872
5[13C]C10H15N5O1	3352147.482	2802662.309	1560414.58	2226947.153	2370230.79	3661005.666	3173995.138	3339412.216	3193947.111	940898.7285	1034048.545	1349305.624
6[13C]C10H15N5O1	205206.0703	171242.6155	117852.2159	133487.2418	132775.6678	222843.2096	177746.4246	186509.796	188575.6715	42435.57135	45061.8964	61565.2841
7[13C]C10H15N5O1	64314.1225	55754.01835	39616.6337	42843.0567	45777.11845	74065.41065	65905.6334	66682.05945	58245.49065	12733.68365	14664.4634	18009.6966
8[13C]C10H15N5O1	1246.22345	0	283.65235	0	0	942.60035	0	0	0	0	0	0
9[13C]C10H15N5O1	0	0	0	0	0	0	0	0	0	0	0	0
10[13C]C10H15N5O	0	0	0	0	0	0	0	0	0	0	0	0
cytidine	174902.2021	196637.1536	149585.5699	238034.2341	287521.694	232575.7116	179134.492	142347.3912	169094.6996	290443.125	325596.9323	303804.2353
1[13C]cytidine	16505.8778	17546.5838	12888.91065	22593.6217	27292.8638	21499.39045	17771.04065	11755.9485	16519.2449	29989.46145	31867.52295	28465.6832
2[13C]cytidine	0	0	0	0	0	376.2324	271.536	0	0	0	0	0
3[13C]cytidine	0	0	0	0	0	0	0	0	0	0	0	0
4[13C]cytidine	0	0	0	0	0	0	0	0	0	0	0	0
5[13C]cytidine	5146.10595	5254.46095	8294.2885	6840.863	8772.6137	5333.4778	7597.2496	2276.73965	3589.0429	5674.3959	705.90975	4483.9665
6[13C]cytidine	0	0	0	0	0	0	0	0	0	0	0	0
7[13C]cytidine	0	421.704	962.15525	0	0	0	0	0	0	0	0	0
8[13C]cytidine	857.242	0	2158.15095	0	1181.972	0	1384.718	0	0	0	0	0
9[13C]cytidine	0	0	0	0	0	0	0	0	0	0	0	0
2-deoxycytidine	5452.5383	10286.3016	0	10166.30705	28487.13905	7055.26895	66886.28195	21918.4412	68225.5262	173282.7859	133417.7513	147204.7461
1[13C]2-deoxycytidi	0	0	0	0	1175.65655	0	5136.81115	979.1866	4728.7543	13864.14485	8754.6904	12770.069
2[13C]2-deoxycytidi	0	0	0	0	0	0	0	0	86.35905	0	0	0
3[13C]2-deoxycytidi	0	0	0	0	0	0	0	0	0	0	0	103.87145
4[13C]2-deoxycytidi	0	0	0	0	0	0	0	0	73.82885	0	0	0
5[13C]2-deoxycytidi	274.53375	729.9252	334.6872	0	142.14295	89.8788	4399.38955	214.9383	2823.74625	201.1298	3555.4627	112.39535
6[13C]2-deoxycytidi	0	0	0	0	0	0	0	0	0	0	0	0
7[13C]2-deoxycytidi	0	0	0	0	0	0	0	0	0	0	0	0
8[13C]2-deoxycytidi	117.49275	534.44665	151.9078	815.73265	129.2434	1103.4765	694.57325	673.3503	277.9334	99.2628	1407.3045	976.25275
9[13C]2-deoxycytidi	0	0	0	0	0	0	0	0	0	0	0	0
Hypoxanthine	20298146.55	24433812.76	14110003.14	23649109.16	28094852.01	25934584.52	25270028.66	18417802.54	24834092.08	44179149.6	45010418.57	41731205.57
1[13C]Hypoxanthine	1195708.636	1442291.446	1028231.564	1332618.642	1603085.682	1475647.961	1404303.341	1012903.107	1361211.136	2396771.717	2435507.31	2263958.709
2[13C]Hypoxanthine	45565.36845	61403.9535	99799.53445	37995.45205	45822.352	48413.2944	33850.1944	23490.0132	28200.54985	46376.876	47555.4449	43523.2188
3[13C]Hypoxanthine	3119.22355	2765.2405	17443.743	734.776	0	432.14985	0	0	0	0	0	0
4[13C]Hypoxanthine	614.92845	912.6785	408.978	456.5725	1819.31785	968.2496	2058.0706	2163.4854	1729.1009	2015.9879	2933.8767	1390.8355
5[13C]Hypoxanthine	0	0	0	0	0	0	0	0	0	0	0	0
2-deoxyguanosine/a	13597.98715	426255.4015	355894.7656	306523.5225	826350.4762	26185.16615	678716.6435	563761.5509	1244460.702	339049.8246	12913.29715	420902.1029
1[13C]2-deoxyguanc	503.77515	153556.9369	117397.0457	111207.5251	310804.6275	4774.0638	169529.0598	122173.8689	273615.2241	125843.0293	863.4313	153496.44
2[13C]2-deoxyguanc	13796.97965	897752.0536	702080.0513	548903.1117	1483102.948	36282.08245	958869.1956	590913.2695	1276504.313	330760.0349	12213.4548	400762.3613
3[13C]2-deoxyguanc	4840.57855	296372.8443	275476.6806	209724.7933	550662.7358	18493.0706	405445.6947	282457.4166	550461.9625	55461.1042	6488.77725	73442.6258
4[13C]2-deoxyguanc	1409.82435	407908.4976	341499.1154	263338.2651	731563.8527	13804.12095	553114.1714	338514.2325	722956.5229	56995.3249	136.9687	74018.1656
5[13C]2-deoxyguanc	66509.1158	4769899.672	4043913.53	2608582.22	6840982.907	163197.8521	7129942.506	4521161.822	9468168.301	407994.4442	9337.5469	560636.7187
6[13C]2-deoxyguanc	1136.98595	279137.7401	313331.3345	139748.4061	388702.7863	4789.32705	391751.0094	234660.0078	529017.7426	16299.3178	0	23855.45715
7[13C]2-deoxyguanc	0	0	46371.37395	3348.4905	230.6045	210.886	388.4217	0	1418.5	253.8753	699.5424	139.24625
8[13C]2-deoxyguanc	0	121.40715	6042.7608	0	182.338	0	0	323.03015	104.5172	0	0	247.6113
9[13C]2-deoxyguanc	0	250.5264	0	0	0	0	0	0	0	126.898	0	0
10[13C]2-deoxyguar	1614.8703	806.3391	2918.2424	658.4268	301.57525	3356.5666	758.71925	6327.9043	588.98415	397.59435	3134.7771	511.99765
5-Methylthioadenosi	91182.09	118509.2517	76281.68065	92370.20695	76799.5068	108273.5876	40718.776	53856.4296	102990.1615	163551.6233	279018.7305	128387.9496
1[13C]5-Methylthioa	27041.25615	36254.56805	23808.5217	30099.96855	24641.2033	33583.4656	8642.54225	9322.71435	22921.71485	59611.4136	55276.97955	47935.4045
2[13C]5-Methylthioa	170396.5175	223509.0464	141239.554	148218.4499	123664.8165	174277.8629	54643.4709	59295.3341	115862.1446	165172.9536	108518.1044	142879.1802
3[13C]5-Methylthioa	61285.61365	80753.88985	56805.6432	61612.61195	55587.99555	73476.28575	24079.89735	29432.6603	54429.946	28443.9186	16349.82815	24973.00855
4[13C]5-Methylthioa	84899.98425	109436.7831	73561.48545	80937.41445	63883.25455	90005.883	31017.6592	31337.8887	68160.0244	27281.71675	13710.5063	25009.4198
5[13C]5-Methylthioa	1060819.457	1409634.629	943391.2813	842782.4809	692947.8847	991506.3959	453119.1312	490404.18	955637.7399	247148.0488	150680.7507	214402.8675
6[13C]5-Methylthioa	66435.0937	93637.00325	84717.0819	55792.1646	38922.3372	60094.56205	25599.62545	29114.5488	57484.5575	11035.00285	4843.47625	10906.8158
7[13C]5-Methylthioa	3322.0457	6076.56415	10695.78995	1273.254	385.42535	430.0042	0	1121.78265	0	174.43165	568.198	0
8[13C]5-Methylthioa	0	0	515.5562	0	0	0	0	0	0	0	0	0
9[13C]5-Methylthioa	0	443.0343	0	0	0	216.91665	211.89825	297.214	0	306.0404	0	0
10[13C]5-Methylthio	2528.2625	1617.5245	2409.10245	1545.20555	1341.96885	4020.3105	1581.4859	19464.81655	1203.01695	766.96085	5962.66705	987.7627
11[13C]5-Methylthio	0	113.9314	0	0	106.7518	183.4637	0	1437.43195	0	0	464.61575	0
UDP(3-)	410395.4044	337658.8026	176705.5765	576557.3511	564101.8261	769918.471	716585.9266	813605.7845	903801.1612	464297.407	460917.8847	507472.5264
1[13C]UDP(3-)	37943.8118	35264.9343	15739.47665	56955.03265	50863.80035	73529.97375	69111.3535	75639.61555	88357.5604	40418.79895	38691.09425	45006.8703
2[13C]UDP(3-)	33335.08875	27981.4904	32069.98085	18001.3908	15728.97435	25520.4631	21569.18185	25831.33285	23459.9792	10934.4645	13882.54775	13773.5825
3[13C]UDP(3-)	8240.4453	8416.143	12240.62305	567.2316	402.288	3395.24115	3258.39315	1974.56125	1548.54455	0	0	0
4[13C]UDP(3-)	13444.44685	7917.7675	18156.4686	697.17825	836.1243	3583.04825	2227.10155	2673.95905	1533.88755	0	0	0
5[13C]UDP(3-)	200712.5803	176146.9193	239836.0968	43738.0458	42785.0273	76230.90355	94991.4002	90270.7543	84656.0201	4304.5115	3401.2491	6705.03865
6[13C]UDP(3-)	18361.2498	17438.55975	26825.63165	1351.424	1507.55005	4664.4046	4951.3778	4861.0937	3708.3852	0	0	0
7[13C]UDP(3-)	8585.57685	8453.7127	17306.6687	361.228	0	1042.65205	1445.0082	2205.30655	1673.0149	0	0	0
8[13C]UDP(3-)	0	0	441.2343	0	0	0	0	512.9787	352.354	0	0	0
9[13C]UDP(3-)	739.986	0	0	510.932	0	211.8019	1353.75435	1769.7631	2020.78365	0	0	395.78
IMP	34996.49935	37249.6929	17060.7331	20481.4909	17625.90195	28767.9168	5846.80015	7454.9812	4258.45705	45584.34705	44061.0552	29595.61335
1[13C]IMP	13380.7619	14288.43055	4999.5704	5703.7115	6037.99095	13800.9715	875.8721	438.208	0	14205.80735	14292.26305	11416.92855
2[13C]IMP	101239.2503	106992.9205	54664.7559	51607.57655	50491.9874	70220.9568	12299.83175	12586.62835	5947.7903	48210.2126	52713.69935	40884.94825
3[13C]IMP	28231.88425	31421.6869	14863.77325	20115.09	14645.7665	22372.74675	4102.9825	3449.8665	1770.6414	7489.9746	6029.884	6832.1226
4[13C]IMP	32171.61285	30621.98815	12378.0911	16630.7802	20212.15155	26158.7032	0	0	0	5584.59805	4064.4239	2314.59345
5[13C]IMP	385714.8118	429686.6362	202711.9174	193270.736	173097.9067	279753.1592	90170.8101	100823.9267	69300.1638	54959.48535	51445.47465	43674.45755
6[13C]IMP	24174.07765	23090.74155	12465.03435	10732.05865	8307.82975	12701.84615	3140.88615	4491.34695	1299.6795	903.9141	879.2238	342.928
7[13C]IMP	22119.7088	23098.61635	23225.13495	16443.27205	12888.94445	16362.4522	33482.13965	64338.3008	72827.77965	19672.13935	24788.04545	21347.4642
8[13C]IMP	0	530.99085	815.34645	0	0	428.19075	2680.0522	4023.2459	7163.3321	448.79835	538.16	890.487
9[13C]IMP	0	0	0	0	0	0	263.684	985.832	0	0	0	399.548
10[13C]IMP	0	0	0	0	0	0	0	0	0	0	0	0
inosine	9136.53095	13773.58525	3602.56645	10759.7461	28396.03685	13576.5632	315503.8161	125741.8786	466595.0189	407039.6143	200363.716	292824.9771
1[13C]inosine	1041.41145	3317.98215	519.74545	1825.34075	7405.9988	3075.57405	82868.34015	28724.49585	103203.9332	151545.1559	27334.5515	109122.9622
2[13C]inosine	19272.17965	35850.5272	8655.6214	22139.2825	56147.40275	26505.9442	490300.1715	146097.9707	499035.392	418183.8448	48072.7328	308054.2086
3[13C]inosine	4702.76975	9383.48265	3016.59795	4725.29125	17126.75115	5805.9454	205642.8745	56741.2861	206932.8035	73244.3841	7427.4649	54501.8419
4[13C]inosine	7359.33805	14857.97605	3011.27545	8000.57885	23372.8461	10719.25655	279650.5692	78666.23235	276150.862	68774.3497	3958.0691	52064.15585
5[13C]inosine	103570.7594	170066.8026	45469.8787	94430.7889	237970.7174	125758.1666	3568826.756	1021046.757	3633399.763	503276.7597	51051.095	391889.6622

6[13C]inosine	16483.46325	16484.51805	59075.0076	6521.17675	14738.7519	24858.2621	201556.2574	76048.19115	206904.3506	52837.0639	9931.40215	32426.3914
7[13C]inosine	1001.7861	360.99	6293.27985	0	103.78845	1097.8887	495.77	1429.6296	0	556.5588	0	0
8[13C]inosine	0	167.99265	242.35605	0	0	90.8019	0	0	0	493.72465	1346.8297	753.1594
9[13C]inosine	0	0	0	0	0	0	0	0	0	0	0	0
10[13C]inosine	0	0	294.45115	85.1906	275.998	0	0	176.68615	0	1405.31165	18249.43985	1764.5049
serine	740480.1905	886525.3803	669431.3313	858564.9849	883757.5837	923396.3543	1026456.226	847881.4286	1019394.132	1091891.048	1248137.127	1398394.5
1[13C]serine	109320.8489	135221.8963	134107.6194	120511.4238	107642.946	148335.5784	99583.4964	89095.8006	103160.469	42262.39685	39630.4778	52723.91085
2[13C]serine	41286.05295	44633.75635	44735.2815	35417.7055	30698.24375	41511.42105	26521.95765	21583.2522	28995.4835	417.64125	0	291.848
3[13C]serine	38325.7757	42196.99605	46160.5715	41227.95075	35227.58635	46363.8666	36220.86045	41193.1257	44436.19675	1928.53045	1917.88935	3046.6042
proline	17539170.15	19735617.34	17474342.53	17635825.95	17095454.31	22327687.57	10633505.09	9830055.6	10699759.35	8308552.978	9035947.131	8461328.217
1[13C]proline	1225711.832	1428702.334	1242389.106	1189197.565	1138201.886	1510907.308	613118.6542	577905.3026	615733.2932	469399.4277	507122.9793	473145.17
2[13C]proline	942142.1363	1050063.498	1130550.651	682449.237	625699.1991	907742.6257	102939.6695	93554.9142	89789.21455	5560.13445	3122.9133	2143.56065
3[13C]proline	118479.9601	145231.378	150043.0536	70014.2768	60444.4448	107300.2596	966.73785	1627.2248	2875.7355	1065.0241	0	0
4[13C]proline	40925.89645	52803.52705	81662.1751	15597.5492	12891.1575	27731.71355	0	0	0	0	0	0
5[13C]proline	481.48425	323.968	4334.9632	501.38205	0	701.4033	0	1005.2846	0	0	0	0
aspartate	1465517.508	1668921.269	1273742.664	1713780.562	1809914.609	1991611.624	1633522.43	1420789.879	1700310.176	2161672.716	2236636.987	2047082.164
1[13C]aspartate	115205.4577	141781.0034	109310.5471	117351.2203	129904.2365	162647.342	134683.3447	110605.5582	131770.8395	98470.64145	102277.2051	97049.4215
2[13C]aspartate	104644.619	123067.1584	119035.9537	96940.8157	97424.82345	121537.968	84159.38165	68612.42285	75354.4447	11049.1062	9886.12405	10265.5944
3[13C]aspartate	67302.3035	68365.9971	69375.60285	63102.8239	59447.2409	91111.09865	47508.30825	48542.9134	53449.51265	13547.02	11935.53655	11886.01135
4[13C]aspartate	686.2791	983.52285	2371.3211	501.838	371.0577	1767.3084	538.36575	782.26395	0	0	0	0
glutamine	52131205.55	61492722.07	42957057.73	56246379.73	59962934.52	58031926.33	69387920.25	56740462.03	68636525.29	78867797.02	88486554.16	91818338.45
1[13C]glutamine	2955624.892	3493202.809	2451364.522	3181082.916	3391312.312	3295381.415	3940414.804	3175529.308	3885344.142	4467092.131	5015667.9	5223740.685
2[13C]glutamine	53797.5098	57986.20545	47174.0464	52728.962	56769.9201	58029.55325	66898.86195	60048.6514	71809.8743	74778.4632	78144.07175	83321.29435
3[13C]glutamine	0	470.8805	68.0883	305.496	116.753	0	0	0	0	805.56105	2092.99235	785.431
4[13C]glutamine	0	0	0	0	0	0	0	0	0	0	0	0
5[13C]glutamine	0	0	0	0	0	0	0	0	0	0	0	0
glutamate	19487398.42	22982551.77	20278738.84	19264552.66	18883316.3	25222426.71	20223607.87	18384093.84	21253709.8	16541866.61	15989634.57	14662548.71
1[13C]glutamate	1733280.759	2055170.564	1792751.194	1672300.012	1611377.046	2220162.192	1824460.61	1628659.949	1811697.252	1017735.565	994795.4619	935037.5613
2[13C]glutamate	2191852.844	2718727.767	2543913.474	1777575.475	1646626.101	2448336.694	1788436.004	1572550.777	1688381.171	272176.795	268369.7308	244758.8609
3[13C]glutamate	348679.2874	423491.2189	415488.5415	267485.6935	241731.4424	349265.6424	308341.8787	276598.0798	281135.6757	70721.2247	74486.38635	82645.26255
4[13C]glutamate	135757.5218	170200.875	197130.515	91171.77765	71192.60195	122719.9731	103851.1005	85957.4551	81733.9786	790.6451	2851.6709	0
5[13C]glutamate	28634.4674	35168.76585	41125.6131	18118.6388	16211.84485	21747.81125	16210.7371	19273.92585	18002.18515	274.92465	0	0
methionine	3126848.065	4120368.187	2504281.57	3674599.059	4534007.589	3608614.638	4480410.88	2413238.811	4422965.888	7584528.972	6779118.917	8489823.517
1[13C]methionine	181389.7902	235605.709	146137.3492	204152.6164	253646.3544	205590.2035	251380.262	131383.053	244381.9033	412777.3327	359740.0612	458515.0612
2[13C]methionine	1853.6286	420.468	778.4386	943.012	966.924	0	475.15	0	1264.87475	4450.56645	2705.2439	4408.6817
3[13C]methionine	3873.28625	4792.2583	3914.6718	5280.3714	2477.538	4567.005	1423.11985	282.61	434.83165	0	0	0
4[13C]methionine	66847.7537	85566.75915	81120.1316	66282.97825	69603.40205	64052.1644	57833.2693	31043.26705	56036.79065	3685.11485	2915.0333	4262.30525
5[13C]methionine	0	0	0	0	0	0	0	0	0	0	0	0
glutathione	468480.1998	489194.1515	606601.6734	393596.9747	379070.7137	440180.9988	924382.6985	936362.2221	1161464.246	687484.425	714380.299	839880.364
1[13C]glutathione	50061.7361	56503.4125	66439.12275	41379.2596	41398.97985	44794.8436	101347.6667	103330.7407	127495.1526	75287.04875	72297.54995	84388.01195
2[13C]glutathione	31485.49485	29500.0363	37962.7098	25170.14545	25555.9491	28549.7472	51419.27855	50511.2834	61706.6331	35994.89875	41233.1443	45424.48265
3[13C]glutathione	2690.30285	603.452	2868.54285	1095.60555	591.5637	1064.93055	5641.45825	4585.96965	7920.3403	1661.73735	690.4443	2655.11415
4[13C]glutathione	900.69525	0	2757.1243	1324.0812	358.51725	1296.75975	2521.64445	1337.69545	4989.7542	0	0	737.67135
5[13C]glutathione	0	0	0	0	0	0	0	0	471.0927	0	0	0
6[13C]glutathione	0	0	0	0	0	0	0	0	0	0	0	0
7[13C]glutathione	0	0	0	0	0	0	0	0	0	0	0	0
8[13C]glutathione	16970.38115	6792.0996	0	10727.4062	29740.51275	5949.9141	41139.7444	10526.76515	7972.23375	22126.4355	82931.58895	61895.71715
9[13C]glutathione	180.79565	546.334	179.6661	1601.1056	2516.4437	948.27575	1940.366	3926.8232	0	1870.3074	6092.19195	2438.8901
10[13C]glutathione	0	0	0	0	0	0	0	0	0	0	0	0
NAD+_pos	101372.5122	120589.5269	93948.115	117113.4734	112337.5824	144604.4789	74698.26705	81594.9023	90239.53035	156761.9303	131911.3521	101405.7712
1[13C]NAD+_pos	31181.6302	38985.14355	30050.0909	35655.01595	33720.1583	48809.62035	25290.2638	33814.7602	35448.7296	148846.2724	110930.9858	95246.656
2[13C]NAD+_pos	69855.7018	82043.444	57808.8369	79125.15985	76221.93825	98206.2389	52600.3855	72947.57165	86484.2445	315722.0838	263379.8843	220417.7786
3[13C]NAD+_pos	49959.64145	61817.3639	50584.255	72177.64915	65800.2801	78085.60265	38097.29415	45622.6172	53208.18185	194833.3988	154897.8696	136531.1581
4[13C]NAD+_pos	123181.2996	150180.2972	117983.1152	136645.6502	127910.5258	163607.7838	77263.2798	88134.0504	107491.7373	253869.2849	210906.7895	181818.3904
5[13C]NAD+_pos	459970.3862	511483.8919	435745.2687	513941.2253	463317.9829	599749.5794	381277.4883	428502.8367	480020.0966	385128.7919	300378.3519	260268.8665
6[13C]NAD+_pos	221085.068	250561.9655	204239.7689	257242.1152	228413.0146	301088.619	168529.9093	168502.436	193005.6653	202193.7346	152612.3413	146014.397
7[13C]NAD+_pos	661547.4634	797209.6191	638536.8396	671898.2892	613074.2624	832777.9568	523192.9277	473044.752	538039.195	376444.6526	287008.3517	256706.2587
8[13C]NAD+_pos	340392.2831	401689.4791	359392.897	400776.1004	353971.8645	503544.9549	296162.425	288396.984	322063.7325	120233.2181	97753.69095	89910.1082
9[13C]NAD+_pos	385428.7947	467652.6408	403019.4346	409915.1883	376136.2579	496495.2416	341193.9912	316425.4177	356615.5349	103754.0617	85911.52195	75991.2839
10[13C]NAD+_pos	2436857.349	2826401.727	2479287.505	2182676.583	1936460.461	2609226.714	2249289.653	2135033.535	2360147.369	443421.4463	360271.2641	316112.5244
11[13C]NAD+_pos	378172.2571	427341.4394	401746.9633	321360.6812	290108.3631	383111.6984	323179.8028	312077.5804	339580.7853	61611.2602	48415.76965	44469.42415
12[13C]NAD+_pos	94076.25295	112616.145	109914.5657	87476.7592	75995.3036	103191.5702	83340.49105	80104.7074	93439.351	12655.389	10558.0212	10516.57475
13[13C]NAD+_pos	10136.9035	13178.18775	15696.3922	8711.15045	10099.84935	13355.92555	11147.572	12225.79555	10182.8635	1957.2752	1439.6204	764.96055
14[13C]NAD+_pos	0	376.49625	360.154	0	0	0	0	389.77965	407.474	0	0	0
15[13C]NAD+_pos	0	0	0	0	0	0	0	0	0	0	0	0
16[13C]NAD+_pos	0	0	0	0	0	0	0	0	0	0	0	0
17[13C]NAD+_pos	0	0	0	0	0	0	0	0	0	0	0	0
18[13C]NAD+_pos	0	0	0	0	0	0	0	0	0	0	0	0
19[13C]NAD+_pos	0	0	0	0	0	0	0	0	0	0	0	0
20[13C]NAD+_pos	0	0	0	0	0	0	0	0	0	0	0	0
21[13C]NAD+_pos	0	0	0	0	0	0	0	0	0	591.18735	0	0
alanine	278300.4028	278004.6082	320288.9732	353486.829	285927.1085	442653.6619	184456.4806	204094.6444	182740.1448	122493.2804	119348.0971	124668.0755
1[13C]alanine	6276.9932	2883.8879	7405.78665	7431.1447	2900.9947	11470.5756	1331.05885	2307.5283	443.3042	0	0	0
2[13C]alanine	1717.7528	780.6401	5604.9351	2555.2908	319.822	3294.40085	0	117.77455	0	0	0	0
3[13C]alanine	147972.4253	140246.4395	227118.6531	123523.5082	103573.9468	184407.4194	77962.5931	89911.19215	87620.95135	859.11405	1281.2315	7855.74215
